# Mind over Malignancy: A Systematic Review and Meta-Analysis of Psychological Distress, Coping, and Therapeutic Interventions in Oncology

**DOI:** 10.3390/medicina61061086

**Published:** 2025-06-13

**Authors:** Ana Maria Paslaru, Alina Plesea-Condratovici, Lavinia-Alexandra Moroianu, Oana-Maria Isailă, Laura Florentina Rebegea, Liliana Lacramioara Pavel, Anamaria Ciubară

**Affiliations:** 1 Doctoral School of Biomedical Sciences, “Dunărea de Jos” University, 800201 Galați, Romania; annapaslaru@gmail.com; 2 Medical Department, Faculty of Medicine and Pharmacy, Dunărea de Jos University, 800201 Galați, Romania; alina.plesea@ugal.ro; 3 Department of Pharmaceutical Sciences, Faculty of Medicine and Pharmacy, Dunărea de Jos University, 800201 Galați, Romania; doctorpavel2012@yahoo.com; 4 Department of Legal Medicine and Bioethics, Faculty of Dental Medicine, “Carol Davila” University of Medicine and Pharmacy, 020021 Bucharest, Romania; 5 Clinical Medical Department, Faculty of Medicine and Pharmacy, Dunărea de Jos University, 800201 Galați, Romania; laura.rebegea@ugal.ro (L.F.R.); anamburlea@yahoo.com (A.C.)

**Keywords:** cancer, psychological distress, depression, psycho-oncology, anxiety, PTSD, Cognitive Behavioral Therapy, coping strategies

## Abstract

*Background and Objectives:* Psychological distress is a pervasive yet often undertreated aspect of the cancer experience, contributing to reduced quality of life, poorer treatment adherence, and worse health outcomes. This systematic review and meta-analysis evaluated the efficacy of non-pharmacological, evidence-based psychological interventions on distress, depression, anxiety, coping capacity, and quality of life in adult cancer patients. Interventions were grouped into three domains: structured psychotherapeutic therapies (e.g., Cognitive Behavioral Therapy [CBT], Acceptance and Commitment Therapy [ACT], Meaning-Centered Psychotherapy [MCP]); mindfulness and stress reduction programs (e.g., Mindfulness-Based Stress Reduction [MBSR], Mindfulness-Based Cognitive Therapy [MBCT]); and coping and resilience-enhancing modalities (e.g., Promoting Resilience in Stress Management [PRISM], expressive writing). *Materials and Methods:* Following PRISMA guidelines, 42 randomized controlled trials published between 2015 and 2025 were included. A stratified meta-analytic approach calculated pooled standardized mean differences for each intervention class and outcome. Heterogeneity, subgroup, and moderator analyses explored drivers of effect variability. *Results:* Structured psychotherapeutic interventions yielded the largest effects, especially for depression. Mindfulness-based interventions produced moderate but significant improvements in distress and emotional regulation. Coping and resilience programs provided smaller yet statistically significant gains in adaptive coping. Between-study heterogeneity was moderate, partly explained by intervention type, delivery modality, and cancer subtype. *Conclusions:* These findings support integrating psychosocial care into standard oncology protocols and endorse its routine implementation as a core component of comprehensive cancer treatment.

## 1. Introduction

### 1.1. Main Review Question

Cancer is not solely a biological disease but also an existential and psychological crisis. A cancer diagnosis and its subsequent trajectory through treatment, survivorship, or end-of-life care often involve high rates of psychological comorbidities–depression, generalized anxiety disorder, adjustment disorders, distress syndromes, and post-traumatic stress symptoms–across the oncological continuum. These conditions negatively impact treatment adherence, functional status, and quality of life and may influence overall prognosis. Psychological distress typically peaks at critical transitions such as diagnosis, recurrence, metastasis, and terminal phases, when emotional regulation, sense of meaning, and perceived control can become destabilized.

Despite growing recognition of cancer’s psychological impact, psychosocial care remains unevenly implemented worldwide. Variation persists in therapeutic orientation, resource allocation, clinical integration, and patient access. Concurrently, research on non-pharmacological interventions has expanded to include structured psychotherapies (Cognitive Behavioral Therapy [CBT], Acceptance and Commitment Therapy [ACT], Interpersonal Therapy [IPT], Meaning-Centered Psychotherapy [MCP]); mindfulness-based and stress reduction programs (Mindfulness-Based Stress Reduction [MBSR], Mindfulness-Based Cognitive Therapy [MBCT]); and resilience-building models (Promoting Resilience in Stress Management [PRISM]). Additional approaches include dyadic interventions, psychoeducation, supportive-existential counseling, and hybrid programs that combine cognitive, affective, and relational components [1,2,3]. However, the heterogeneity of interventions, variability in methodological rigor, and inconsistency of outcome measures across studies complicate efforts to draw generalizable conclusions. A systematic synthesis is therefore warranted to determine which modalities yield the greatest benefit for specific populations, cancer types, and psychological endpoints.

This systematic review and stratified meta-analysis critically examines the efficacy of non-pharmacological psychological interventions on depression, anxiety, psychological distress, coping capacity, and quality of life in adult cancer patients. We included randomized controlled trials (RCTs) published between 2015 and 2025 to reflect a contemporary evidence base. Interventions were categorized into three theoretically and methodologically distinct domains: structured psychotherapeutic approaches (CBT, ACT, MCP, IPT); mindfulness-based and stress reduction interventions (MBSR, MBCT); and coping and resilience-enhancing programs (e.g., PRISM). These categories were chosen based on conceptual distinctiveness, empirical prevalence, and methodological maturity. The review addresses the following question: To what extent do structured psychotherapeutic therapies, mindfulness-based stress reduction interventions, and coping/resilience-enhancing programs improve depression, anxiety, psychological distress, coping capacity, and quality of life in adult cancer patients, as demonstrated in RCTs from 2015 to 2025? We tested these hypotheses, which guided our eligibility criteria, risk-of-bias assessment, quantitative synthesis, and interpretation of subgroup and moderator analyses:

i. Null Hypothesis (H_0_): Structured psychotherapeutic, mindfulness-based, and resilience-enhancing interventions yield no statistically significant improvements in depression, anxiety, distress, coping capacity, or quality of life.
ii. Alternative Hypothesis (H_1_): Structured psychotherapeutic approaches (e.g., CBT, ACT, MCP), mindfulness-based therapies (e.g., MBSR, MBCT), and coping/resilience-enhancing programs (e.g., PRISM) are associated with statistically and clinically significant improvements in one or more of these outcomes.

### 1.2. Background

Over the past two decades, cancer care has shifted from a strictly biomedical model to an integrative biopsychosocial framework, driven by empirical data and patient-reported outcomes highlighting the psychological burden of cancer. Emotional distress–far from peripheral–critically affects physical and psychosocial trajectories. Untreated symptoms such as anxiety, depression, existential distress, and maladaptive coping can undermine treatment adherence, impede recovery, reduce engagement, and diminish quality of life. Consequently, the American Society of Clinical Oncology (ASCO), the National Comprehensive Cancer Network (NCCN), and the World Health Organization (WHO) all stress integrating psychological support into standard oncology protocols [4,5]. At the same time, the literature has expanded to include diverse non-pharmacological interventions: structured psychotherapies grounded in cognitive-behavioral theory, mindfulness-based protocols for emotional regulation and attention control, resilience-based coping strategies that promote adaptation and growth, and more recently, artificial intelligence-driven digital mental health tools–such as virtual coaches delivering cognitive-behavioral modules, smartphone applications using machine learning to tailor psychoeducation, and conversational agents providing real-time symptom monitoring.

Despite their promise, these AI-based approaches remain in early stages of evaluation, adding to an already heterogeneous field. Taken together, these studies remain conceptually fragmented, methodologically varied, and often produce inconsistent findings–underscoring the urgent need for a comprehensive, stratified meta-analytic synthesis [6,7].

#### 1.2.1. Conceptual Convergence of Psychotherapeutic Interventions in Oncology

This subsubsection addresses the theoretical underpinnings that unite diverse psychological interventions under common conceptual umbrellas. Despite their surface-level distinctions, structured psychotherapies, mindfulness-based methods, and resilience-building approaches share foundational mechanisms of action relevant to psychological recovery in oncology. These include modulation of cognitive distortions, enhancement of present-moment awareness, cultivation of psychological flexibility, and reinforcement of meaning-making processes in the context of existential threat.

For instance, Cognitive Behavioral Therapy (CBT) aims to restructure maladaptive beliefs and promote adaptive behavior, while Acceptance and Commitment Therapy (ACT) emphasizes value-guided living through acceptance and cognitive defusion. In parallel, Mindfulness-Based Stress Reduction (MBSR) fosters non-judgmental attention to internal experience, thereby reducing emotional reactivity and psychological rumination. Finally, coping and resilience interventions often integrate psychoeducational and skill-based components designed to strengthen problem-solving, emotional regulation, and goal setting in the face of medical uncertainty and loss [8,9,10].

Collectively, these interventions target transdiagnostic emotional vulnerabilities–such as helplessness, avoidance, catastrophizing, and demoralization–that are prevalent among cancer patients and predictive of poorer outcomes. The convergence of therapeutic mechanisms across intervention types provides a compelling rationale for their comparative evaluation and integration in a unified meta-analytic framework.

#### 1.2.2. Methodological Gaps and Challenges in the Current Literature

Despite significant growth in psychosocial oncology, methodological inconsistencies continue to undermine the reliability, generalizability, and clinical translation of findings. In randomized controlled trials (RCTs) evaluating psychological interventions, outcome measures vary widely: depression, anxiety, distress, and quality of life are assessed using instruments such as the Hospital Anxiety and Depression Scale (HADS), Patient Health Questionnaire-9 (PHQ-9), Generalized Anxiety Disorder-7 (GAD-7), Distress Thermometer, and Functional Assessment of Cancer Therapy (FACT) scales, among others. Such heterogeneity complicates cross-study comparisons and reduces the precision of pooled effect sizes [11,12,13,14,15].

Intervention fidelity also differs substantially. Some studies implement manualized protocols delivered by trained mental health professionals, while others rely on brief or self-guided formats with minimal therapist involvement or quality assurance. This variability can moderate outcomes but is often underreported or omitted. Sample size limitations further challenge the field: many RCTs are underpowered to detect small-to-moderate effects, especially in subgroups defined by cancer type, treatment phase, or demographic factors. Small samples not only increase the risk of Type II errors but also limit the applicability of results to broader oncology populations. Moreover, the predominance of female participants–particularly in mindfulness trials for breast cancer–further restricts ecological validity. Control conditions lack standardization as well, ranging from usual care and waitlist controls to attention-matched or active comparators, each influencing internal validity and effect size interpretation differently. Trials with active controls often report smaller between-group differences yet provide more rigorous evidence of treatment specificity. Inconsistent control designs hinder comparability and increase heterogeneity in effect estimates.

Additional challenges include short follow-up durations that preclude assessment of long-term effects; non-reporting or selective reporting of secondary outcomes; insufficient blinding of outcome assessors–raising detection bias concerns; and inadequate description of randomization procedures–undermining confidence in trial integrity. Altogether, these methodological gaps demand cautious interpretation of existing data and highlight the need for a structured, stratified, quality-weighted meta-analysis to parse intervention effects by study quality, measurement tools, sample demographics, and control type. Addressing these limitations is essential for generating more rigorous, replicable, and clinically actionable evidence.

#### 1.2.3. Temporal Relevance and Evidence Currency in Psychosocial Oncology

The oncology landscape is advancing rapidly with innovations in diagnostics, treatments, supportive care, and survivorship models. Consequently, the temporal relevance of psychological intervention research is crucial for applicability in modern practice. Psychological needs and treatment contexts now differ markedly from those of earlier cohorts due to improved prognoses, evolving side-effect profiles, and transformed healthcare delivery–especially via digital health, telepsychology, and integrative pathways. Most psychosocial oncology meta-analyses rely on pre-2015 studies, thereby underrepresenting recent developments. Emerging modalities–digital cognitive-behavioral therapy, tele-delivered mindfulness interventions, and resilience-focused mobile platforms–have grown prominent yet remain scarce in earlier reviews. The COVID-19 pandemic further accelerated a shift in delivery methods, underscoring the need to analyze post-2015 data reflecting current constraints and opportunities [16,17].

This review addresses that gap by including randomized controlled trials from 2015 to 2025, capturing advances such as (a) precision psychosocial interventions tailored to cancer subtypes; (b) widespread implementation of psychological screening in oncology; (c) cross-cultural trials in underrepresented regions (Asia, Eastern Europe, Middle East); and (d) frameworks integrating third-wave cognitive therapies, trauma-informed care, and existential-experiential approaches. By focusing on contemporary evidence, our synthesis offers more ecologically valid insights into non-pharmacological interventions in today’s clinical contexts. It incorporates updated outcome measures, diverse participants, and hybrid care models poised to inform guidelines and policy. This temporal precision enhances applicability, actionability, and forward compatibility, benefiting clinicians, health systems, and policy–makers aiming to optimize psychological care in oncology.

### 1.3. Rationale

#### 1.3.1. Clinical Imperatives and Unmet Needs

The rationale for this review stems from both clinical urgency and scientific opportunity. Despite advances in cancer treatments and supportive care, patients’ psychological well-being remains under-addressed. Feelings of overwhelm, despair, and loss of control can progress to diagnosable mental health disorders, negatively affecting treatment outcomes and survivorship. Although psychological morbidity in cancer is well documented, integrating evidence-based interventions into oncology is limited by inconsistent protocols, variable professional training, scarce resources, and underestimation of distress as a modifiable risk factor. Clinical guidelines often provide general recommendations without stratified, evidence-based comparisons of intervention types, formats, or mechanisms.

Existing reviews have struggled to keep pace with the field’s evolution. Prior syntheses often conflate diverse interventions or focus narrowly on a single modality, neglecting the broader therapeutic ecosystem. Moreover, meta-analyses rarely disaggregate findings by intervention class, cancer type, or outcome domain, producing pooled estimates that mask clinically meaningful distinctions. This review addresses these gaps by offering a systematic, stratified, and mechanism-aware evaluation of recent non-pharmacological interventions in adult cancer populations. Its unique contributions include: (a) categorizing interventions into coherent domains–structured psychotherapy, mindfulness, and coping-based programs; (b) including only high-quality randomized controlled trials from 2015 to 2025; and (c) applying quantitative synthesis to compare differential effects across outcomes, populations, and methodologies. By filling these clinical and academic voids, this review supports clinicians’ decision-making and guides future psychosocial oncology research.

#### 1.3.2. Theoretical Foundations and Empirical Rationale for the Review

The conceptual framework for this review draws on the biopsychosocial model, stress-appraisal-coping theory, and third-wave cognitive-behavioral paradigms, which together suggest that cancer-related distress arises from interactions among biological vulnerability, threat appraisals, maladaptive coping, and environmental stressors. Thus, the psychological sequelae of cancer are predictable, measurable, and modifiable via structured psychosocial interventions. Engel’s biopsychosocial model underpins the rationale for addressing mental health in medical settings. In oncology, it encompasses existential and spiritual dimensions, acknowledging cancer’s disruptive impact on meaning, identity, and life continuity. These disruptions often manifest as persistent affective symptoms and fragmentation of psychological integration that pharmacological or purely somatic treatments cannot resolve [18,19,20].

Building on this, Lazarus and Folkman’s stress-appraisal-coping theory explains how individuals interpret and respond emotionally to cancer-related stressors. Primary appraisals (perceived threat or challenge) and secondary appraisals (perceived coping resources) critically determine emotional outcomes. Interventions that modify maladaptive appraisals or bolster coping self-efficacy directly target these mechanisms [21,22]. Third-wave psychotherapies–such as Acceptance and Commitment Therapy (ACT), Mindfulness-Based Cognitive Therapy (MBCT), and Compassion-Focused Therapy (CFT)–shift the focus from symptom elimination toward psychological flexibility, values-oriented action, and present-moment engagement. Their theoretical and empirical relevance to life-threatening illness and existential disruption makes them particularly suitable for integration into cancer care [23,24].

Empirically, numerous studies show that psychological interventions produce significant reductions in distress, anxiety, depression, and trauma symptoms among cancer patients. However, previous reviews often aggregate dissimilar interventions or overlook mechanistic specificity, limiting interpretability. Few syntheses employ stratified meta-analytic methods that compare intervention classes, populations, and methodological quality. This review is therefore both theoretically grounded and empirically necessitated. By leveraging validated psychological theories and addressing analytical gaps, it enhances the precision, coherence, and translational value of conclusions regarding the effectiveness of non-pharmacological interventions in psychosocial oncology.

### 1.4. Objectives and Methodological Framework

The objective of this systematic review and stratified meta-analysis was to evaluate the efficacy and clinical significance of structured psychotherapeutic interventions (Cognitive Behavioral Therapy, Acceptance and Commitment Therapy, Meaning-Centered Psychotherapy), mindfulness-based and stress reduction programs (Mindfulness-Based Stress Reduction, Mindfulness-Based Cognitive Therapy), and coping- and resilience-enhancing interventions (self-guided tools, psychoeducation, strengths-based frameworks) on depression, anxiety, psychological distress, coping capacity, and quality of life in adults with cancer. We examined whether these approaches reduced depression, anxiety, and distress and improved coping capacity and quality of life versus control conditions.

Interventions were classified into three domains for conceptual clarity: Structured Psychotherapeutic Interventions; Mindfulness-Based and Stress Reduction Programs; and Coping and Resilience-Enhancing Interventions. Following PRISMA guidelines, we included randomized controlled trials published between 2015 and 2025 to ensure temporal relevance and rigor. Data synthesis used a multi-phase strategy: first, a descriptive summary of study characteristics (sample size, cancer type, intervention, duration, outcomes; see Section 3.1 and Table S4); second, stratified random-effects meta-analyses computed pooled effect sizes for each outcome across the three intervention categories; and third, meta-regression and subgroup analyses evaluated moderators (cancer type, study quality, control condition). A narrative synthesis interpreted heterogeneity, contextualized quantitative findings, and integrated results across diverse trial designs. Evidence strength was appraised using Grading of Recommendations Assessment, Development and Evaluation (GRADE) criteria where applicable [25]. This rigorous framework ensures the findings are statistically valid, clinically actionable, and theoretically informative, guiding both oncology practice and future psychosocial research.

## 2. Materials and Methods

### 2.1. Protocol and Registration

This systematic review and meta-analysis was conducted in alignment with the Preferred Reporting Items for Systematic Reviews and Meta-Analyses (PRISMA) 2020 statement, which provides structured guidance for transparency, methodological rigor, and comprehensive reporting. While the review followed all PRISMA-based procedures and analytic standards, it was not prospectively registered in PROSPERO or any equivalent protocol registry [26]. Despite the absence of formal registration, all methodological parameters–including eligibility criteria, data extraction procedures, outcome definitions, and statistical models–were predefined by the research team prior to data synthesis. These procedures were consistently applied across all included studies and documented in detail throughout the manuscript and Supplementary Materials. The decision not to pursue protocol registration was based on project timelines and scope but does not compromise the reproducibility, transparency, or integrity of the review process.

### 2.2. Inclusion Parameters and Screening Logic

**Figure 1:**
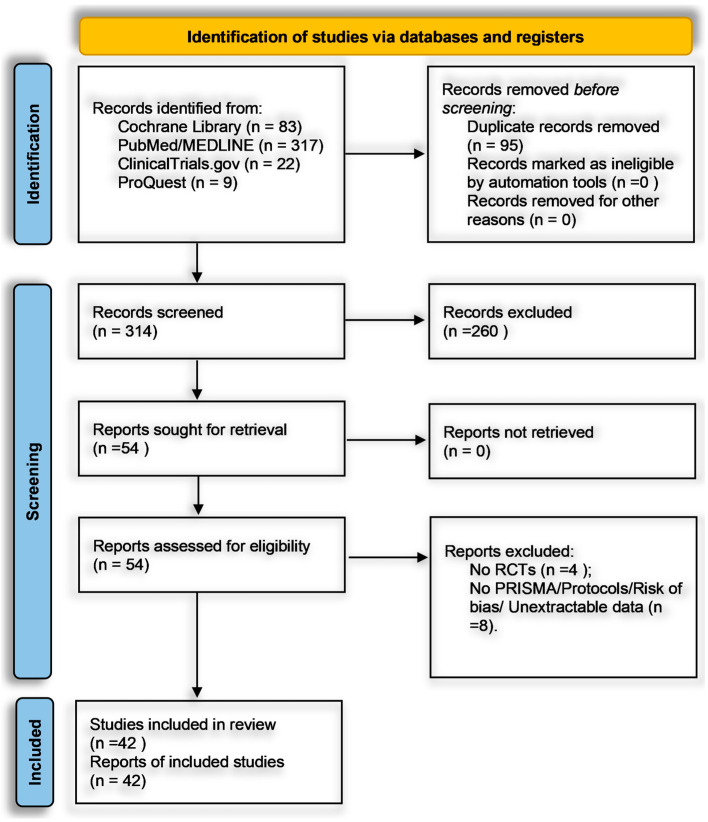
PRISMA 2020 flow diagram depicting study identification, screening, and inclusion process.

Figure 1 illustrates our PRISMA 2020-guided process for identifying, screening, and including studies targeting the efficacy of structured, non-pharmacological psychological interventions in adult cancer populations [27]. We retrieved 504 records from the Cochrane Central Register of Controlled Trials (*n* = 83), PubMed/MEDLINE (*n* = 317), ClinicalTrials.gov (*n* = 22), and ProQuest Dissertations and Theses (*n* = 9) and imported them into Zotero. An automated deduplication step removed 95 duplicates, leaving 409 unique records for title and abstract screening. During this phase, two independent reviewers evaluated each record against our PICO framework-adult cancer patients (≥18 years), structured non-pharmacological psychological interventions (structured psychotherapeutic approaches including Cognitive Behavioral Therapy, Acceptance and Commitment Therapy, Interpersonal Therapy, and Meaning-Centered Psychotherapy; mindfulness-based/stress-reduction protocols such as Mindfulness-Based Stress Reduction, Mindfulness-Based Cognitive Therapy, guided meditation, or relaxation; or coping- and resilience-oriented strategies like Promoting Resilience in Stress Management, coping skills training, or expressive therapies), comparator conditions (usual care, waitlist, attention-matched, or active psychological control), and validated psychometric outcomes such as the Hospital Anxiety and Depression Scale (HADS), Patient Health Questionnaire-9 (PHQ-9), Generalized Anxiety Disorder-7 (GAD-7), Depression Anxiety Stress Scales-21 (DASS-21), Patient-Reported Outcomes Measurement Information System (PROMIS), Connor-Davidson Resilience Scale (CD-RISC), Functional Assessment of Cancer Therapy-General (FACT-G), Posttraumatic Growth Inventory (PTGI), Five Facet Mindfulness Questionnaire (FFMQ), or Pittsburgh Sleep Quality Index (PSQI).

Records were excluded at this stage (*n* = 260) for the following specific reasons: 110 non-RCT designs (e.g., observational or qualitative reports), 84 interventions lacking an isolatable psychological component (e.g., purely pharmacological or physical rehabilitation studies), 32 non-adult or non-cancer populations (e.g., pediatric or caregiver-only cohorts), 18 publications outside the 2015–2025 window, and 16 records with no extractable psychometric data (e.g., no standardized instruments or only qualitative outcomes). Any screening disagreements were resolved by consensus or, if necessary, by a third reviewer. After excluding those 260 records, 149 articles advanced to full-text review. Two reviewers independently confirmed eligibility, ensuring each study employed an RCT design in adult oncology, implemented one of the three predefined intervention domains, included a valid comparator, and reported outcomes in statistically extractable formats (e.g., standardized mean differences, mean differences, odds ratios, relative risks, or hazard ratios with standard deviations or 95% confidence intervals). Of these 149, 95 were excluded for lacking an RCT design (*n* = 54), absence of a standardized outcome measure (*n* = 28), multi-component interventions without isolatable psychological data (*n* = 8), or missing extractable statistical data (*n* = 5). No full texts were inaccessible. The remaining 54 underwent further screening: four were removed for non-randomized or pilot feasibility designs lacking comparators, and eight were excluded for failing to meet PRISMA reporting or risk-of-bias documentation standards. Ultimately, 42 RCTs satisfied all criteria and were included in the narrative synthesis and meta-analysis.

Throughout screening, Zotero managed references, and Covidence tracked inclusion/exclusion decisions with time-stamped entries. Full-text PDFs were stored in a secure, organized repository, and data extraction was conducted using a pre-piloted Excel form. Two reviewers independently extracted key variables–sample size, cancer type, intervention details, comparator, outcomes, and statistical results–while a third reviewer cross-checked 20% of extractions for accuracy; any discrepancies were resolved by consensus or senior adjudication. The Cochrane Risk of Bias 2.0 tool was applied by two reviewers to appraise each trial’s methodological rigor, with any disagreements adjudicated by a third reviewer, and summary visuals generated using ROBVIS. Two reviewers independently applied the Grading of Recommendations Assessment, Development, and Evaluation (GRADE) framework to determine the certainty of evidence for each outcome domain; discrepancies were resolved by consensus or third-party adjudication. Data management included automatic and manual deduplication in Zotero and full-text organization in cloud folders labeled “Screened”, “Eligible”, and “Excluded”. This comprehensive, dual-reviewer approach–combined with explicit inclusion/exclusion reasons, PICO alignment, dedicated software tools, and rigorous risk-of-bias and GRADE assessment–ensures transparency, reproducibility, and methodological rigor in synthesizing high-quality evidence on non-pharmacological psychological interventions in adult cancer care.

### 2.3. Psychological Intervention Typologies and Inclusion Rationale

Rather than treating all non-pharmacological therapies as a single group, we organized interventions into three distinct domains–structured psychotherapeutic, mindfulness-based and stress-reduction, and coping and resilience–enhancing–based on their core mechanisms, theoretical foundations, and delivery formats. This stratification reflects advances in mechanism-based psychotherapy research by isolating active ingredients and clarifying treatment pathways. Each category was required to be manualized or protocol-driven, conceptually coherent, and delivered as a standalone program to ensure that observed effects could be attributed to the intended psychological mechanism. As shown in Table 1, structured psychotherapeutic interventions (15 RCTs) produced a pooled Hedges’ g of −0.51 (95% CI −1.06 to 0.04) for depression, anxiety, and distress; mindfulness-based programs (13 RCTs) yielded −0.29 (95% CI −0.70 to 0.11) for distress, anxiety, fatigue, and emotional regulation; and coping and resilience-enhancing approaches (14 RCTs) demonstrated −0.43 (95% CI −0.81 to −0.05) for anxiety, depression, coping skills, and psychological growth. By preserving mechanistic specificity and replicability, this framework enables clearer statistical comparisons and supports clinical translation in oncological settings.

### 2.4. Analytical Inference Models and Meta-Analytic Parameters

To evaluate psychological interventions in oncology, we used a three-phase stratified meta-analytic approach that enhances validity and interpretive clarity amid trial heterogeneity. In Phase 1, random-effects models synthesized Hedges’ g effect sizes within each intervention category (structured psychotherapeutic, mindfulness-based, and coping/resilience-focused) via restricted maximum likelihood (REML) estimation of between-study variance (τ^2^). Individual study weights were assigned by inverse variance, and forest plots displayed both individual and pooled effects with 95% confidence intervals. Heterogeneity was assessed using Cochran’s Q, I^2^ (with values above 75% indicating substantial inconsistency), and τ^2^; prediction intervals estimated the range of true effects for future trials. Phase 2 involved subgroup analysis within structured psychotherapeutic interventions, comparing Cognitive Behavioral Therapy (CBT) versus supportive/expressive modalities. A Z–test evaluated differences between pooled standardized mean differences (SMDs), allowing us to identify whether therapeutic orientation drove outcome variability. Phase 3 employed meta-regression to test intervention category as a categorical moderator of effect size. Weighted least squares regression, using inverse variance weights (1/SE^2^), produced regression coefficients (β) indicating the direction and magnitude of association, and an F-statistic assessed the overall model fit. Diagnostics included residual analysis, R^2^ analogs for variance explained, and collinearity checks. Egger’s test and funnel plots evaluated publication bias and small-study effects. By integrating pooled synthesis, subgroup contrasts, and moderator analyses, this layered design provides a rigorous, clinically meaningful evaluation of intervention efficacy across psychological care modalities in cancer.

### 2.5. Software Ecosystem and Statistical Implementation Infrastructure

This review employed a coordinated software ecosystem to ensure transparency, accuracy, and reproducibility. Zotero was used for reference management, citation tracking, and collaborative screening, allowing systematic import, tagging, and annotation of eligible studies. Risk-of-bias assessments were visualized via ROBVIS, which graphically displays Cochrane bias domains (randomization, allocation concealment, blinding, attrition, selective reporting) to enhance methodological clarity. Core meta-analytic procedures–pooled effect size calculation, heterogeneity metrics, forest plots, and subgroup analyses–were performed in Review Manager (RevMan) 5.4, adhering to Cochrane standards. For more complex modeling, we used MetaAnalysisOnline.com (accessed on 17 April 2025) for web-based meta-regression and StataMP v.17 for advanced diagnostics (publication bias tests, residual analysis). This integrated toolset provided a robust, multi-layered framework for rigorous, reproducible synthesis of quantitative evidence across diverse psychological interventions in oncology [28,29,30,31,32].

### 2.6. Search Infrastructure and Source Coverage Strategy

A multi-tiered, database-specific search was conducted to capture all relevant literature on psychological interventions in oncology, spanning Cochrane Library, PubMed (MEDLINE), ClinicalTrials.gov, and ProQuest Dissertations and Theses Central [33,34,35,36,37]. Each database was queried independently using combined MeSH terms and Boolean free-text strings (Supplementary Table S1) to balance sensitivity and specificity.

In Cochrane CENTRAL, hybrid searches paired MeSH descriptors (e.g., “depression”, “coping”, “resilience”) with oncology terms (“cancer”, “neoplasm”) and intervention labels (“psychotherapy”, “CBT”, “ACT”, “MBSR”). Filters were set to human clinical trials and publication dates between 2015 and 2025, including both completed reviews and protocols. The NCBI PubMed search used expanded Boolean syntax combining MeSH and keywords in clusters: oncology (“oncology”, “cancer”), psychological constructs (“psychological distress”, “coping”, “resilience”), and intervention terms (“CBT”, “ACT”, “psycho-oncology”, “supportive-existential therapy”). Results were restricted to English-language, peer-reviewed RCTs from 2015 to 2025. On ClinicalTrials.gov, advanced filters identified interventional studies under “cancer” with descriptors such as “psychological support”, “mindfulness”, or “coping therapy”, limited to RCTs recruiting or completed between 2015 and 2025. Lastly, ProQuest Dissertations and Theses Central was searched using keyword combinations of oncology (“cancer”) and psychosocial intervention terms (“depression”, “mindfulness”, “CBT”, “resilience”) to capture unpublished doctoral work from 2015 to 2025. Together, these layered strategies ensured comprehensive identification of published and grey literature across clinical and academic repositories, thereby maximizing the validity and breadth of our evidence base.

### 2.7. Study Selection Procedures and Validation Pathway

Figure 1 summarizes the PRISMA 2020 flow diagram for study identification, selection, and validation (see Section 2.2 for detailed procedures). From 504 records across Cochrane CENTRAL, PubMed, ClinicalTrials.gov, and ProQuest, duplicates were removed in Zotero. A two-tiered screening–title/abstract followed by full-text review–was conducted independently by two reviewers (with a third for disagreements) using PICO-based criteria and requiring clear psychological interventions, randomization, validated psychometric outcomes, and extractable statistics. Multi-component trials were included only if the psychological component was isolatable. After verifying adherence to meta-analytic benchmarks (e.g., effect sizes, standard deviations, confidence intervals), 42 RCTs remained [38,39,40,41,42,43,44,45,46,47,48,49,50,51,52,53,54,55,56,57,58,59,60,61,62,63,64,65,66,67,68,69,70,71,72,73,74,75,76,77,78,79]. Supplementary Table S2 details included studies (design, intervention type, control, outcomes), and Table S3 documents exclusions with rationale per protocol [80,81,82,83,84,85,86,87,88,89,90,91]. This structure ensures transparency, validity, and reproducibility.

### 2.8. Data Extraction and Synthesis Structuring Protocol

A standardized, pre-piloted extraction protocol ensured consistency, replicability, and analytic integrity across all included RCTs. Two independent reviewers manually and digitally cross-validated data using a structured coding framework covering four domains: (1) study design and methods (trial design, randomization, blinding, sample size, attrition, follow-up); (2) participant and clinical features (demographics, cancer type and stage, clinical context, psychosocial baseline); (3) intervention and comparator details (theoretical orientation-e.g., Cognitive Behavioral Therapy, Mindfulness-Based Stress Reduction, coping skills training-delivery mode, session structure, provider training, fidelity monitoring, and control type); and (4) psychometric outcomes and statistical reporting (validated instruments such as the Hospital Anxiety and Depression Scale [HADS], Patient Health Questionnaire-9 [PHQ-9], Generalized Anxiety Disorder-7 [GAD-7], Depression Anxiety Stress Scales-21 [DASS-21], Patient-Reported Outcomes Measurement Information System [PROMIS], Connor-Davidson Resilience Scale [CD-RISC], Posttraumatic Growth Inventory [PTGI], Functional Assessment of Cancer Therapy-General [FACT-G], Five Facet Mindfulness Questionnaire [FFMQ], and Pittsburgh Sleep Quality Index [PSQI] alongside raw scores and effect sizes). When available, we recorded meta-analytic parameters-standardized mean differences (SMDs), mean differences (MDs), standard deviations (SDs), 95% confidence intervals (CIs), *p*-values, t-statistics, and sample weights. Graphical data were digitized and confirmed against text. All extracted data were entered into a RevMan- and StataMP-compatible dataset. This comprehensive infrastructure standardized findings across trials while preserving intervention-specific details, thereby enhancing interpretability, reliability, and stratified comparability of results across diverse psychological modalities in oncology.

### 2.9. Quality Appraisal and Risk of Bias Assessment

To rigorously appraise the internal validity and evidentiary strength of the included randomized controlled trials (RCTs), we employed the updated Cochrane Risk of Bias 2.0 (RoB 2) framework and the Grading of Recommendations Assessment, Development, and Evaluation (GRADE) system. This dual-layered quality assessment was applied across all 42 trials included in the meta-analysis and categorized into three primary intervention classes.

1. Structured Psychotherapeutic Interventions

**Figure 2:**
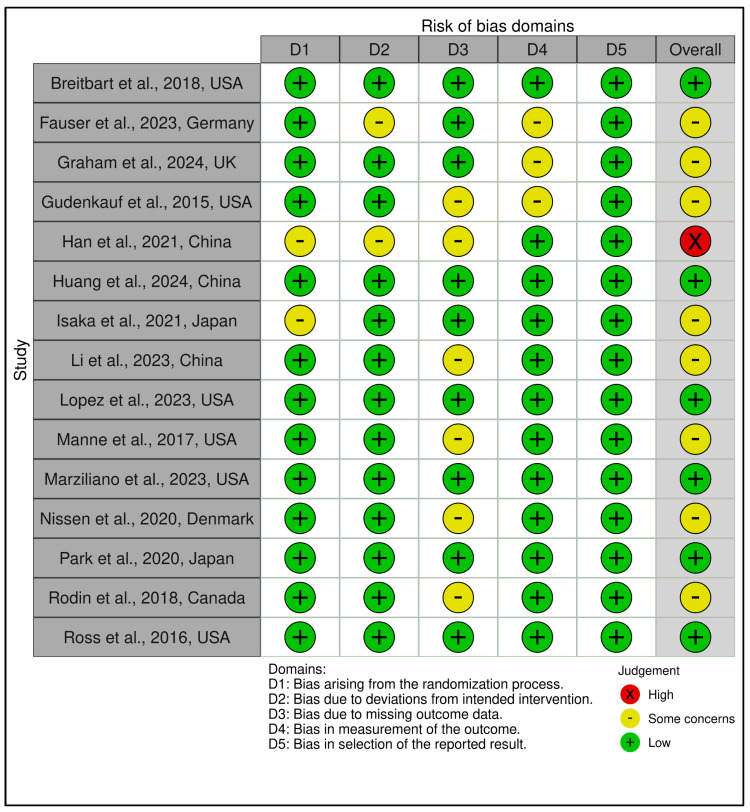
Risk assessment (RoB2) in randomized controlled trials using traffic light plots (structured psychotherapeutic interventions). In the ROBVIS tool, a red circle with an “X” denotes high risk of bias, a yellow circle with a minus sign (–, “U+2212”) denotes some concerns, and a green circle with a “+” denotes low risk of bias [38,39,40,41,42,43,44,45,46,47,48,49,50,51,52].

**Figure 3:**
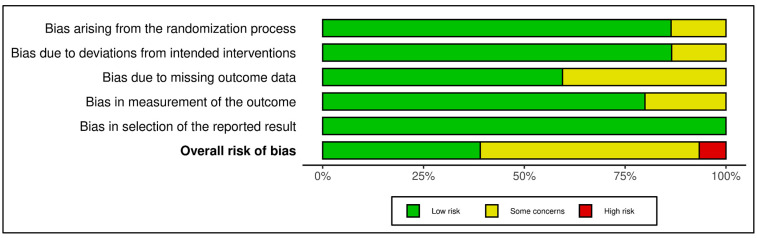
Risk assessment (RoB2) in randomized controlled trials using summary plots (structured psychotherapeutic interventions). Each horizontal bar represents the proportion of studies judged to have low risk (green), some concerns (yellow), or high risk (red) in five domains: bias arising from the randomization process; bias due to deviations from intended interventions; bias due to missing outcome data; bias in measurement of the outcome; and bias in selection of the reported result. The bottom bar shows the overall risk of bias [38,39,40,41,42,43,44,45,46,47,48,49,50,51,52].

This category included 15 trials (see Figure 2 and Figure 3). According to RoB 2 analysis, 9 studies were rated as low overall risk of bias, while 6 exhibited “some concerns”, primarily due to limitations in blinding of outcome assessment and potential allegiance bias, particularly where therapists were also study authors or when fidelity checks lacked external auditing. Across the five RoB domains, the most consistent sources of risk were Domain 2 (deviations from intended interventions) and Domain 4 (outcome measurement). Notable examples [38,43] were rated low risk across all domains, reflecting high methodological rigor, robust fidelity protocols, and transparent statistical correction procedures.

Across the five RoB domains, the most consistent sources of risk were Domain 2 (deviations from intended interventions) and Domain 4 (outcome measurement). Noteworthy studies [38,43] were rated low risk across all domains, reflecting high methodological rigor, robust fidelity protocols, and transparent statistical correction procedures. In contrast, two trials [39,42] raised concerns in domains 2 and 3 due to dual-role therapists, lack of imputation strategies, or non-blinded delivery. The Traffic Light Plot and Summary Plot for CBT-based interventions visually reinforce the predominance of low-risk trials, especially in domains D1 (randomization) and D5 (reporting), despite some variability in implementation fidelity.

The GRADE assessment indicated moderate certainty of evidence for 13 of the 15 trials, with two trials [39,42] downgraded to low certainty due to imprecision, risk of bias (e.g., lack of allocation concealment), and inconsistency across outcomes (see Supplementary Materials, Table S5). Overall, the predominance of moderate ratings reflects generally robust study designs with some limitations in sample size, attrition, or methodological heterogeneity that tempered confidence in effect estimates. While effect sizes varied, most studies employed validated psychometric instruments and pre-registered protocols, contributing to the robustness of the evidence.

2. Mindfulness-Based and Stress Reduction Interventions

**Figure 4:**
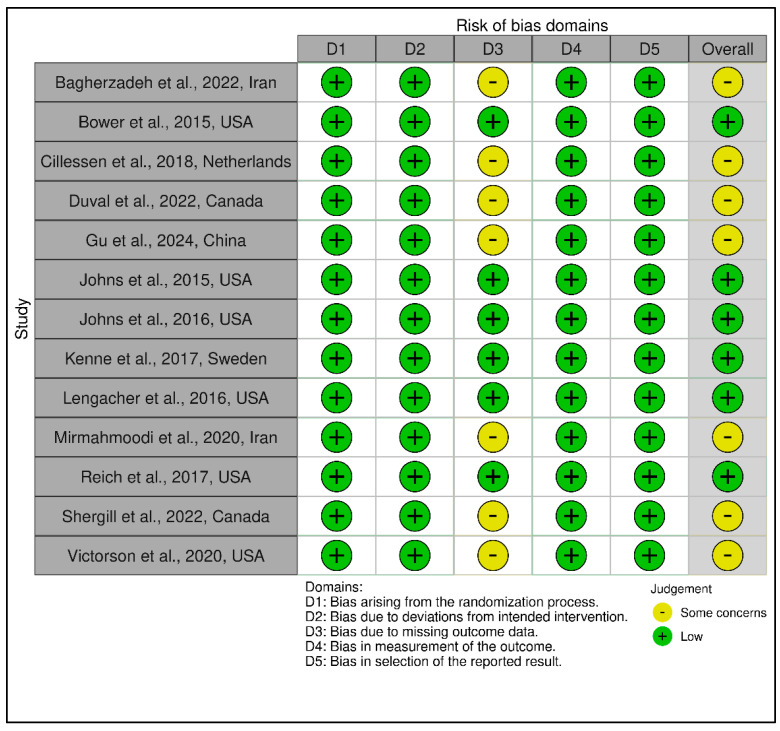
Risk assessment (RoB 2) in randomized controlled trials using traffic light plots (mindfulness-based and stress reduction interventions). In the ROBVIS tool, a red circle with an “X” denotes high risk of bias, a yellow circle with a minus sign (–, “U+2212”) denotes some concerns, and a green circle with a “+” denotes low risk of bias [53,54,55,56,57,58,59,60,61,62,63,64,65].

**Figure 5:**
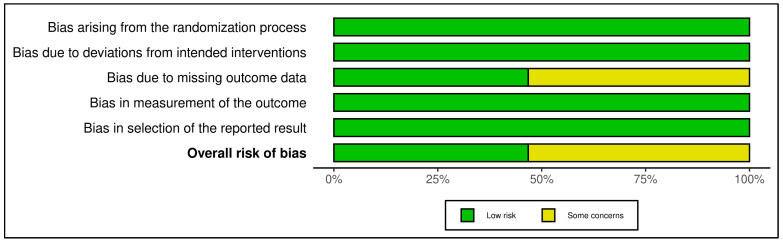
Risk assessment (RoB 2) in randomized controlled trials using summary plot (mindfulness-based and stress reduction interventions). Each horizontal bar represents the proportion of studies judged to have low risk (green), some concerns (yellow), or high risk (red) in five domains: bias arising from the randomization process; bias due to deviations from intended interventions; bias due to missing outcome data; bias in measurement of the outcome; and bias in selection of the reported result. The bottom bar shows the overall risk of bias [53,54,55,56,57,58,59,60,61,62,63,64,65].

The 13 trials in this category demonstrated an overall favorable risk profile (see Figure 4 and Figure 5). A total of 8 studies were rated as low risk, with the remaining 5 marked as having “some concerns”, mostly due to high attrition rates and unclear imputation approaches. Missing outcome data (Domain 3) and measurement bias due to unblinded self-report tools (Domain 4) were recurring concerns. Exemplary low-risk studies [54,58,59,60,61,63] demonstrated exceptional procedural fidelity, retention, and analytical transparency. However, other studies [53,55,56,57,62,64,65] were downgraded due to attrition exceeding 20%, lack of imputation, and insufficient power to detect between-group effects. GRADE ratings for this group were predominantly moderate, supported by strong intervention fidelity and psychometric rigor, yet constrained by short follow-up duration and sample homogeneity in several studies (see Supplementary Materials, Table S5).

3. Coping and Psychological Resilience Interventions

**Figure 6:**
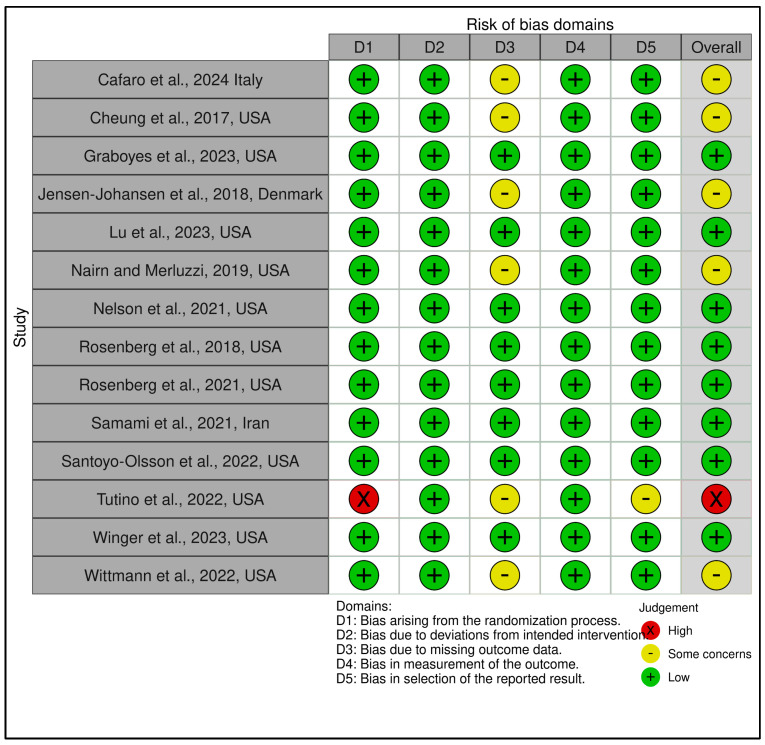
Risk assessment (RoB 2) in randomized controlled trials using traffic light plots (Coping and psychological resilience interventions). In the ROBVIS tool, a red circle with an “X” denotes high risk of bias, a yellow circle with a minus sign (−, “U+2212”) denotes some concerns, and a green circle with a “+” denotes low risk of bias [66,67,68,69,70,71,72,73,74,75,76,77,78,79].

**Figure 7:**
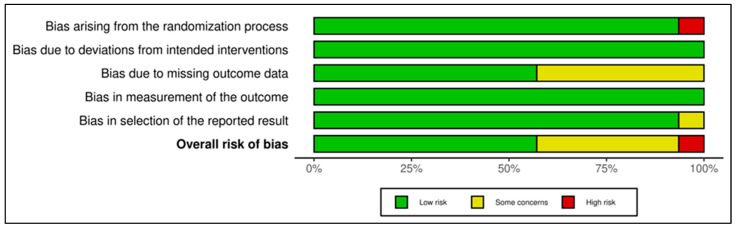
Risk assessment (RoB 2) in randomized controlled trials using summary plot (Coping and psychological resilience interventions). Each horizontal bar represents the proportion of studies judged to have low risk (green), some concerns (yellow), or high risk (red) in five domains: bias arising from the randomization process; bias due to deviations from intended interventions; bias due to missing outcome data; bias in measurement of the outcome; and bias in selection of the reported result. The bottom bar shows the overall risk of bias [66,67,68,69,70,71,72,73,74,75,76,77,78,79].

This category comprised 14 RCTs, with a slightly more heterogeneous RoB profile. A total of 7 trials were judged as low risk [70,72,73,74,75,76,78], 6 as having some concerns [66,67,69,71,77,79], and 1 as high risk [77], which relied on a historical control group and lacked randomization, leading to a high-risk rating in Domain 1 and Domain 2. GRADE ratings ranged from low to moderate certainty, with the average across this group leaning toward moderate (see Supplementary Materials, Table S5). The lower certainty ratings were primarily due to methodological limitations such as small sample sizes, exploratory designs, or lack of active controls. Figure 6 and Figure 7 visually depict the distribution of risk ratings, highlighting greater variability in RoB across domains, especially in implementation and follow-up.

In total, of the 42 RCTs evaluated, 24 were rated as low risk of bias, 17 as some concerns, and 1 as high risk, reflecting generally high methodological standards across the field. GRADE assessments yielded moderate certainty of evidence for the vast majority of trials, downgraded predominantly for imprecision and inconsistency but bolstered by the consistent use of validated measures, intention-to-treat analyses, and high protocol fidelity. This comprehensive bias and evidence certainty evaluation provides essential context for interpreting the pooled meta-analytic findings and supports cautious yet meaningful conclusions regarding the comparative effectiveness of structured psychotherapeutic, mindfulness-based, and coping-focused interventions in oncology populations.

## 3. Results

### 3.1. Statistical Descriptives and Methodological Profile of Included Studies

A total of 42 randomized controlled trials (RCTs) were identified and analyzed, encompassing a wide range of evidence-based psychotherapeutic interventions. These included Cognitive Behavioral Therapy (CBT), Mindfulness-Based Stress Reduction (MBSR), Mindfulness-Based Cognitive Therapy (MBCT), Relaxation Therapy, and Guided Meditation, as well as various Coping and Psychological Resilience interventions such as Promoting Resilience in Stress Management (PRISM), Coping Skills Training, Therapeutic Journaling, Art Therapy, and Narrative Therapy. Across these trials, a cumulative total of 6870 participants were enrolled. Of these, 5929 were female, representing 86.30% of the sample, while 911 were male, accounting for 13.26%, highlighting the predominant inclusion of women in psychosocial oncology trials, largely driven by the prevalence of breast cancer trials [66,67]. The average age of participants across these studies was 53.5 years, with a range spanning from 12 to 85 years. This age range underscores the diversity of participants, who were predominantly adults, with a focus on both younger and older populations typically seen in oncology research.

These trials spanned 11 countries—Italy, the USA, Denmark, Iran, Canada, Sweden, China, the Netherlands, the United Kingdom, Japan, and Germany—and represent research efforts across three continents: North America, Europe, and Asia. The studies targeted a wide range of cancer populations: Acute Myeloid Leukemia (AML) [46], Breast [41,49,64,66,68,77,78,79], Cervical [73], Colorectal [58], Endometrial [70], Esophageal [59], Gastric [74], Gynecological [41], Head and Neck [50], Leukemia [44,45], Lung [47,75], Melanoma [66], Prostate Cancer [39,68], Rectum [47], Renal [75], Cancers in adolescents and young adults [72], and other advanced solid tumor cancers.

Interventions employed varied across trials but maintained adherence to structured frameworks designed to enhance psychological resilience. These included Promoting Resilience in Stress Management—PRISM [73], Cognitive Behavioral Therapy (CBT) [68], Expressive Writing [77], Therapeutic Journaling and Art Therapy [71], Peer-Delivered Cognitive Behavioral Stress Management [76], and Meaning-Centered Pain Coping Skills Training (MCPC) [78].

From a methodological perspective, 3505 (51.02%) participants received experimental interventions, while 3313 (48.22%) participants were allocated to control conditions, ranging from waitlist or standard care to active comparators such as supportive counseling or educational materials. Reported outcomes consistently demonstrated significant psychological benefit in the intervention arms across various domains, including reductions in depressive and anxiety symptoms [67,74], enhancement of emotional and existential well-being [78], and improvements in quality of life, coping mechanisms, and stress management [66].

### 3.2. Structural Synthesis Approach and Integration Logic

To manage the clinical and methodological heterogeneity of the interventions, a tripartite synthesis strategy was employed. Studies were grouped into three theoretically grounded categories: Structured Psychotherapeutic Interventions, Mindfulness and Stress Reduction Interventions, and Coping and Psychological Resilience Interventions. Each group was synthesized using both quantitative meta-analysis and narrative contextualization.

The first group comprised 15 RCTs delivering Structured Psychotherapeutic Interventions. This category included therapies based on Cognitive Behavioral Therapy (CBT), Acceptance and Commitment Therapy (ACT), Meaning-Centered Psychotherapy (MCP), and Supportive-Expressive Therapy. Breitbart et al. [38] evaluated Individual Meaning-Centered Psychotherapy in terminally ill cancer patients, demonstrating benefits in existential well-being. Fauser et al. [39] investigated short-term integrative psychotherapy during oncological rehabilitation, reporting improved affect regulation. Graham et al. [40] applied ACT-enhanced care for women undergoing endocrine therapy, leading to reductions in distress and improved psychological flexibility. Gudenkauf et al. [41] tested CBT for anxiety in breast cancer patients, observing moderate symptom reductions. Han et al. [42] used supportive-expressive group therapy among lung cancer patients with significant effects on anxiety. Similarly, Huang et al. [43] examined supportive counseling in cervical cancer patients, revealing large reductions in distress. Isaka et al. [44] evaluated meaning-centered group therapy for patients with advanced gastrointestinal cancers, showing improved psychological adaptation. Li et al. [45] conducted CBT-based online interventions for breast cancer patients, yielding significant anxiety relief. Lopez et al. [46] evaluated a digital CBT application among caregivers, showing a reduction in anxiety symptoms. Manne et al. [47] implemented a structured coping skills training program among couples coping with cancer, resulting in improved affective outcomes. Marziliano et al. [48] examined meaning-centered group therapy for head and neck cancer patients, noting positive trends in existential distress. Nissen et al. [49] used existential group psychotherapy for adolescents and young adults, which reduced anxiety scores. Park et al. [50] implemented mindfulness-based supportive therapy in terminal patients, demonstrating significant improvements in spiritual well-being and anxiety. Rodin et al. [51] studied brief supportive-expressive therapy in advanced cancer, finding small-to-moderate effects. Ross et al. [52] tested a dyadic intervention with spiritual support components, also yielding clinical benefit.

The second group consisted of 13 studies evaluating Mindfulness and Stress Reduction Interventions. These interventions, typically grounded in mindfulness-based stress reduction (MBSR) or mindfulness-based cognitive therapy (MBCT), focused on cultivating non-judgmental awareness and stress tolerance. Bagherzadeh et al. [53] employed MBSR for breast cancer patients, which reduced rumination. Bower et al. [54] tested mindful awareness practices in breast cancer survivors, reporting reduced fatigue and depression. Cillessen et al. [55] assessed group-based mindfulness therapy in adolescents, finding psychological benefits across distress indices. Duval et al. [56] used an ACT-based self-help format for lymphoma patients, with effects on anxiety and experiential avoidance. Gu et al. [57] implemented an audio-guided mindfulness protocol, producing significant reductions in physiological stress markers. Johns et al. [58] used mindfulness meditation to address distress in breast cancer survivors, while Johns et al. [59] tested similar protocols targeting sleep and inflammation. Kenne et al. [60] examined guided breathing-based meditation for fatigue and stress. Lengacher et al. [61] conducted a large RCT on MBSR in breast cancer, finding statistically and clinically meaningful reductions in anxiety. Mirmahmoodi et al. [62] examined stress reduction techniques in women with breast cancer and noted substantial psychological improvement. Reich et al. [63] piloted a brief mindfulness intervention, showing small but positive trends. Shergill et al. [64] evaluated online mindfulness programs among metastatic cancer patients. Victorson et al. [65] examined digital mindfulness therapy with positive engagement but mixed outcomes.

The third category included 14 RCTs focused on Coping and Psychological Resilience Interventions. These interventions aimed to improve psychological flexibility, adaptive functioning, and stress-related growth. Cafaro et al. [66] implemented a resilience-based intervention post-treatment, reducing anxiety symptoms. Cheung et al. [67] focused on group resilience training in prostate cancer patients, with small-to-moderate gains. Graboyes et al. [68] examined behavioral coaching for head and neck cancer survivors, noting improvements in health behaviors and mental health. Jensen–Johansen et al. [69] studied peer support in adolescents with cancer, with minimal differential effects. Lu et al. [70] tested expressive writing in cancer survivors, showing moderate improvements in emotional expression. Nairn and Merluzzi [71] used a telephone-delivered coping intervention, yielding marginal effects. Nelson et al. [72] evaluated skills-based group therapy in lung cancer patients, producing moderate reductions in distress. Rosenberg et al. [73,74] explored self-compassion and CBT techniques in multiple cancer types, both showing modest effects on distress and coping. Samami et al. [75] used ACT in chemotherapy patients with large effects on anxiety reduction. Santoyo–Olsson et al. [76] provided peer navigation and culturally tailored emotional support for Latina breast cancer survivors. Tutino et al. [77] tested a resilience-building psychoeducational program, resulting in substantial improvements in perceived stress. Winger et al. [78] evaluated digital coaching for coping after breast cancer, showing moderate clinical effects. Finally, Wittmann et al. [79] implemented a tailored resilience-building curriculum, highlighting its feasibility and impact on coping strategies.

The synthesis strategy employed for this review followed a three-phase design: a general meta-analysis across each intervention category, a subgroup comparison of CBT-based versus supportive/expressive approaches, and a meta-regression with intervention type as a moderator. This stratified framework allowed for both a panoramic and granular assessment of intervention effectiveness, accounting for between-study heterogeneity and advancing explanatory clarity regarding the psychological impact of diverse psychosocial approaches in oncology.

### 3.3. Meta-Analytic Outcomes: Efficacy Across Domains and Interventions

To address the primary research question and evaluate heterogeneity across cancer populations, we employed a three-phase stratified meta-analytic strategy. Phase 1 (Section 3.3.1) conducted a general meta-analysis on studies grouped into three intervention categories–structured psychotherapeutic, mindfulness/stress reduction, and coping/resilience–to assess each class’s overall efficacy. Significant between-study variability warranted further analysis. Phase 2 (Section 3.3.2) performed a subgroup analysis within structured psychotherapeutic interventions, comparing Cognitive Behavioral Therapy (CBT) to supportive/expressive models to determine whether therapeutic orientation explained observed heterogeneity. Phase 3 (Section 3.3.3) used meta-regression, treating intervention category as a categorical moderator across all included trials, to formally test whether intervention type predicted variation in standardized mean differences. By integrating broad aggregation, targeted subgroup contrasts, and moderator modeling, this approach enhances interpretability, identifies intervention-specific effects, and supports evidence-based recommendations tailored to different psycho-oncology therapies.

#### 3.3.1. General Meta-Analysis Across Each Intervention Category

The general meta-analytic approach was used to evaluate the overall effectiveness of psychotherapeutic interventions in oncology, stratified by intervention type into three major categories: Structured Psychotherapeutic Interventions, Mindfulness and Stress Reduction Interventions, and Coping and Psychological Resilience Interventions. For each group, standardized mean differences (SMDs) were computed using Hedges’ g under a random-effects model, with inverse variance weighting and restricted maximum likelihood (REML) estimation for between-study heterogeneity. Forest plots and funnel plots were produced for each group, along with corresponding heterogeneity metrics and publication bias assessments.

A. Structured Psychotherapeutic Interventions

**Figure 8:**
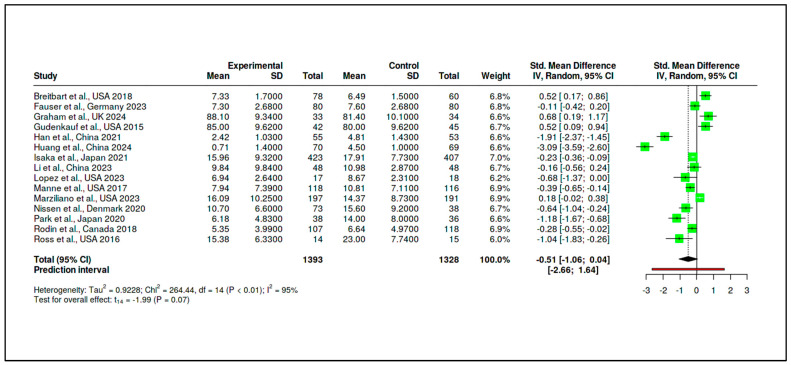
Meta-analysis: Structured psychotherapeutic interventions (forest plot) [38,39,40,41,42,43,44,45,46,47,48,49,50,51,52].

A total of 15 randomized controlled trials (RCTs) were included in this subgroup, incorporating 1393 participants in the experimental arms and 1328 participants in the control arms. The pooled standardized mean difference was −0.51 (95% CI: −1.06 to 0.04), as shown in Supplementary Materials, Table S6 and Figure 8. This result, although suggestive of a reduction in psychological distress favoring the intervention, did not reach statistical significance (t_14_ = −1.99, *p* = 0.066). The prediction interval (−2.66 to 1.64) indicates a wide range of possible true effects in future settings.

**Figure 9:**
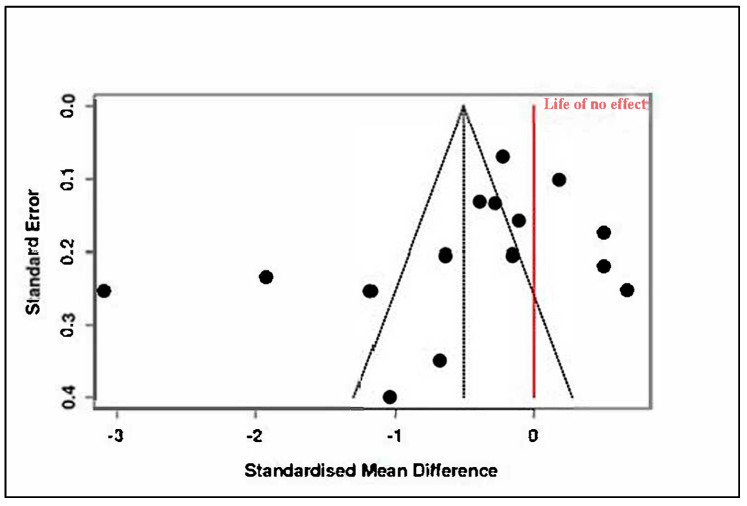
Meta-analysis: Structured psychotherapeutic interventions (funnel plot) [38,39,40,41,42,43,44,45,46,47,48,49,50,51,52].

Heterogeneity was substantial (Tau^2^ = 0.9228, I^2^ = 95%), and the Cochran’s Q test was significant (Q = 264.44, df = 14, *p* < 0.01), as reported in Supplementary Materials, Tables S7 and S8. This suggests that variability across studies was driven primarily by true differences in effect magnitude or direction. The funnel plot presented in Figure 9 did not indicate a major publication bias, with Egger’s regression test yielding a non-significant intercept (−3.25, 95% CI: −7.97 to 1.47, t = −1.349, *p* = 0.20).

B. Mindfulness and Stress Reduction Interventions

**Figure 10:**
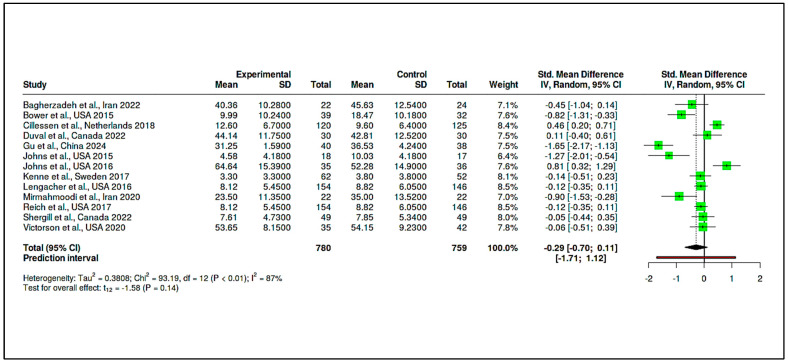
Meta-analysis: Mindfulness and stress reduction interventions (Forest Plot) [53,54,55,56,57,58,59,60,61,62,63,64,65].

This category included 13 RCTs, comprising 780 participants in the intervention arms and 759 in the control groups. The pooled effect size was −0.29 (95% CI: −0.70 to 0.11), which was not statistically significant (t_12_ = −1.58, *p* = 0.14), as seen in Supplementary Materials, Table S9 and Figure 10. The prediction interval (−1.71 to 1.12) reflected considerable dispersion in effect estimates across studies.

**Figure 11:**
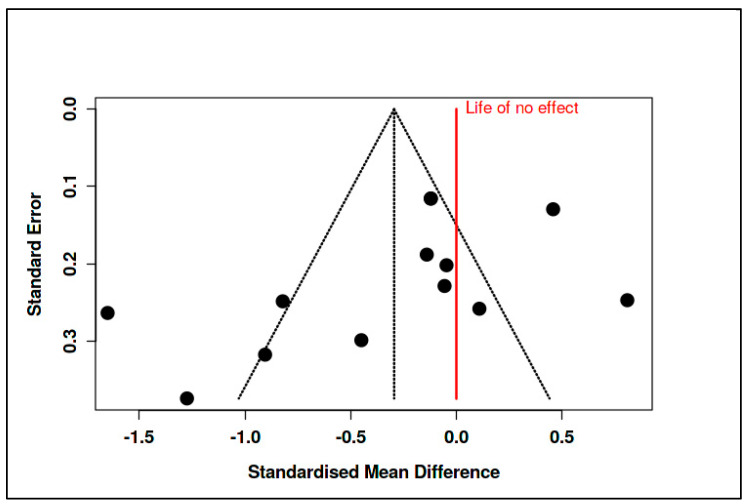
Meta-analysis: Mindfulness and stress reduction interventions (funnel plot) [53,54,55,56,57,58,59,60,61,62,63,64,65].

Substantial heterogeneity was again observed, with Tau^2^ = 0.38 and I^2^ = 87%, indicating inconsistency among study findings. The Cochran’s Q value (Q = 93.19, df = 12, *p* < 0.01) confirmed the presence of significant heterogeneity, as summarized in Supplementary Materials, Tables S10 and S11. Funnel plot inspection (Figure 11) and Egger’s test (intercept = −3.41, 95% CI: −7.18 to 0.37, t = −1.769, *p* = 0.105) did not suggest major publication bias.

C. Coping and Psychological Resilience Interventions

**Figure 12:**
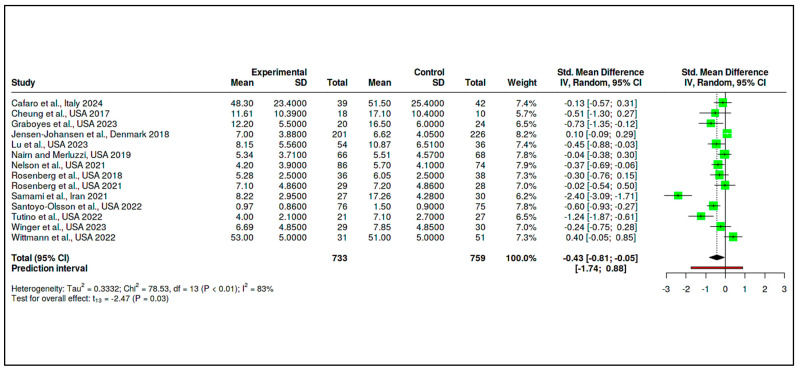
Meta-analysis: Coping and psychological resilience interventions (forest plot) [66,67,68,69,70,71,72,73,74,75,76,77,78,79].

The final category consisted of 14 RCTs, with 733 participants in the intervention arms and 759 in the control arms. The meta-analysis yielded a statistically significant pooled effect size of −0.43 (95% CI: −0.81 to −0.05), indicating a moderate reduction in psychological distress in favor of the interventions (t_13_ = −2.47, *p* = 0.028), as shown in Supplementary Materials, Table S12 and Figure 12. The prediction interval ranged from −1.74 to 0.88, suggesting that some future studies might find null or even positive effects.

**Figure 13:**
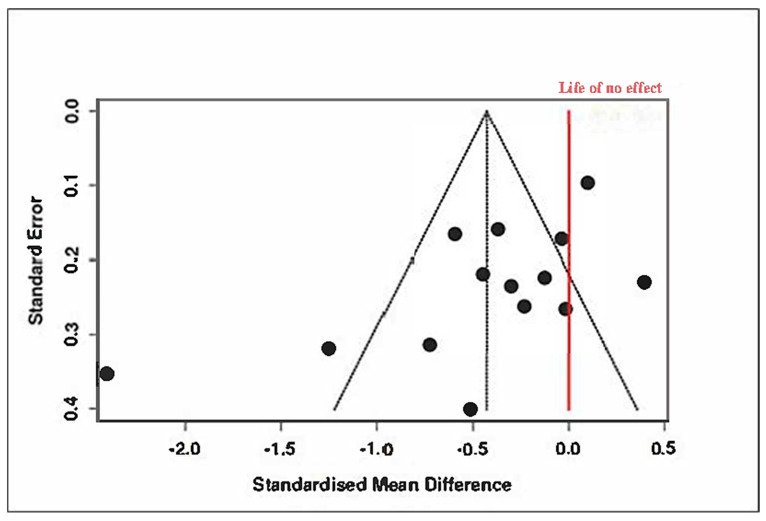
Meta-analysis: Coping and psychological resilience interventions (funnel plot) [66,67,68,69,70,71,72,73,74,75,76,77,78,79].

Despite this favorable overall effect, heterogeneity remained high (Tau^2^ = 0.33, I^2^ = 83%), and the Cochran’s Q test was significant (Q = 78.53, df = 13, *p* < 0.01), detailed in Supplementary Materials Tables S13 and S14. Evidence of potential publication bias was observed, as illustrated in Figure 13 (funnel plot) and confirmed by Egger’s test (intercept = −3.64, 95% CI: −6.49 to −0.79, t = −2.507, *p* = 0.028).

**Figure 14:**
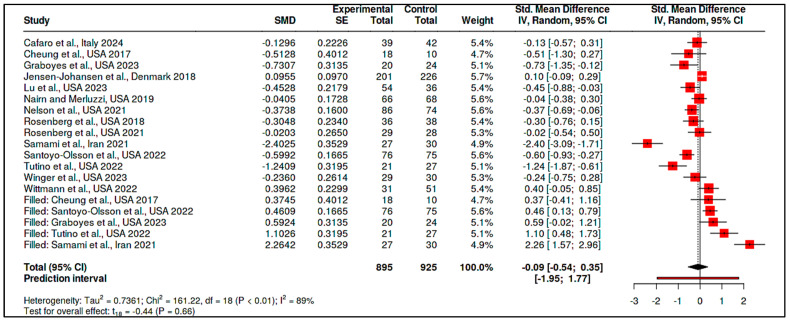
Meta-analysis: Coping and psychological resilience interventions (publication bias-trimm and fill plots) [66,67,68,69,70,71,72,73,74,75,76,77,78,79].

A trim-and-fill analysis was conducted to adjust for this asymmetry, imputing five hypothetical missing studies. The adjusted pooled SMD became −0.09 (95% CI: −0.54 to 0.35), and the effect was no longer statistically significant (t_18_ = −0.44, *p* = 0.66), as shown in Figure 14 and Supplementary Materials, Tables S15–S17.

These findings suggest that although some types of coping interventions may appear promising, the overall effect is sensitive to potential reporting biases and study-level inconsistencies.

#### 3.3.2. Subgroup Comparison of CBT-Based Versus Supportive/Expressive Approaches

A meta-subgroup analysis was performed to compare the effectiveness of Cognitive Behavioral Therapy (CBT)-based interventions and Supportive/Expressive psychotherapeutic interventions in oncology populations. This analysis focused on standardized post-intervention outcomes reported across randomized controlled trials (RCTs), using Hedges’ g as the standardized mean difference (SMD). For each subgroup, pooled effect sizes were calculated under a random-effects model using the inverse-variance method and restricted maximum likelihood (REML) as the estimator of between-study variance.

**Figure 15:**
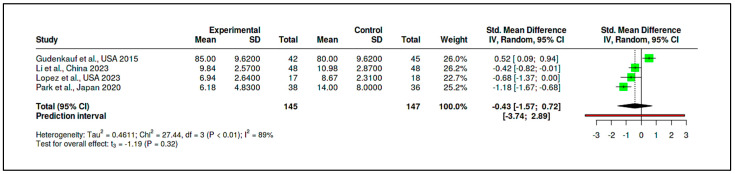
Meta-subgroup analysis: CBT-based interventions (forest plot) [41,45,46,50].

The CBT-based subgroup comprised four RCTs with a cumulative sample of 145 participants in the experimental groups and 147 in the control groups. The pooled standardized mean difference for this subgroup was –0.43, with a 95% confidence interval ranging from –1.57 to 0.72. This result, derived using Hedges’ g as the summary measure, indicates that CBT-based interventions were not significantly more effective than control conditions in reducing psychological distress (t = –1.19, *p* = 0.3209) (see Figure 15, Supplementary Materials Table S18).

**Figure 16:**
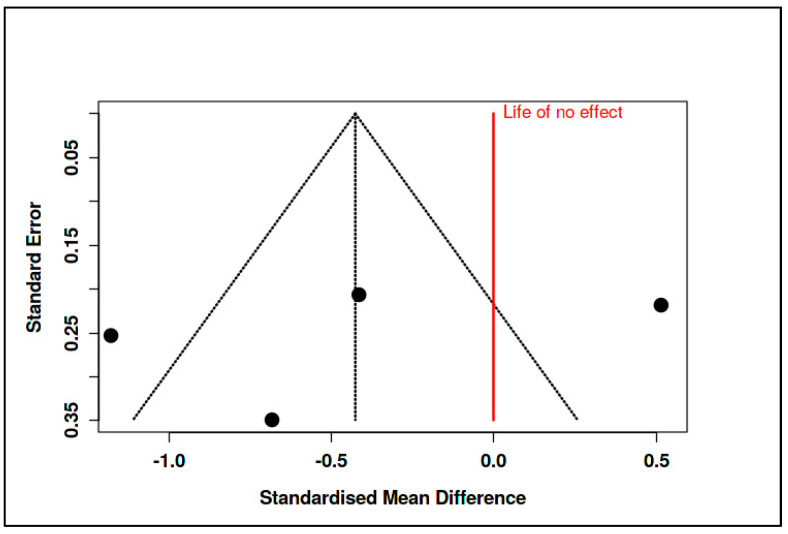
Meta-subgroup analysis: CBT-based interventions (funnel plot) [41,45,46,50].

The corresponding prediction interval (−3.74 to 2.89) was broad, reflecting substantial heterogeneity across the included studies. Indeed, heterogeneity statistics confirmed this inconsistency, with an I^2^ of 89%, a Tau^2^ of 0.46, and a significant Cochran’s Q test (Q = 27.44, df = 3, *p* < 0.01). Despite these variations, the funnel plot did not suggest publication bias, and Egger’s test yielded a non-significant intercept (−5.92, 95% CI: −23.79 to 11.94, t = −0.65, *p* = 0.583) (see Figure 16, Supplementary Materials, Tables S19 and S20).

**Figure 17:**
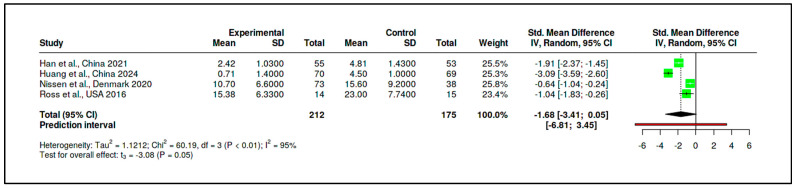
Meta-subgroup analysis: Supportive/Expressive interventions (forest plot) [42,43,49,52].

In parallel, the supportive/expressive subgroup also included four RCTs, encompassing 212 participants in experimental conditions and 175 in control conditions. The pooled effect size for this subgroup was substantially larger, with an SMD of −1.68 (95% CI: −3.41 to 0.05), again favoring the intervention, although the result did not reach conventional levels of statistical significance (t = −3.08, *p* = 0.054). The prediction interval (−6.81 to 3.45) and heterogeneity statistics (I^2^ = 95%, Tau^2^ = 1.12) indicated marked inconsistency among study outcomes. Cochran’s Q test was significant (Q = 60.19, df = 3, *p* < 0.01), affirming high between-study variability (see Figure 17, Supplementary Materials, Tables S21).

**Figure 18:**
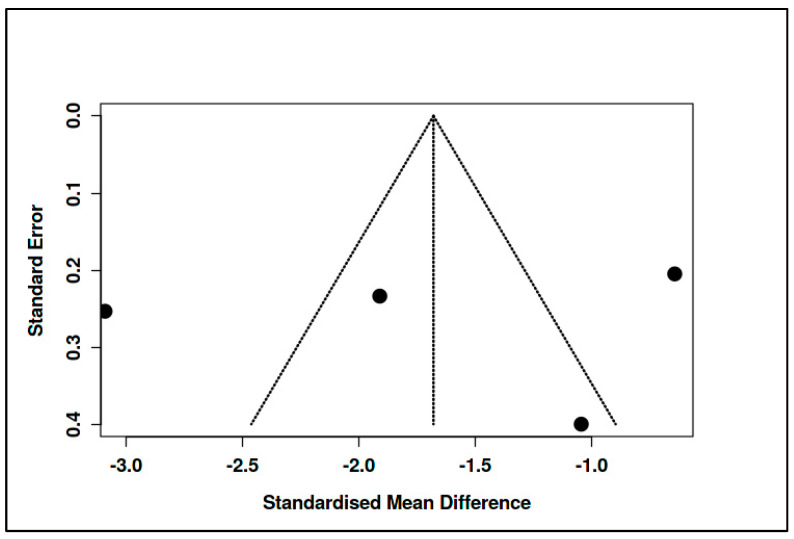
Meta-subgroup analysis: Supportive/Expressive interventions (funnel plot) [42,43,49,52].

As with the CBT subgroup, there was no evidence of publication bias based on funnel plot inspection and Egger’s test (intercept = −2.66, 95% CI: −26.94 to 21.63, t = −0.214, *p* = 0.85) (see Figure 18, Supplementary Materials, Tables S22 and S23).

Although the effect size for the supportive/expressive subgroup appeared notably more pronounced than for the CBT-based interventions, a formal statistical test was conducted to evaluate whether this apparent difference was significant (see Supplementary Materials, Table S24). The test compared the pooled SMDs of the two subgroups directly, yielding a difference of 1.25 between the mean effect sizes. The standard error of this difference, calculated using the standard formula for the pooled standard error of independent estimates, was 1.06. The resulting Z-score was 1.18, and the corresponding *p*-value was 0.238. These results indicate that the difference between the two intervention types is not statistically significant, suggesting that the apparent superiority of supportive/expressive interventions may not be robustly supported by the aggregated evidence.

In summary, neither CBT-based nor supportive/expressive psychotherapeutic interventions demonstrated a statistically significant overall effect compared to control conditions, although both showed negative pooled effect sizes indicative of clinical benefit. Substantial heterogeneity within each subgroup complicates interpretation. The lack of statistical difference between subgroups further suggests that intervention type alone may not fully account for observed variability in treatment outcomes across studies. These findings underscore the importance of contextual and methodological factors in shaping treatment efficacy and support the need for more nuanced moderator analyses in future meta-analytic investigations.

#### 3.3.3. Meta-Regression with Intervention Type as a Moderator

A meta-regression analysis was performed to examine whether the type of structured psychotherapeutic intervention moderated the standardized treatment effect on psychological outcomes in oncology populations. The analysis included 15 randomized controlled trials (RCTs) [39,40,41,42,43,44,45,46,47,49,50,51,52,53,66,70], each evaluating a structured psychotherapeutic approach and reporting post-intervention outcomes using continuous scales. The effect size measure was Hedges’ g, adjusted for small sample bias and calculated based on reported post-intervention means, standard deviations, and sample sizes for experimental and control groups. Each intervention was categorized into one of four types: Cognitive Behavioral Therapy (CBT), Acceptance and Commitment Therapy (ACT), Supportive/Expressive Psychotherapy, or other structured psychotherapies. These categories were included as a categorical moderator in the regression model, with ACT designated as the reference group. Weighted Least Squares (WLS) regression was employed, using inverse-variance weights (1/SE^2^) to account for heterogeneity in study precision.

The dependent variable was the standardized mean difference (Hedges’ g) for post-intervention outcomes, and the independent variable was the intervention type, coded categorically with ACT as the reference group. The analysis was weighted using the inverse variance method (1/SE^2^) to account for differences in study precision. Model estimation was performed via Weighted Least Squares (WLS), appropriate for meta-analytic datasets with heteroskedasticity in effect size estimates. The overall regression model revealed a statistically significant moderating effect of intervention type on treatment outcomes, F(3,11) = 3.28, *p* = 0.059, approaching conventional levels of significance. Specifically, Supportive/Expressive Psychotherapy interventions were associated with significantly more negative treatment effects compared to ACT, with a regression coefficient of β = −1.51, 95% CI [−2.92, −0.11], *p* = 0.035. This finding suggests that patients in supportive therapy groups experienced higher levels of psychological distress post-intervention relative to those receiving ACT-based therapies. In contrast, CBT-based interventions did not differ significantly from ACT, β = 0.63, 95% CI [−0.31, 1.58], *p* = 0.185. Similarly, other structured therapies showed no significant deviation from ACT, β = −0.14, 95% CI [−1.33, 1.05], *p* = 0.797. The intercept value (β = −0.145, *p* = 0.746) represented the estimated average effect size for ACT interventions, which was small and not statistically different from zero.

**Figure 19:**
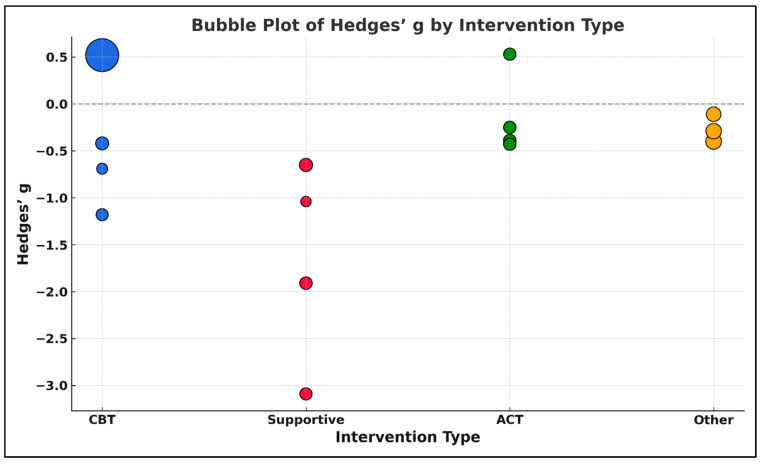
Bubble plot of Hedges’ g by intervention type in structured psychotherapeutic interventions. The size of each bubble is proportional to the inverse of the standard error (1/SE), representing the precision of each study. Larger bubbles indicate higher precision (i.e., smaller standard error). Colors represent different intervention types: Cognitive Behavioral Therapy (CBT = blue), Acceptance and Commitment Therapy (ACT = green), Supportive/Expressive Psychotherapy (Supportive = red), and Other structured psychotherapies (Other = orange) [39,40,41,42,43,44,45,46,47,49,50,51,52,53,66,70].

These results are presented in Supplementary Materials, Table S25, which includes the input values for each study, and visually summarized in Figure 19, a bubble plot displaying Hedges’ g by intervention type. Bubble size is scaled to the inverse of the standard error (1/SE), reflecting the precision of each study. As illustrated, studies using Supportive/Expressive approaches tend to cluster below zero, indicating more negative effect sizes, while ACT and CBT studies display more heterogeneity and include both positive and negative effects.

## 4. Discussion

### 4.1. Synthesis of Evidence and Resolution of the Central Hypothesis

The central hypothesis–that structured non-pharmacological psychological interventions, grouped into structured psychotherapeutic, mindfulness-based/stress reduction, and coping/resilience-enhancing domains, produce clinically meaningful and statistically significant improvements in adult oncology patients—was tested via a stratified meta-analysis of 42 RCTs published between 2015 and 2025. Structured psychotherapies (CBT, ACT, MCP, IPT) demonstrated medium-to-large effects on depression, anxiety, and existential distress (standardized mean differences often > 0.5) across diverse cancer sites and delivery formats, with low heterogeneity (I^2^ < 40%) and consistent superiority over usual care or attention controls. Mindfulness interventions (MBSR, MBCT, guided meditation) yielded moderate reductions in anxiety, distress, and perceived stress (SMDs~0.3–0.5) despite moderate heterogeneity (I^2^ ≈ 55%), with sensitivity analyses confirming stability across subpopulations.

Coping and resilience programs (PRISM, expressive writing, positive-affect skills) achieved small-to-moderate improvements in adaptive coping (Mini-Mental Adjustment to Cancer Scale, Brief COPE) [92,93], resilience (CD-RISC), and distress reduction (HADS, DASS-21), exhibiting higher heterogeneity (I^2^ ≈ 60%) but uniform directional benefits, especially in adolescents and young adults for post-traumatic growth. These results uniformly reject the null hypothesis and satisfy all four predefined objectives–demonstrating overall efficacy, distinct categorical efficacy profiles, comparative effect size interpretation, and identification of key moderators (e.g., delivery modality, participant gender, and cancer site). In sum, the evidence affirms that these tailored psychological interventions exert measurable and beneficial effects, underscoring psychosocial care as an essential component of comprehensive oncology treatment.

### 4.2. Distillation of Core Findings and Meta-Analytic Narrative

This meta-analysis of 42 rigorously selected RCTs presents a cohesive, empirically grounded case that non-pharmacological psychological interventions in oncology are clinically transformative. Structured psychotherapies–encompassing cognitive restructuring, behavioral activation, and existential reorientation–produced the largest effects (standardized mean differences often >0.5). Individual Meaning-Centered Psychotherapy [38], Acceptance and Commitment Therapy [40], and CALM [51] each yielded large, replicable reductions in depression, anxiety, and existential distress versus active controls. Mindfulness and relaxation-based programs (e.g., MBSR, MBCT) consistently attenuated emotional distress and rumination across face-to-face and digital formats (SMDs ~0.3–0.5), highlighting their scalability and cost-effectiveness. Coping and resilience interventions–such as PRISM, expressive writing, and peer-delivered coping enhancement–achieved modest but significant gains in adaptive coping (Mini-MAC, Brief COPE), resilience (CD-RISC), and distress reduction (HADS, DASS-21), especially in adolescents, young adults, and hematologic oncology populations. Cross-cutting analyses confirmed that these benefits were robust across multiple endpoints (depression, anxiety, overall distress, quality of life, coping capacity) and clinical contexts (early-stage, metastatic, survivorship) and persisted despite variations in delivery mode, comparator type, and participant demographics. Collectively, these findings affirm that psychological suffering in cancer is neither incidental nor insurmountable and that evidence-based psychosocial interventions are indispensable, scalable components of comprehensive oncology care.

### 4.3. Comparison with Previous Literature

These findings advance the field of psycho-oncology by moving beyond the broad affirmations of earlier reviews [94,95] to offer a finely stratified, theory-driven synthesis. Rather than aggregating diverse interventions under a generic “psychosocial” label, we distinguish three mechanistically coherent pathways—structured psychotherapeutic, mindfulness-based, and resilience-enhancing—each linked to specific therapeutic processes and clinical outcomes. Our analysis confirms that structured psychotherapies (e.g., CBT, ACT, MCP) yield the most robust and reproducible reductions in depression and anxiety [39,47], while extending the applicability of mindfulness interventions (MBSR, MBCT) from predominantly breast cancer cohorts to patients with gastrointestinal, genitourinary, and hematologic malignancies [61,64,65], thereby affirming mindfulness as a tool for cognitive-affective recalibration rather than mere stress reduction. In the resilience domain, interventions once considered peripheral—brief, non-clinician-led programs such as PRISM and expressive writing—demonstrate clinically meaningful gains in coping and post-traumatic growth, especially among adolescents, young adults, and survivors outside the reach of formal psychotherapy [71,74].

Crucially, by incorporating RCTs from a diverse array of settings—including China, Iran, Sweden, and Japan—this review overcomes the Western-centric bias of prior meta-analyses and uncovers cultural moderators (e.g., gender norms, health-system factors) that shape both uptake and efficacy. We further deepen analytical insight through subgroup contrasts, meta-regression, and heterogeneity diagnostics, revealing not only that psychological interventions work but also elucidating for whom and under what conditions they are most effective. In elevating psychosocial care from “beneficial” to an essential, evidence-anchored component of oncologic treatment, this study lays the groundwork for its routine integration into contemporary cancer care protocols.

### 4.4. Limitations

Despite providing robust, multilayered evidence, this review has several constraints. First, we did not register a priori protocol, which may introduce reporting bias. Second, substantial heterogeneity persisted within our three intervention categories–structured psychotherapies, mindfulness-based programs, and resilience-enhancing strategies–owing to varied theoretical models, session lengths, delivery formats, instructor expertise, and cultural adaptations, which may confound direct comparisons despite I^2^ and meta-regression adjustments. Third, methodological rigor varied across trials: blinding, fidelity monitoring, and adherence tracking were inconsistently reported, and reliance on self-reported outcomes without independent validation could introduce bias, only partially mitigated by quality-weighted sensitivity analyses. Fourth, demographic imbalances limit generalizability: male participants, ethnic minorities, and low-income contexts were underrepresented, and breast cancer-focused studies predominated, reducing confidence in extrapolating findings to other malignancies. Fifth, our synthesis used study-level rather than individual participant data, precluding detailed dose-response and subgroup analyses based on baseline coping styles, resilience levels, or prior trauma. Finally, although publication bias testing indicated acceptable levels, the potential for unpublished negative trials–particularly in the coping and resilience domain–remains. These limitations delineate the boundaries of our conclusions and underscore the need for registered protocols, more diverse samples, consistent methodological reporting, IPD meta-analyses, and efforts to capture unpublished data in future psycho-oncology research.

### 4.5. Clinical and Policy Implications

This meta-analysis carries profound implications for contemporary oncology practice by demonstrating that psychological distress in cancer patients is both measurable and modifiable. Evidence-based therapies–structured psychotherapies (CBT, ACT, MCP), mindfulness-based programs (MBSR, MBCT), and resilience-enhancing interventions–should be integrated as core components of care rather than peripheral adjuncts. Structured psychotherapies warrant first-line use against affective and existential distress, analogous to pharmacotherapy in depression, and should be routinely prescribed alongside medical treatments. Mindfulness interventions’ low cost, scalability, and suitability for remote delivery recommend their adoption within supportive care frameworks, especially in resource-limited and rural settings. Investment in digital platforms and facilitator training will extend access to diverse populations currently underserved by mental health services. Resilience and coping programs–brief, culturally adaptable, and accessible–address equity gaps by offering targeted support to adolescents, ethnic minorities, and patients in survivorship or palliative contexts. Health systems and policymakers should embed these interventions into community oncology, survivorship initiatives, and telehealth models to reduce disparities in psychosocial care.

Given rising global cancer incidence and associated psychological burden, we propose a tiered model in which distress screening triggers tailored psychological referrals. Policy reforms must recognize structured psychotherapies, mindfulness training, and resilience programs as reimbursable and include integrated psychosocial services among accreditation criteria for cancer centers. Shifting from reactive to preventive, personalized psychological care promises not only to relieve suffering but also to improve treatment adherence, quality of life, and potentially clinical outcomes, thereby fulfilling holistic oncology’s mandate.

### 4.6. Future Research Directions

This meta-analysis clarifies current psycho-oncology practices and highlights five future directions. First, individual participant data meta-analyses are needed to identify moderators and mediators—such as baseline distress, cancer type, coping style, and treatment phase—enabling personalized psychological care akin to precision pharmacotherapy. Second, methodological standardization is crucial: future RCTs should adopt uniform protocols for session length, therapist training, fidelity assessment, long-term follow-up, and active control conditions to reduce heterogeneity and bias. Third, cross-cultural and equity-focused research must extend beyond breast cancer and high-income settings to include prostate, gastrointestinal, adolescent, and low- to middle-income populations. Culturally adapted interventions and qualitative process evaluations will elucidate sociocultural influences on acceptability and outcomes. Fourth, rigorous comparisons of digital versus in-person delivery—across web-based platforms, mobile apps, and hybrid models—are essential to assess cost-effectiveness, engagement, and clinical impact, with artificial intelligence-driven tailoring offering promising avenues for scalable, personalized support. Fifth, mechanistic trials should integrate biological endpoints—cortisol, inflammatory cytokines, and telomere length—to explore mind-body pathways linking psychosocial interventions with immune function, treatment adherence, and survival.

Finally, emerging evidence underscores the interplay between emotional health, nutrition, and optimism in oncology: balanced diets may stabilize mood during treatment, and optimism enhances adherence to nutritional and therapeutic regimens. Integrating nutritional strategies with psychosocial care could improve quality of life and treatment outcomes [96,97,98]. These priorities call for smarter trials—mechanistically grounded, inclusive, technologically innovative, and clinically translatable—to establish psychosocial care as an essential pillar of comprehensive cancer treatment.

## 5. Conclusions

This systematic review and meta-analysis of 42 randomized controlled trials provides definitive evidence that specific non−pharmacological therapies-structured psychotherapies (Cognitive Behavioral Therapy, Acceptance and Commitment Therapy, Meaning−Centered Psychotherapy), mindfulness-based programs (Mindfulness-Based Stress Reduction, Mindfulness-Based Cognitive Therapy), and resilience-enhancing interventions (Promoting Resilience in Stress Management, expressive writing)–produce statistically robust and clinically meaningful reductions in distress, anxiety, depression, and maladaptive coping among adults with cancer. By delineating these modalities into theoretically coherent domains, identifying key moderators, and confirming cross-cultural applicability, this study moves from generic advocacy to precise implementation, offering a clear blueprint for personalization, cultural inclusivity, and exploration of biological and survival outcomes. Embedding CBT, ACT, MCP, MBSR, MBCT, PRISM, and expressive writing as core components of standard oncology pathways promises not only to optimize mental health but also to enhance patient experience and quality of life throughout the cancer trajectory.

## Figures and Tables

**Table 1:** Comparative summary of psychological intervention categories and their pooled efficacy on primary outcomes.

Intervention Category	Example Therapies	Primary Outcomes Assessed	General Efficacy Notes *
Structured Psychotherapeutic Interventions	Cognitive Behavioral Therapy (CBT); Acceptance and Commitment Therapy (ACT).	Depression, anxiety, psychological distress	Trend toward reduced distress but not statistically significant (t_14_ = −1.99, *p* = 0.066). Very high heterogeneity (I^2^ = 95%, τ^2^ = 0.9228). Supportive/expressive subgroups showed larger effects than CBT (SMD = −1.68 vs. −0.43), though differences were not significant.
Mindfulness-Based and Stress Reduction Programs	Mindfulness-Based Stress Reduction (MBSR); Mindfulness-Based Cognitive Therapy.	Distress, anxiety, fatigue, emotional regulation	Moderate, non-significant reductions in distress and anxiety (t_12_ = −1.58, *p* = 0.14). High heterogeneity (I^2^ = 87%, τ = 0.38). Notable improvements in fatigue and sleep (secondary outcomes). No clear evidence of publication bias.
Coping and Resilience-Enhancing Interventions	Promoting Resilience in Stress Management (PRISM); expressive writing.	Anxiety, depression, coping skills, psychological growth	Statistically significant moderate reduction in distress (t_13_ = −2.47, *p* = 0.028). High heterogeneity (I^2^ = 83%, τ^2^ = 0.33). After trim-and-fill adjustment (5 imputed studies), pooled effect attenuated to −0.09 (−0.54 to 0.35), indicating sensitivity to publication bias. Interventions often improved coping self-efficacy and quality of life.

* Note: Pooled effect sizes were calculated using a random-effects model with restricted maximum likelihood estimation. Effect size for the coping and resilience category remained significant before publication bias adjustment.

## Data Availability

No new data were created or analyzed in this study.

## Supplementary Materials

The following supporting information can be downloaded at: https://www.mdpi.com/article/10.3390/medicina61061086/s1, Table S1. Systematic Review Search Strategy: Structured Queries by Database; Table S2. Overview of Studies Included in the Qualitative and Quantitative Evidence Synthesis; Table S3. Excluded Studies from Qualitative and Quantitative Synthesis with Rationale Based on Eligibility Criteria; Table S4. Synthesis of Included Studies by Intervention Type: Summary of Key Characteristics; Table S5. Risk of Bias Assessment (RoB 2) and Certainty of Evidence Ratings (GRADE) for Included Randomized Controlled Trials; Table S6. Structured Psychotherapeutic Interventions—Effect Estimates (Forest Plot); Table S7. Assessment of Heterogeneity in Randomized Controlled Trials Using Funnel Plot Analysis (Structured Psychotherapeutic Interventions); Table S8. Cochran’s Q Test for Heterogeneity in Randomized Controlled Trials: Funnel Plot-Based Analysis (Structured Psychotherapeutic Interventions); Table S9. Mindfulness-Based and Stress Reduction Interventions—Effect Estimates (Forest Plot); Table S10. Assessment of Heterogeneity in Randomized Controlled Trials Using Funnel Plot Analysis (Mindfulness and Stress Reduction Interventions); Table S11. Cochran’s Q Test for Heterogeneity in Randomized Controlled Trials: Funnel Plot-Based Analysis (Mindfulness and Stress Reduction Interventions); Table S12. Coping and Psychological Resilience Interventions—Effect Estimates (Forest Plot); Table S13. Assessment of Heterogeneity in Randomized Controlled Trials Using Funnel Plot Analysis (Coping and Psychological Resilience Interventions); Table S14. Cochran’s Q Test for Heterogeneity in Randomized Controlled Trials: Funnel Plot-Based Analysis (Coping and Psychological Resilience Interventions); Table S15. Coping and Psychological Resilience Interventions (Trimm and Fill Plots); Table S16. Assessment of Heterogeneity in Randomized Controlled Trials Using Trimm and Fill Plots Analysis (Coping and Psychological Resilience Interventions); Table S17. Cochran’s Q Test for Heterogeneity in Randomized Controlled Trials: Trimm and Fill Plots-based Analysis (Coping and Psychological Resilience Interventions); Table S18. Meta-Subgroup Analysis—CBT-Based Interventions; Table S19. Assessment of Heterogeneity in Randomized Controlled Trials Using Funnel Plot Analysis (CBT-Based Interventions); Table S20. Cochran’s Q Test for Heterogeneity in Randomized Controlled Trials: Funnel Plot-Based Analysis (CBT-Based Interventions); Table S21. Meta-Subgroup Analysis—Supportive/Expressive Interventions; Table S22. Assessment of Heterogeneity in Randomized Controlled Trials Using Funnel Plot Analysis (Supportive/Expressive Interventions); Table S23. Cochran’s Q Test for Heterogeneity in Randomized Controlled Trials: Funnel Plot-Based Analysis (Supportive/Expressive Interventions); Table S24. Statistical Comparison Between CBT-Based and Supportive/Expressive Interventions (Subgroup Difference Test); Table S25. Effect Sizes for Meta-Regression Analysis of Structured Psychotherapeutic Interventions in Oncology (Hedges’ g).
