# Supplementary material for: Mind over Malignancy: A Systematic Review and Meta-Analysis of Psychological Distress, Coping, and Therapeutic Interventions in Oncology
Source: Medicina (Kaunas). 2025 Jun 13;61(6):1086. doi: 10.3390/medicina61061086 (PMC12195218; doi:10.3390/medicina61061086)
Supplement: Supplementary file 1 [file medicina-61-01086-s001.zip › medicina-3637202-supplementary.pdf]

Article,

Mind over Malignancy: Investigating Psychological Distress, Coping, and Therapeutic Responses in Oncology through Systematic Review and Meta-Analysis

Ana Maria Paslaru [1,2,†], Alina Plesea-Condratovici [2,†], Lavinia- Alexandra Moroianu [3,*], Oana- Maria Isailă [4,*], Laura Florentina Rebegea [5], Liliana Lacramioara Pavel [3] and Anamaria Ciubară [5]

[1] Doctoral School of Biomedical Sciences, "Dunărea de Jos" University, 800201 Galați, Romania; annapaslaru@gmail.com
[2] Medical Department, Faculty of Medicine and Pharmacy, Dunărea de Jos University, 800201 Galați, Romania; alina.plesea@ugal.ro
[3] Department of Pharmaceutical Sciences, Faculty of Medicine and Pharmacy, Dunărea de Jos University, 800201 Galați, Romania; doctorpavel2012@yahoo.com
[4] Department of Legal Medicine and Bioethics, Faculty of Dental Medicine, "Carol Davila" University of Medicine and Pharmacy, 020021 Bucharest, Romania
[5] Clinical Medical Department, Faculty of Medicine and Pharmacy, Dunărea de Jos University, 800201 Galați, Romania; laura.rebegea@ugal.ro (L.F.R.); anamburlea@yahoo.com (A.C.)
* Correspondence: lavinia.moroianu@yahoo.com (L.-A.M.); oana-maria.isaila@umfcd.ro (O.-M.I.)
† These authors contributed equally to this work.

SUPPLEMENTARY MATERIALS

Table S1. Systematic Review Search Strategy: Structured Queries by Database.

ID	Platform	Database	Resources	Search Strategy
**1**	Cochrane Library	Cochrane Central Register of Controlled Trials (CENTRAL)	Randomized controlled trials, Cochrane Reviews, protocols.	Combined keyword and MeSH strategy focused on psychological outcomes in oncology:<br>**Psychological terms**: ("depression" OR "anxiety" OR "distress" OR "coping" OR "resilience");<br>**Oncology terms**: ("cancer" OR "oncology" OR "neoplasm" OR "malignancy");<br>**Intervention terms**: ("psychotherapy" OR "mindfulness" OR "psychoeducation" OR "resilience training" OR "support group" OR "CBT" OR "ACT" OR "MBSR");<br>**Filters**: 2015–2025, Humans, Clinical Trials, Cochrane Reviews.
**2**	NCBI PubMed.	MEDLINE (PubMed Records)	Peer-reviewed biomedical literature.	Boolean strategy integrating MeSH and free-text terms:<br>**Condition terms:** ("cancer" OR "oncology" OR "malignancy");<br>**Mental health terms**: ("depression" OR "anxiety" OR "psychological distress" OR "resilience" OR "coping");<br>**Intervention terms:** ("CBT" OR "ACT" OR "MBSR" OR "psycho-oncology" OR "supportive-existential" OR "peer support").<br>**Filters**: 2015–2025, Humans, Randomized Controlled Trials, English.
**3**	ClinicalTrials.gov.	ClinicalTrials.gov Registry	Registered clinical trials, unpublished interventional research.	Advanced condition and intervention field search:<br>**Condition**: "Cancer" AND<br>**Intervention:** ("psychological support" OR "coping therapy" OR "mindfulness" OR "psychotherapy").<br>**Filters:** Interventional studies (RCTs); Status: Completed or **Recruiting**; Study start: 2015–2025.

ID	Platform	Database	Resources	Search Strategy
**4**	ProQuest (Dissertations and Theses-Central)	ProQuest Research Platforms	Grey literature: theses, dissertations, technical reports, unpublished data.	Keyword-driven search for unpublished psychosocial intervention trials:<br>**Keywords:** (“cancer” AND “depression” AND “psychological intervention”) OR (“mindfulness” OR “CBT” OR “resilience”);<br>Filters: Publication types: Dissertations, Theses, Scholarly Journals;<br>Time filter: 2015–2025.

References: All studies referenced herein are fully cited within the main body of the manuscript.

Table S2. Overview of Studies Included in the Qualitative and Quantitative Evidence Synthesis.

No.	ID	Design	Qualitative Synthesis (Yes/No)	Quantitative Synthesis (Yes/No)	Support
**1**	Bagherzadeh et al., 2022, Iran	Randomized Controlled Trials	Yes	Yes	The study met all eligibility criteria. **Population**: adult women diagnosed with breast cancer. **Intervention**: a standardized mindfulness-based stress reduction program delivered in eight sessions according to the original protocol. **Comparison**: a usual care control group with no psychological intervention. **Outcomes**: psychological distress measured through validated instruments assessing cognitive rumination. The trial employed a randomized design, ensured intervention fidelity, and reported effect size data suitable for inclusion in the meta-analysis.
**2**	Bower et al., 2015, USA	Randomized Controlled Trials	Yes	Yes	The study met all eligibility criteria. **Population:** adult premenopausal women diagnosed with early-stage breast cancer at or before age 50, who had completed cancer treatment at least three months prior. **Intervention:** a structured 6-week mindfulness-based program—Mindful Awareness Practices (MAPs)—consisting of weekly two-hour group sessions integrating theoretical education, experiential meditation, and psychoeducation tailored for young survivors. **Comparison**: a wait-list control group that did not receive any intervention during the study period. **Outcomes**: significant reductions in psychological stress and marginal reductions in depressive symptoms were observed post-intervention. Secondary benefits included improved sleep quality, reduced fatigue and menopausal symptoms, enhanced sense of peace and positive affect, and lowered proinflammatory gene expression. However, these psychological effects diminished at 3-month follow-up, with only sustained reductions in cancer-specific distress. The trial employed a randomized controlled design with rigorous biomarker analyses, ensuring the methodological robustness necessary for inclusion in the meta-analysis.

No.	ID	Design	Qualitative Synthesis (Yes/No)	Quantitative Synthesis (Yes/No)	Support
**3**	Breitbart et al., 2018, USA	Randomized Controlled Trials	Yes	Yes	The study met all eligibility criteria. **Population**: adult patients diagnosed with stage IV solid tumor cancers who reported clinically relevant psychological distress, defined as a score of 4 or greater on the Distress Thermometer. **Intervention**: a structured, manualized individual psychotherapy model known as Individual Meaning-Centered Psychotherapy, delivered in seven sessions by trained clinicians. The intervention focused on enhancing spiritual well-being, existential meaning, and psychosocial adjustment in the context of terminal illness. **Comparison**: two control conditions were employed—supportive psychotherapy consisting of nondirective emotional support across seven sessions, and enhanced usual care, which involved referral to standard psychosocial services and provision of educational materials without direct therapeutic contact. **Outcomes**: primary outcomes included spiritual well-being (measured using the Functional Assessment of Chronic Illness Therapy - Spiritual Well-being Scale), sense of meaning (Life Attitude Profile-Revised), and quality of life (McGill Quality of Life Questionnaire). Secondary outcomes comprised psychological symptoms such as anxiety and depression (Hospital Anxiety and Depression Scale), hopelessness, and desire for hastened death. Results indicated that the intervention produced significant improvements in spiritual well-being, existential meaning, and quality of life compared to both control groups. The trial's rigorous design, adherence to protocol fidelity, and validated outcome measures supported its inclusion in both the systematic review and the meta-analysis.

No.	ID	Design	Qualitative Synthesis (Yes/No)	Quantitative Synthesis (Yes/No)	Support
**4**	Cafaro et al., 2019, Italy	Randomized Controlled Trials	Yes	Yes	The study met all eligibility criteria. **Population**: adult patients (aged 18 years or older) with stage I to III breast or colon cancer who had completed adjuvant chemotherapy within the previous eight months and were disease-free at the last follow-up visit. **Intervention**: a guided disclosure protocol, consisting of three 20-minute structured writing sessions designed to facilitate both emotional expression and cognitive processing of the cancer experience, administered over a six-week period. **Comparison**: a control group that completed a generic writing task unrelated to the cancer experience, also structured as three 20-minute sessions over six weeks. **Outcomes**: the primary outcome was post-traumatic growth, measured by the Post-traumatic Growth Inventory. Secondary outcomes included constructed meaning (Constructed Meaning Scale), distress associated with traumatic events (Impact of Event Scale), and emotional distress (Hospital Anxiety and Depression Scale). Although the study was underpowered and the results were not statistically significant, trends suggested a potential positive impact of the guided writing intervention on post-traumatic growth and distress. The trial used a randomized design and maintained intervention fidelity, with sufficient follow-up and validated instruments for all measured outcomes.
**5**	Cheung et al., 2017, USA	Randomized Controlled Trials	Yes	Yes	The study met all eligibility criteria. **Population**: adult women diagnosed with metastatic breast cancer. **Intervention**: a five-session positive affect skill intervention titled "Lessons in Linking Affect and Coping," which involved teaching participants empirically supported techniques to increase positive affect, including gratitude, mindfulness, positive reappraisal, savoring, personal strengths awareness, achievable goal setting, and acts of kindness. **Comparison**: an attention-matched control group receiving in-person sessions without active psychological skills training. **Outcomes**: psychological well-being (depression, positive and negative affect, cancer-specific quality of life) and positive coping (mindfulness, self-compassion, and positive-affect skill use) assessed through validated self-report measures. The study used a randomized controlled design and demonstrated good feasibility and acceptability. Within-group analysis showed significant reductions in depressive symptoms and negative affect among intervention participants at one-month follow-up, with medium to large effect sizes. The online format was as effective as in-person delivery, supporting the potential for scalable digital implementation.

No.	ID	Design	Qualitative Synthesis (Yes/No)	Quantitative Synthesis (Yes/No)	Support
**6**	Cillessen et al., 2018, Netherlands	Randomized Controlled Trials	Yes	Yes	The study met all eligibility criteria. **Population**: adult cancer patients experiencing at least mild psychological distress, diagnosed with various cancer types and stages. **Intervention**: two forms of mindfulness-based cognitive therapy, namely group-based in-person sessions and individual internet-based sessions, both following a standardized eight-week structure adapted slightly for cancer-related needs. **Comparison**: the two intervention modalities were compared head-to-head, with patients initially randomized also to a treatment-as-usual group which was later allocated to one of the interventions after a three-month delay. **Outcomes**: the primary outcome was psychological distress, with secondary outcomes including fear of cancer recurrence, rumination, positive mental health, and health-related quality of life. Both interventions demonstrated sustained long-term benefits, but the individual internet-based format was associated with slightly better outcomes in reducing psychological distress over a nine-month follow-up. The randomized controlled design, standardized intervention delivery, and inclusion of follow-up assessments enhance the study’s validity and suitability for meta-analytical integration.

No.	ID	Design	Qualitative Synthesis (Yes/No)	Quantitative Synthesis (Yes/No)	Support
**7**	Duval et al., 2022; Canada	Randomized Controlled Trials	Yes	Yes	The study met all eligibility criteria. **Population**: adult female breast cancer survivors experiencing chronic neuropathic pain following chemotherapy, at least one year post-treatment, with moderate to severe baseline pain scores. **Intervention**: a standardized mindfulness-based stress reduction program comprising eight weekly 2.5-hour sessions and one 6-hour retreat, adapted slightly for chronic pain populations but following the established structure, facilitated by professionals with experience in mindfulness and chronic pain. **Comparison**: a waitlist control group that received the same intervention only after a 3-month follow-up, allowing for evaluation of effects in the absence of the mindfulness program. **Outcomes:** subjective memory difficulties (retrospective and prospective) and general perceived cognitive function assessed using validated instruments such as the Functional Assessment of Cancer Therapy - Cognitive Function and the Prospective and Retrospective Memory Questionnaire. The intervention group showed significant short-term improvements in perceived memory functions (2 weeks post-intervention), although these effects were not sustained at the 3-month follow-up. Objective measures of cognitive performance, however, did not reveal a differential benefit from the intervention. The trial employed a randomized controlled design, ensured intervention fidelity, and reported comprehensive outcome data suitable for meta-analytic inclusion.

No.	ID	Design	Qualitative Synthesis (Yes/No)	Quantitative Synthesis (Yes/No)	Support
**8**	Fauser et al., 2023, Germany	Randomized Controlled Trials	Yes	Yes	The study met all eligibility criteria. **Population:** Adult women with a history of breast cancer who completed initial cancer treatment and demonstrated psychological distress (a score of at least five on the Distress Thermometer) at the start of a rehabilitation program in Germany. **Intervention**: A structured short-term psychotherapy delivered in a group format across three weekly sessions during a multi-modal inpatient rehabilitation program. The intervention incorporated psychoeducational elements, mindfulness, cognitive-behavioral strategies, and emotional self-regulation techniques. **Comparison**: A non-specific group discussion of the same duration and frequency, where participants freely discussed psychological and practical issues related to breast cancer without structured therapeutic techniques. **Outcomes**: The primary outcome was anxiety, measured using the Hospital Anxiety and Depression Scale. Secondary outcomes included depression, psychological distress, cancer-related fatigue, and various domains of health-related quality of life assessed with validated instruments. Although no significant differences were found between groups overall, a subgroup analysis revealed that participants with high baseline anxiety experienced a greater reduction in depressive symptoms following structured short-term psychotherapy. The trial was randomized and followed ethical standards, with adequate intervention fidelity and power calculations to support subgroup findings.
**9**	Graboyes et al., 2023, USA	Randomized Controlled Trials	Yes	Yes	The study met all eligibility criteria. **Population**: adult survivors of head and neck cancer presenting with clinically significant body image-related distress following treatment. **Intervention**: a tailored brief cognitive behavioral therapy program titled BRIGHT (Building a Renewed ImaGe after Head & neck cancer Treatment), consisting of five weekly psychologist-led video-based sessions targeting maladaptive body image coping. **Comparison**: a dose- and delivery-matched telehealth attention control condition providing general survivorship education without therapeutic elements. **Outcomes**: reduction in body image-related distress as measured by validated tools (IMAGE-HN and Body Image Scale), with mediation analysis showing significant improvement through the reduction of avoidant and appearance-fixing body image coping behaviors. The study utilized a randomized clinical trial design, maintained high intervention fidelity, and provided detailed outcome effect size data, qualifying it for meta-analytic inclusion.

No.	ID	Design	Qualitative Synthesis (Yes/No)	Quantitative Synthesis (Yes/No)	Support
**10**	Graham et al., 2024, UK	Randomized Controlled Trials	Yes	Yes	The study met all eligibility criteria. **Population**: adult women with early-stage (stage 1 to 3a) breast cancer who had completed hospital-based treatment (surgery, radiotherapy, and/or chemotherapy) within the past six months and were prescribed adjuvant endocrine therapy. **Intervention**: a remotely delivered Acceptance and Commitment Therapy intervention, including one individual session and three group sessions led by clinical psychologists, supplemented with a website providing side effect management resources and psychological flexibility exercises. **Comparison**: usual care, consisting of standard post-treatment care including a treatment summary meeting, holistic needs assessment, and access to a breast cancer nurse on an open referral basis. **Outcomes**: improvements in medication adherence (measured with the Adherence Starts with Knowledge questionnaire), health-related quality of life (assessed via the Functional Assessment of Cancer Therapy and the McGill Quality of Life Questionnaire), psychological distress (evaluated with the Generalized Anxiety Disorder-7 and the Patient Health Questionnaire-9), and psychological flexibility (measured with the Valuing Questionnaire). The trial showed good intervention fidelity and acceptability, indicating feasibility for a future phase III trial.
**11**	Gu et al., 2024, China	Randomized Controlled Trials	Yes	Yes	The study met all eligibility criteria. **Population:** adult women diagnosed with cervical cancer undergoing post-treatment rehabilitation in a tertiary hospital in China. **Intervention**: a standardized mindfulness-based stress reduction program delivered online over eight weeks, incorporating mindfulness breathing, body scan, yoga, and meditation exercises, tailored to enhance self-awareness, coping, and emotional regulation. **Comparison**: a control group receiving routine nursing care without any psychological intervention during the initial study period. **Outcomes**: primary outcome was cancer-related fatigue, assessed using the Cancer Fatigue Scale; secondary outcomes included uncertainty in illness, coping styles (facing, avoidance, yielding), sense of coherence, and perceived social support. The trial utilized a randomized controlled design with multiple follow-up assessments at post-intervention, three months, and six months. The mindfulness-based stress reduction program significantly improved fatigue, psychological resilience, and coping styles, with effects sustained over time, although perceived social support did not significantly differ between groups. The design ensured methodological rigor, enabling reliable effect size estimation for meta-analytic inclusion.

No.	ID	Design	Qualitative Synthesis (Yes/No)	Quantitative Synthesis (Yes/No)	Support
**12**	Gudenkauf et al., 2015, USA	Randomized Controlled Trials	Yes	Yes	The study met all eligibility criteria. **Population**: adult women diagnosed with stage 0–III non-metastatic breast cancer, recruited within 10 weeks post-surgery from community clinics and cancer centers in Miami, Florida. **Intervention**: two distinct 5-week group-based interventions—a cognitive-behavioral training focused on adaptive coping, cognitive restructuring, interpersonal skills, and assertiveness, and a relaxation training intervention teaching techniques such as progressive muscle relaxation, guided imagery, and meditation. **Comparison**: a time and attention-matched health education control group receiving cancer-related educational content without therapeutic skills training. **Outcomes**: indicators of psychological adaptation including depressive affect, cancer-specific cognitive intrusions, emotional well-being, and social disruption, alongside stress management resources like perceived social support and confidence in relaxation abilities. The randomized controlled trial design ensured methodological rigor, with blinded assessments, protocol adherence monitoring, and effect size reporting appropriate for meta-analytic inclusion.
**13**	Han et al., 2021, China	Randomized Controlled Trials	Yes	Yes	The study met all eligibility criteria. **Population**: adult patients in China newly diagnosed with advanced cancer, receiving palliative radiation therapy, aged over 20, and exhibiting clinically relevant psychological distress as indicated by a distress thermometer score above 4. **Intervention**: a structured combination of Naikan therapy and Morita therapy administered over seven weeks, incorporating 20 two-hour sessions of introspective Naikan therapy and sequential phases of Morita therapy targeting emotional regulation and behavioral activation. **Comparison**: a control group receiving standard medical care without any psychological intervention during the study period. **Outcomes:** primary outcomes included psychological distress measured by the distress thermometer and posttraumatic growth assessed through a culturally adapted version of the Posttraumatic Growth Inventory. Significant reductions in distress and improvements in growth domains such as relationships, personal strength, and appreciation of life were reported in the intervention group, with adherence rates exceeding 89%. The trial was randomized, adhered to intention-to-treat principles, and included statistical adjustments using analysis of covariance to control for baseline differences.

No.	ID	Design	Qualitative Synthesis (Yes/No)	Quantitative Synthesis (Yes/No)	Support
**14**	Huang et al., 2024, China	Randomized Controlled Trials	Yes	Yes	The study met all eligibility criteria. **Population**: adult patients diagnosed with esophageal or gastric cancer, recruited from a hospital oncology department in China, without prior psychological intervention and with significant psychological distress (score ≥4 on the Psychological Distress Thermometer). **Intervention**: an eight-session behavioral activation program, delivered primarily via telephone or WeChat, designed to increase engagement in meaningful and pleasurable activities and reduce avoidance behaviors, based on the Brief Behavioral Activation Treatment for Depression manual. **Comparison**: standard cancer care alone, involving usual medical and nursing support without any structured psychological intervention. **Outcomes**: psychological distress measured using the Psychological Distress Thermometer; anxiety symptoms assessed via the Generalized Anxiety Disorder 7-item scale; self-efficacy measured with the General Self-Efficacy Scale; and behavioral activation assessed using the Behavioral Activation for Depression Scale (activation subscale). The study used a randomized controlled trial design, ensured fidelity through audio recording and quality checks, and reported effect sizes, mediation analysis, and subgroup analyses by cancer type. The results demonstrated significant reductions in psychological distress and anxiety, along with improved self-efficacy and activation in the intervention group compared to the control. Self-efficacy mediated the effect of behavioral activation on psychological distress.
**15**	Isaka et al., 2021, Japan	Randomized Controlled Trials	Yes	Yes	The study met all eligibility criteria. **Population**: Adult women aged 20–69 years attending cervical cancer screening in Tsukuba City, Japan. **Intervention**: Provision of a comprehensive educational leaflet accompanying a hypothetical positive cervical cancer screening result, designed to clarify the implications and reduce psychological distress. **Comparison**: Control group receiving only a notification of a hypothetical positive screening result without any accompanying explanatory material. **Outcomes**: Primary outcome was psychological distress assessed using the Cancer Worry Scale, and the secondary outcome was intention to undergo further diagnostic examination. The randomized controlled trial demonstrated that the intervention group experienced significantly less psychological distress (odds ratio: 2.57, 95% confidence interval: 1.87–3.54) compared to controls, while both groups maintained a similarly high intention to pursue further examination (95% in the intervention group vs. 97% in the control group). This supports the leaflet's effectiveness in reducing distress without deterring medical follow-up.

No.	ID	Design	Qualitative Synthesis (Yes/No)	Quantitative Synthesis (Yes/No)	Support
**16**	Jensen-Johansen et al., 2018, Denmark	Randomized Controlled Trials	Yes	Yes	The study met all eligibility criteria. **Population**: Adult women (aged 27–70 years) recently treated for early-stage (Stage I and II) invasive breast cancer in Denmark, post-surgery (mastectomy or lumpectomy) and adjuvant treatment, and able to read and write Danish. **Intervention**: A structured expressive writing intervention consisting of three 20-minute, home-based sessions, one week apart, focusing on emotional disclosure about a distressing experience, including but not limited to their own cancer. The protocol followed the established expressive writing procedure and was facilitated through telephone support by trained assistants. **Comparison**: A control group instructed to engage in emotionally neutral writing about daily activities, matched in session structure and timing. **Outcomes**: The primary outcomes were self-reported physical symptoms and healthcare utilization, specifically the number of visits and telephone contacts with general practitioners, assessed at baseline, and 3 and 9 months post-intervention. Results showed no significant main effects for the intervention. However, subgroup analyses indicated beneficial effects among women low in alexithymia and those who wrote about their own cancer, suggesting these as potential moderators. The trial used a randomized controlled design and reported outcome data amenable for inclusion in meta-analytic synthesis.

No.	ID	Design	Qualitative Synthesis (Yes/No)	Quantitative Synthesis (Yes/No)	Support
**17**	Johns et al., 2015, USA	Randomized Controlled Trials	Yes	Yes	The study met all eligibility criteria. **Population**: adult cancer survivors who experienced clinically significant cancer-related fatigue for at least eight weeks and had completed active cancer treatment (excluding endocrine therapy) at least three months prior to enrollment. **Intervention**: a structured seven-week program based on mindfulness-based stress reduction, incorporating meditation, yoga, and psychoeducation tailored to fatigue management. The sessions included training in body scan, sitting and walking meditation, compassion practices, and mindful movement. Home practice was encouraged with guided materials. **Comparison**: a wait-list control group with delayed access to the intervention post-follow-up. **Outcomes**: the primary outcome was fatigue interference, measured using the Fatigue Symptom Inventory interference subscale. Secondary outcomes included fatigue severity, vitality, depression, anxiety, functional disability, and sleep disturbance. The trial employed a randomized controlled design, ensured fidelity to the mindfulness-based stress reduction curriculum, and provided comprehensive effect size data for analysis. Significant improvements were observed in all measured outcomes in the intervention group compared to controls, with sustained benefits at six-month follow-up.
**18**	Johns et al., 2016, USA	Randomized Controlled Trials	Yes	Yes	The study met all eligibility criteria. **Population**: Adult survivors of stage 0–III breast or colorectal cancer, previously treated with chemotherapy and/or radiotherapy, experiencing moderate-to-severe cancer-related fatigue for at least 8 weeks. **Intervention:** A structured eight-week mindfulness-based stress reduction program, including formal mindfulness meditation practices (e.g., body scan, mindful movement, sitting meditation), adapted slightly in duration for fatigued cancer survivors and delivered by certified instructors. **Comparison**: An active control group that received education and support through weekly sessions focusing on symptom management and survivorship topics, led by oncology social workers. **Outcomes**: Cognitive functioning measured subjectively using the Attentional Function Index and objectively via the Stroop color-word test. The mindfulness-based stress reduction group demonstrated significantly greater improvements in attentional function and error reduction on the Stroop test at post-intervention and six-month follow-up compared to the control group. Intervention fidelity was maintained, and effect sizes were reported, supporting inclusion in the meta-analysis.

No.	ID	Design	Qualitative Synthesis (Yes/No)	Quantitative Synthesis (Yes/No)	Support
**19**	Kenne Sarenmalm et al., 2017, Sweden	Randomized Controlled Trials	Yes	Yes	The study met all eligibility criteria. **Population**: Adult women diagnosed with breast cancer, post-adjuvant chemotherapy and/or radiation therapy, with or without endocrine therapy. **Intervention**: A standardized mindfulness-based stress reduction program delivered in eight weekly group sessions including meditation, yoga, and supportive group discussion, with home practice supported by structured materials and audio recordings. **Comparison**: Two comparator groups: one with a self-instructed mindfulness-based stress reduction program (active control) and another with no psychological intervention (non-mindfulness-based stress reduction control). **Outcomes:** Primary outcomes included mood disorders (depression and anxiety), assessed with the Hospital Anxiety and Depression Scale. Secondary outcomes encompassed symptom burden, distress, physical and mental health (via Short Form-36 Health Survey), coping capacity (Sense of Coherence scale), mindfulness (Five Facets of Mindfulness Questionnaire), posttraumatic growth (Posttraumatic Growth Inventory), and biological markers such as natural killer cell activity and cytokine levels (IL-6, IL-8). The randomized controlled trial reported significant improvements in depression, psychological symptom burden, mental health, coping capacity, and biological immune response in the intervention group compared to controls.
**20**	Lengacher et al., 2016, USA	Randomized Controlled Trials	Yes	Yes	The study met all eligibility criteria. **Population**: Adult female survivors of stage 0 to III breast cancer who had completed treatment within the previous 2 weeks to 2 years. **Intervention**: A six-week mindfulness-based stress reduction program specifically adapted for breast cancer survivors, which included weekly 2-hour sessions of meditation, yoga, and psychoeducation, along with home practice materials. **Comparison**: A usual care group that received no active psychological intervention but was waitlisted to receive the program post-study. **Outcomes:** Psychological symptoms (anxiety, perceived stress, fear of recurrence, depression) and physical symptoms (fatigue, pain), along with quality of life were measured at baseline, 6 weeks, and 12 weeks using validated scales. The study reported statistically significant improvements in anxiety, fear of recurrence, and fatigue severity and interference, with small to moderate effect sizes. These effects were sustained at the 12-week follow-up. The study employed a rigorous randomized controlled trial design with fidelity checks and moderator analysis showing that those with higher baseline stress experienced greater benefits.

No.	ID	Design	Qualitative Synthesis (Yes/No)	Quantitative Synthesis (Yes/No)	Support
**21**	Li et al., 2023, China	Randomized Controlled Trials	Yes	Yes	The study met all eligibility criteria. **Population**: adolescent and young adult cancer patients aged 15–39 years, diagnosed with malignant tumors in the last 5 years, experiencing psychological distress. **Intervention**: an 8-week intelligent physical activity program incorporating moderate-intensity exercise tracked by wearable smart devices, following recommendations from the American Cancer Society, including goal-setting, exercise diaries, and social interaction via mobile apps. **Comparison**: a treatment-as-usual control group receiving standard psychological nursing care and another group undergoing a web-based, modified behavioral activation program involving weekly video seminars and structured tasks. **Outcomes**: primary outcomes included changes in salivary cortisol and testosterone levels, with secondary outcomes comprising anxiety and depression (measured via the Hospital Anxiety and Depression Scale), physical activity (via International Physical Activity Questionnaire), self-efficacy, sleep quality, and social support. The trial was randomized, reported effect sizes, used validated outcome instruments, and allowed comparative evaluation at 1-week and 3-month follow-ups.
**22**	Lopez et al., 2023, USA	Randomized Controlled Trials	Yes	Yes	The study met all eligibility criteria. **Population**: adult cancer patients experiencing moderate symptoms of anxiety and/or depression, primarily women with breast cancer, receiving care at the MD Anderson Cancer Center in the United States. **Intervention**: a two-week, self-administered, smartphone-based meditation application that included 5-, 10-, or 15-minute guided meditation audio sessions designed to enhance mood and reduce distress. **Comparison**: a waitlist control group receiving usual care without the intervention during the study period. **Outcomes**: primary outcome was feasibility of the intervention (recruitment and adherence rates); secondary outcomes included changes in psychological distress, sleep quality, and mood assessed using standardized scales such as the Edmonton Symptom Assessment Scale (ESAS), Hospital Anxiety and Depression Scale (HADS), and Pittsburgh Sleep Quality Index (PSQI). The intervention group showed statistically and clinically significant improvements in fatigue, depression, anxiety, appetite, and various distress subscales. These findings suggest potential clinical benefit and feasibility for broader implementation in future trials.

No.	ID	Design	Qualitative Synthesis (Yes/No)	Quantitative Synthesis (Yes/No)	Support
**23**	Lu et al., 2023, USA	Randomized Controlled Trials	Yes	Yes	The study met all eligibility criteria. **Population**: Adult Chinese American women who had completed primary treatment for breast cancer within the past five years and were fluent in Mandarin or Cantonese. **Intervention**: A culturally adapted expressive writing intervention known as the enhanced self-regulation condition, which involved three weekly writing sessions focusing sequentially on stress and coping, emotional expression, and benefit finding. **Comparison**: Two control groups—one received a cancer-facts writing condition (factual writing only), and the other a standard self-regulation writing condition where emotional disclosure preceded cognitive reappraisal. **Outcomes:** Depressive and anxiety symptoms measured at 1, 3, and 6 months using validated instruments. The enhanced self-regulation condition demonstrated significant improvements in both outcomes compared to the control group, with reductions mediated by decreased perceived stress. The trial was randomized, incorporated cultural tailoring through stakeholder engagement, and reported statistically and clinically relevant outcome data appropriate for meta-analytic synthesis.
**24**	Manne et al., 2017, USA	Randomized Controlled Trials	Yes	Yes	The study met all eligibility criteria. **Population**: adult women newly diagnosed with gynecological cancer (including cervical, endometrial, ovarian, or uterine cancers), within six months of diagnosis, possessing good functional status (Karnofsky Performance Status > 80 or Eastern Cooperative Oncology Group score of 0 or 1), and residing within two hours of a participating center in the Northeastern United States. **Intervention**: a structured coping and communication-enhancing psychological intervention consisting of seven individual weekly sessions and one telephone booster session. The intervention focused on enhancing coping skills, communication, emotional expression, and managing fears of recurrence, and was based on cognitive-affective-social processing theory. **Comparison**: two control arms—supportive counseling intervention with the same schedule but non-directive in nature, and a usual care group receiving standard psychosocial care including access to social work services. **Outcomes**: psychological outcomes including depression (measured via Beck Depression Inventory), cancer-specific distress (Impact of Events Scale), fear of recurrence (Concerns About Recurrence Scale), and emotional well-being (Functional Assessment of Cancer Therapy – Emotional Functioning subscale). The coping and communication-enhancing intervention showed greater improvements in depression, distress, and emotional well-being than either supportive counseling or usual care, particularly over the first six months.

No.	ID	Design	Qualitative Synthesis (Yes/No)	Quantitative Synthesis (Yes/No)	Support
**25**	Marziliano et al., 2023, USA	Randomized Controlled Trials	Yes	Yes	The study met all eligibility criteria. **Population:** Adult women newly diagnosed with early-stage, non-metastatic breast cancer, within 60 days of diagnosis, residing in the United States, with computer access and no prior treatment decision made. **Intervention**: A multimedia, theory-based interactive decision-support program named Healing Choices, designed to aid treatment decision-making through educational modules, peer testimonials, and physician communication tools. **Comparison**: A control group receiving standard National Cancer Institute educational print materials without access to the multimedia software. **Outcomes**: Primary outcomes included levels of decisional conflict and psychological distress, measured at two months post-intervention. While the intent-to-treat analysis revealed no significant reduction in decisional conflict and a marginal increase in distress in the intervention group, as-treated analyses indicated increased perceived decisional support among users of the intervention with no adverse effect on distress. The study employed a randomized controlled design, included psychometrically validated assessment tools, and reported sufficient statistical data for meta-analytic integration.
**26**	Mirmahmoodi et al., 2020, Iran	Randomized Controlled Trials	Yes	Yes	The study met all eligibility criteria. **Population**: adult women diagnosed with breast cancer without metastasis, aged between 18 and 70 years, undergoing at least one chemotherapy period or surgery, and with no history of psychological treatment, psychiatric disorders, or use of corticosteroids or psychotropic medications. **Intervention**: an eight-week group-based mindfulness-based stress reduction program delivered in 90-minute weekly sessions by a trained facilitator, focusing on awareness, body scanning, mindful breathing, and meditative practices. **Comparison**: a routine care control group receiving no structured psychological intervention, but with access to psychiatric consultation on request. **Outcomes**: psychological responses were assessed using validated instruments measuring anxiety (Beck Anxiety Inventory), depression (Beck Depression Inventory-II), and stress (Perceived Stress Scale), along with biological markers of inflammation (cortisol and C-reactive protein). The intervention significantly improved anxiety levels but did not produce statistically significant changes in stress, depression, or inflammatory markers. The trial applied a randomized design with block randomization and controlled for confounding treatment variables in the analysis.

No.	ID	Design	Qualitative Synthesis (Yes/No)	Quantitative Synthesis (Yes/No)	Support
**27**	Nairn & Merluzzi, 2019, USA	Randomized Controlled Trials	Yes	Yes	The study met all eligibility criteria. **Population**: Adults undergoing active treatment for various cancer types who reported low confidence in their ability to cope with the disease. **Intervention:** A structured, brief psychological therapy termed Mastery Enhancement Therapy, consisting of four tailored sessions based on self-regulation and self-efficacy theories. The therapy aimed to increase coping skills by focusing on patients' previously successful coping behaviors. **Comparison**: A control group that received usual care without additional psychological intervention, but completed the same assessments. **Outcomes**: Improvements in coping self-efficacy, psychological adjustment, and reductions in depressive symptoms. Notably, the intervention group showed accelerated improvements in coping self-efficacy and, for those with higher symptom burdens, superior outcomes in psychological adjustment and depression at 3-month follow-up compared to controls. The study employed a randomized controlled design and ensured treatment fidelity through session monitoring. Effect sizes supported moderate intervention efficacy.
**28**	Nelson et al., 2021, USA	Randomized Controlled Trials	Yes	Yes	The study met all eligibility criteria. **Population**: adult patients hospitalized with newly diagnosed or relapsed/refractory high-risk acute myeloid leukemia, undergoing intensive chemotherapy regimens typically requiring a 3–6 week inpatient stay. **Intervention**: a structured integrated palliative and oncology care program, involving at least twice-weekly consultations with palliative care specialists during initial and subsequent hospitalizations, addressing symptom management, coping strategies, illness understanding, treatment decision-making, and end-of-life care planning. **Comparison**: a standard usual care control group that received routine leukemia management without mandatory palliative involvement, though ad hoc palliative consultations were allowed. **Outcomes**: psychological outcomes (depression and anxiety symptoms) and quality of life, evaluated at baseline and multiple follow-ups through standardized instruments including the Hospital Anxiety and Depression Scale and Functional Assessment of Cancer Therapy–Leukemia scale; coping was assessed through the Brief Coping Orientation to Problems Experienced Inventory. The randomized design, high intervention fidelity, and mediation analyses support inclusion in meta-analytical evaluations.

No.	ID	Design	Qualitative Synthesis (Yes/No)	Quantitative Synthesis (Yes/No)	Support
**29**	Nissen et al., 2020, Denmark	Randomized Controlled Trials	Yes	Yes	The study met all eligibility criteria. **Population**: adult breast and prostate cancer survivors experiencing symptoms of anxiety and/or depression, more than 3 months and up to 5 years post-primary treatment, recruited through routine oncology follow-up in Denmark. **Interventio**n: a therapist-assisted internet-delivered mindfulness-based cognitive therapy program, developed with cancer survivors' input, comprising eight weekly modules with written, audio, and video content, asynchronous therapist feedback, and adapted for cancer survivorship needs. **Comparison**: a usual care wait-list control group receiving no psychological intervention during the study period. **Outcomes:** primary outcomes were anxiety and depression assessed at baseline, post-intervention, and six months follow-up using standardized scales (State-Trait Anxiety Inventory and Beck Depression Inventory-II). The intervention yielded statistically significant reductions in anxiety and depression at post-intervention (effect sizes d = 0.45 and d = 0.42 respectively), with maintained effects on anxiety (d = 0.40) but not depression at follow-up. The trial used a randomized design, stratified by cancer type, with rigorous adherence monitoring and sensitivity analyses. Effect size data were provided, supporting inclusion in the meta-analysis.

No.	ID	Design	Qualitative Synthesis (Yes/No)	Quantitative Synthesis (Yes/No)	Support
**30**	Park et al., 2020, Japan	Randomized Controlled Trials	Yes	Yes	The study met all eligibility criteria. **Population**: Adult women diagnosed with Stage 0 to III breast cancer, attending as outpatients at a tertiary medical facility in Tokyo, Japan, and experiencing psychological distress, defined by a Hospital Anxiety and Depression Scale score of five or greater. **Intervention:** An eight-week mindfulness-based cognitive therapy program delivered in group sessions, adapted from the original protocol with structured psychoeducational and meditative components tailored for breast cancer patients, including mindfulness exercises, cognitive therapy elements, and daily homework. **Comparison**: A wait-list control group instructed to refrain from any mindfulness-based practices during the study period. **Outcomes**: Significant reductions in psychological distress (anxiety and depression), fear of cancer recurrence, and fatigue, along with improvements in spiritual well-being and overall quality of life. Outcomes were measured using validated instruments including the Hospital Anxiety and Depression Scale, Concerns About Recurrence Scale, Brief Fatigue Inventory, Functional Assessment of Chronic Illness Therapy-Spiritual, and Functional Assessment of Cancer Therapy-General. The effects were maintained at four weeks post-intervention, with reported effect sizes ranging from small to large across all domains. The trial adhered to a randomized design, maintained high intervention fidelity, and provided effect size data suitable for meta-analytic inclusion.
**31**	Reich et al., 2017, USA	Randomized Controlled Trials	Yes	Yes	The study met all eligibility criteria. **Population**: adult women who had completed treatment for Stage 0 to III breast cancer within two weeks to two years prior to enrollment. **Intervention**: a structured six-week mindfulness-based stress reduction program specifically adapted for breast cancer patients, including meditation practices, relaxation techniques, and group discussions, delivered weekly by a trained psychologist. **Comparison**: a usual care group that continued standard post-treatment clinical visits without engaging in mindfulness-based interventions during the study period. **Outcomes**: psychological symptoms (depression, anxiety, stress, and emotional well-being), fatigue (fatigue severity, sleep quality, and drowsiness), pain (intensity and interference), and cognition (memory and mindfulness) measured with validated instruments. The randomized controlled trial reported significant improvements in psychological and fatigue symptom clusters at six weeks, with sustained effects through twelve weeks, and included effect size data appropriate for inclusion in the meta-analysis.

No.	ID	Design	Qualitative Synthesis (Yes/No)	Quantitative Synthesis (Yes/No)	Support
**32**	Rodin et al., 2018, Canada	Randomized Controlled Trials	Yes	Yes	The study met all eligibility criteria. **Population**: adults diagnosed with advanced or metastatic cancers including stage III or IV lung cancer, advanced gastrointestinal, breast, gynecologic, genitourinary, endocrine, or soft tissue cancers, with an expected prognosis of 12 to 18 months. **Intervention**: a structured psychotherapeutic approach called Managing Cancer and Living Meaningfully (CALM), comprising 3–6 semi-structured sessions over a period of 3 to 6 months, tailored to address domains including symptom management, changes in identity and relationships, spiritual well-being, and preparation for end of life. **Comparison**: usual care consisting of standard oncology treatment with distress screening and optional psychosocial referrals without a structured psychotherapeutic framework. **Outcomes**: primary outcome was depressive symptom severity as measured by the Patient Health Questionnaire-9. Secondary outcomes included anxiety, spiritual well-being, death-related distress, quality of life at end of life, attachment security, couple communication, post-traumatic growth, and demoralization. The randomized controlled trial demonstrated significant reductions in depressive symptoms and improvements in end-of-life preparation in the intervention group at both 3 and 6 months.
**33**	Rosenberg et al., 2018, USA	Randomized Controlled Trials	Yes	Yes	The study met all eligibility criteria. **Population**: Adolescents and young adults aged 12 to 25 years diagnosed with cancer, including leukemia, lymphoma, central nervous system, and solid tumors, who were fluent in English and receiving systemic chemotherapy or had progressive, recurrent, or refractory cancer. **Intervention**: The Promoting Resilience in Stress Management program, a developmentally tailored, manualized, skills-based psychosocial intervention delivered over four sessions, targeting stress management, goal setting, cognitive reframing, and meaning making. **Comparison**: A usual care control group that included ad hoc psychosocial services provided by an assigned social worker and access to multidisciplinary support upon request. **Outcomes:** Patient-reported resilience (measured by the 10-item Connor-Davidson Resilience Scale), quality of life (measured by Pediatric Quality of Life Generic and Cancer Modules), and psychological distress (measured by the Kessler-6 Psychological Distress Scale and the Hospital Anxiety and Depression Scale). The intervention group showed improved resilience and cancer-specific quality of life, along with reduced psychological distress compared to the control group. The trial used a randomized controlled design, maintained intervention fidelity, and reported clinically meaningful effects on psychosocial outcomes.

No.	ID	Design	Qualitative Synthesis (Yes/No)	Quantitative Synthesis (Yes/No)	Support
**34**	Rosenberg et al., 2021, USA	Randomized Controlled Trials	Yes	Yes	The study met all eligibility criteria. **Population**: Adolescents and young adults between 13 and 25 years old, diagnosed with newly diagnosed or progressive cancer and treated at a quaternary children's hospital in the United States. **Intervention**: The Promoting Resilience in Stress Management program, a structured, four-session, skills-based coaching intervention delivered one-on-one by trained nonclinical staff. The sessions targeted stress management, goal setting, cognitive reframing, and meaning-making, with supporting worksheets between sessions. **Comparison:** A control group receiving psychosocial usual care, which included assessments and ad hoc support from social workers and mental health professionals. **Outcomes:** Improvements in patient-reported measures of cancer-related quality of life, hope, resilience, and psychological distress were assessed at baseline, 6, 12, and 24 months. The intervention group showed significant and sustained benefits in quality of life and hope compared to the control group over the two-year period, although effects on resilience and psychological distress were not statistically significant in long-term follow-up.
**35**	Ross et al., 2016, USA	Randomized Controlled Trials	Yes	Yes	The study met all eligibility criteria. **Population:** Adult individuals diagnosed with life-threatening cancer who exhibited clinically significant symptoms of anxiety and/or depression, as assessed by standardized diagnostic interviews and validated self-report instruments. **Intervention:** A single moderate dose (0.3 mg/kg) of psilocybin administered in conjunction with a structured psychotherapeutic support protocol. The intervention emphasized preparation, dosing, and integration psychotherapy sessions to enhance safety and therapeutic impact. **Comparison**: An active control group receiving niacin (250 mg) under identical psychotherapeutic conditions. The study employed a randomized, double-blind, placebo-controlled, crossover design to rigorously compare the interventions. **Outcomes**: The primary outcomes were reductions in anxiety and depression symptoms, measured using the Hospital Anxiety and Depression Scale, Beck Depression Inventory, and the State-Trait Anxiety Inventory. Psilocybin was associated with immediate and sustained anxiolytic and antidepressant effects up to 6.5 months post-intervention. Secondary outcomes included improvements in existential distress, quality of life, and spiritual wellbeing. The intensity of the psilocybin-induced mystical experience was found to mediate therapeutic outcomes. The trial confirmed a robust safety profile with no serious adverse events reported.

No.	ID	Design	Qualitative Synthesis (Yes/No)	Quantitative Synthesis (Yes/No)	Support
**36**	Samami et al., 2021, Iran	Randomized Controlled Trials	Yes	Yes	The study met all eligibility criteria. **Population:** adult women diagnosed with stages I to III breast cancer, undergoing chemotherapy, with no psychiatric comorbidities or recent traumatic life events. **Intervention:** a six-week supportive psychosocial program, consisting of educational modules on breast cancer, diaphragmatic breathing, progressive muscle relaxation, emotion- and problem-focused coping strategies, stress management, social support, and spiritual coping, delivered in weekly 90-minute sessions by a trained facilitator. **Comparison**: a usual care control group receiving standard oncological information from healthcare staff without any structured psychological intervention. **Outcomes:** primary outcomes included coping strategies—problem-focused and emotion-focused—measured using the Ways of Coping Questionnaire; secondary outcome was stress, measured via the Depression-Anxiety-Stress Scale (short version). The study showed statistically and clinically significant increases in adaptive coping and reductions in maladaptive coping and stress levels, maintained one month post-intervention. A randomized controlled design and effect size calculations supported the study's inclusion in meta-analytic synthesis.
**37**	Santoyo-Olsson et al., 2022, USA	Randomized Controlled Trials	Yes	Yes	The study met all eligibility criteria. **Population**: Spanish-speaking Latina women newly diagnosed with nonmetastatic breast cancer residing in Northern California, USA. **Intervention**: A peer-delivered, cognitive behavioral stress management intervention (Nuevo Amanecer), delivered over eight weeks. **Comparison:** Baseline data from a randomized controlled trial with standard care as control (although the support statement refers to baseline data only). **Outcomes:** Psychological distress outcomes measured were health-related distress and general anxiety. These were assessed in relation to multilevel coping resources—specifically intrapersonal (self-efficacy, coping confidence), interpersonal (perceived neighborhood cohesion), and organizational/community resources (healthcare engagement). The study found that greater self-efficacy and coping confidence were significantly associated with lower health distress and anxiety. Perceived neighborhood cohesion was also inversely associated with health distress. No organizational/community level resources were significantly associated with outcomes.

No.	ID	Design	Qualitative Synthesis (Yes/No)	Quantitative Synthesis (Yes/No)	Support
**38**	Shergill et al., 2022, Canada	Randomized Controlled Trials	Yes	Yes	The study met all eligibility criteria. **Population**: adult women who were breast cancer survivors experiencing chronic neuropathic pain for more than six months, with a confirmed diagnosis and a minimum baseline pain score of 4 on a validated inventory. **Intervention**: an eight-week group-based mindfulness-based stress reduction program tailored for breast cancer survivors, which included weekly 2.5-hour sessions, a retreat, and was facilitated by trained professionals with mindfulness-based intervention credentials. **Comparison**: a waitlist control group that received usual care and later had the option to participate in the mindfulness-based program. **Outcomes:** primary outcome was pain interference, measured using the Brief Pain Inventory; secondary outcomes included measures of depressive symptoms, pain catastrophizing, emotional function, mindfulness, perceived stress, and quality of life using multiple validated instruments. Despite rigorous implementation, no statistically significant improvements were observed in the primary or secondary outcomes at the 3-month follow-up. The study emphasized the need to report non-significant results to mitigate publication bias and to refine outcome measures and timing in future research.

No.	ID	Design	Qualitative Synthesis (Yes/No)	Quantitative Synthesis (Yes/No)	Support
**39**	Tutino et al., 2022, USA	Randomized Controlled Trials	Yes	Yes	The study met all eligibility criteria. **Population:** older adults (aged 70 years and older) diagnosed with breast, prostate, lung, gynecologic cancer, or lymphoma, either currently undergoing active treatment or within six months post-treatment, who also exhibited psychological distress (defined by thresholds on the Distress Thermometer or the Hospital Anxiety and Depression Scale), and demonstrated sufficient cognitive and functional status (Karnofsky Performance Status ≥60 and Blessed Orientation Memory Concentration score ≤11). **Intervention**: the Cancer and Aging: Reflections for Elders-Expressive Writing (CARE-Express) intervention, a structured expressive writing program delivered via telephone over five sessions across seven weeks, designed to improve coping and psychological well-being by adapting principles from the original Cancer and Aging: Reflections for Elders psychotherapy intervention. **Comparison**: a historical control group receiving Enhanced Social Work Control, consisting of five telephone sessions over the same period, led by geriatric social workers providing supportive psychotherapy. **Outcomes:** the intervention demonstrated feasibility with high retention (90%) and treatment fidelity (97%). Preliminary findings indicated reductions in depression (Cohen's d = 0.69, p = 0.01) and demoralization (d = 0.50, p = 0.06), as well as improved spiritual well-being and reduced behavioral disengagement in the intervention group. Effects attenuated over time but remained favorable for the expressive writing group at four-month follow-up.

No.	ID	Design	Qualitative Synthesis (Yes/No)	Quantitative Synthesis (Yes/No)	Support
**40**	Victorson et al., 2020, USA	Randomized Controlled Trials	Yes	Yes	The study met all eligibility criteria. **Population:** Young adults aged 18–39 diagnosed with any type of cancer, predominantly female and Caucasian, with a variety of cancer types including breast cancer and lymphoma. **Intervention:** An eight-week standardized in-person mindfulness-based stress reduction program, including weekly 2.5-hour sessions and a half-day retreat, conducted by trained instructors with fidelity monitoring through recordings and supervision. **Comparison**: A waitlist control group that received the same assessments but did not participate in the intervention until after the 16-week assessment period. **Outcomes:** Measures included a range of validated instruments assessing physical symptoms (fatigue, sleep disturbance, pain), psychosocial distress (anxiety, depression, social isolation), and overall wellbeing (self-kindness, emotional support, mindfulness, post-traumatic growth). The intervention was found to be feasible and acceptable with over 70% of participants endorsing its utility. Statistically significant improvements were observed in sleep disturbance, intolerance of uncertainty, post-traumatic growth, and self-kindness within the intervention group.
**41**	Winger et al., 2023, USA	Randomized Controlled Trials	Yes	Yes	The study met all eligibility criteria. **Population**: adults diagnosed with stage IV solid tumor cancers and experiencing moderate to severe pain. **Intervention:** a structured psychosocial intervention termed Meaning-Centered Pain Coping Skills Training, delivered in four weekly 60-minute individual sessions via telehealth by trained therapists following a manualized protocol, integrating cognitive-behavioral strategies and meaning-centered therapeutic approaches. **Comparison**: a usual care group receiving standard oncological pain management without additional psychological intervention. **Outcomes**: self-reported levels of pain severity, pain interference, self-efficacy in pain management, and spiritual well-being (specifically dimensions of meaning and peace), as well as psychological distress indicators including anxiety, depression, and hopelessness, measured at baseline, 5-week, and 10-week follow-ups. The trial reported high feasibility, strong retention, and moderate-to-large effect sizes in primary outcomes, indicating promise for the intervention's clinical application.

No.	ID	Design	Qualitative Synthesis (Yes/No)	Quantitative Synthesis (Yes/No)	Support
**42**	Wittmann et al., 2022, USA	Randomized Controlled Trials	Yes	Yes	The study met all eligibility criteria. **Population**: Adult couples in which one partner was diagnosed with localized prostate cancer and scheduled to undergo treatment (surgery, radiation therapy, or combined radiation and hormonal therapy), with eligibility extended to all sexual orientations. **Intervention:** The TrueNTH Sexual Recovery Intervention—an interactive, web-based psychosexual program tailored to treatment type and sexual orientation, comprising six modules over a seven-month period. It focused on preparing for and coping with treatment-related sexual and emotional changes, introducing sexual aids, and encouraging recovery of sexual intimacy through structured activities. **Comparison**: A control group receiving standard information via the American Cancer Society's webpage on sexuality after cancer. **Outcomes:** Primary outcome was satisfaction with sex life at six months post-treatment, measured by the Patient-Reported Outcomes Measurement Information System Global Satisfaction With Sex Life scale. Secondary outcomes included sexual interest, sexual activity, use of sexual aids, and quality of life measured at three and six months. While no significant difference was observed in sexual satisfaction at six months, couples in the intervention group engaged more frequently in sexual activity, particularly nonpenetrative acts, at the three-month follow-up, and reported higher overall satisfaction with the intervention. The randomized controlled design across six sites and high user satisfaction support the intervention's acceptability and its potential as a psychosexual support tool in survivorship care.

References: All studies referenced herein are fully cited within the main body of the manuscript.

Table S3. Excluded Studies from Qualitative and Quantitative Synthesis with Rationale Based on Eligibility Criteria.

No.	Authors	Design	Eligibility	Support
**1**	Badr et al., 2019, USA	Randomized controlled trial	Excluded	It utilized a dyadic intervention model focusing on couples, with psychological outcomes assessed at the pair level. Although the study was a randomized controlled trial, the intervention targeted both patients and caregivers simultaneously, making it impossible to isolate patient-level psychological effects. This design did not meet the inclusion criteria requiring individually focused psychological interventions with patient-specific outcome reporting.
**2**	Bidstrup et al., 2023, Denmark	Randomized comparative effectiveness trial	Excluded	There was no control group without a psychological component. Participants in the standard care arm received municipal rehabilitation services that included psychosocial support and nurse-led guidance during oncology consultations. The intervention arm added structured nurse navigation and symptom screening but did not test a distinct psychological intervention against a neutral or non-psychological control. As such, the study does not meet the criteria for inclusion as a randomized controlled trial evaluating an independent, standardized psychological intervention.
**3**	Bisseling et al., 2017, Netherlands	Observational (Prospective mixed-methods cohort study)	Excluded	There was no control group. No randomization was performed. Participants self-selected and all received the same MBSR intervention. It is a prospective interventional cohort without a parallel comparison, and therefore does not meet the criteria for an RCT (Randomized Controlled Trial).
**4**	Chan et al. (2015), Malaysia	Observational (Prospective Cohort)	Excluded	There was no psychological intervention, no randomization, and no control group. The study is an observational prospective cohort that examined the effects of psychological morbidity (anxiety and depression) on cancer mortality using clinical diagnostic interviews and self-reported distress levels. Therefore, it does not meet the eligibility criteria for inclusion as a randomized controlled trial.
**5**	Compen et al. (2015), Netherlands	Study Protocol for a Multicenter Randomized Controlled Trial	Excluded	The study is a protocol for a randomized controlled trial and does not report any empirical results. No data on psychological outcomes are presented, thus it does not meet the inclusion criteria for meta-analysis, which require reported intervention outcomes. It is a study protocol only.

No.	Authors	Design	Eligibility	Support
**6**	Galvão et al., 2021_Australia	Randomized Controlled Trial	Excluded	The study was excluded from both analyses. Although it employed a randomized controlled trial design and included psychological distress as a secondary outcome, the population was limited exclusively to men with prostate cancer undergoing androgen deprivation therapy. This target population does not align with the inclusion criteria, which required mixed or broader oncological populations inclusive of female participants or other cancer types. Additionally, the intervention tested various modes of exercise (impact-loading, resistance, and aerobic), not a psychological intervention. Thus, the study does not meet the eligibility criteria regarding population and intervention focus.
**7**	Zerbinati et al., 2024, Italy	Observational (multicenter, cross-sectional study)	Excluded	There was no randomization: participants were not randomly assigned to an experimental or control group. There is no control group or comparative intervention. The study is cross-sectional and correlational, relying on validated questionnaires administered to oncology patients across multiple centers. The authors conducted regression and moderation analyses (e.g., examining the relationship between coping, informational support, and quality of life), but these do not involve an experimental intervention.
**8**	Langford et al., 2017, USA	Observational cross-sectional correlational)	Excluded	There was no random allocation of participants into an experimental or control group.<br>There is no active intervention (psychotherapeutic or otherwise).This is an observational, descriptive, and analytical study, which:<br>• Examines statistical relationships between life stress, coping, and cancer-related distress.<br>• Uses structural equation modeling (SEM), not interventions.
**9**	Schuurhuizen et al., 2015, Netherlands	Study protocol	Excluded	Study protocol only; no outcome data reported. Results not available for meta-analysis.
**10**	Huang et al., 2016, China	Study protocol	Excluded	Study protocol only; no outcome data reported. Results not available for meta-analysis.
**11**	Zhang et al., 2022, Malaysia	Study protocol	Excluded	This publication represents a study protocol outlining the planned methodology of a randomized controlled trial. No outcome data or post-intervention results are reported; therefore, it is not eligible for inclusion in the meta-analysis.

No.	Authors	Design	Eligibility	Support
**12**	Kwiatkowski et al., 2016, France	Study protocol	Excluded	This publication is a study protocol describing the design and planned methodology of a randomized controlled trial. No outcome data are reported, and therefore the study is not eligible for inclusion in the meta-analysis.

References: All studies referenced herein are fully cited within the main body of the manuscript.

Table S4. Synthesis of Included Studies by Intervention Type: Summary of Key Characteristics.

No.	Authors	Design	Population	Intervention	Comparison	Outcome	Methods	Conclusion
				**INTERVENTION: Structured Psychotherapeutic (evidence-based)**<br>Cognitive Behavioral Therapy (CBT); Acceptance and Commitment Therapy (ACT); Interpersonal Therapy (IPT); Supportive-existential or psychodynamic therapies (e.g., CALM)				
**1**	Breitbart et al., 2018, USA	Randomized Controlled Trials	Total: 321 patients; Individual Meaning-Centered Psychotherapy group: 109; Supportive Psychotherapy group: 108; Enhanced Usual Care group: 104.	Individual Meaning-Centered Psychotherapy – a structured, manualized intervention based on Viktor Frankl's logotherapy, designed to enhance spiritual well-being and sense of meaning. Category: Structured Psychotherapeutic Interventions.	Individual Meaning-Centered Psychotherapy versus Supportive Psychotherapy; Individual Meaning-Centered Psychotherapy versus Enhanced Usual Care; Supportive Psychotherapy versus Enhanced Usual Care.	**Primary outcomes (continuous):**<br>Spiritual Well-Being (Functional Assessment of Chronic Illness Therapy – Spiritual Well-being, FACIT-Sp):<br>IMCP: Pre = 28.48 (SD = 10.4), Mid = 31.26 (10.6), Post = 34.59 (9.4), Follow-up = 33.29 (9.3)<br>SP: Pre = 28.18 (9.3), Mid = 31.27 (8.2), Post = 32.86 (8.9), Follow-up = 32.28 (8.3)<br>EUC: Pre = 29.53 (8.0), Mid = 31.19 (8.4), Post = 31.88 (8.3), Follow-up = 33.25 (8.2)<br>**Other outcomes:**<br>Sense of Meaning (Life Attitude Profile – Revised, LAP-R)<br>Quality of Life (McGill Quality of Life Questionnaire, MQOL)<br>Anxiety (Hospital Anxiety and Depression Scale – Anxiety, HADS-A)<br>Depression (Hospital Anxiety and Depression Scale –	Stratified randomization; mixed-effects models; intent-to-treat and completers analysis (three or more sessions); False Discovery Rate correction; manualized treatment protocols; therapist adherence monitored through session recordings.	Individual Meaning-Centered Psychotherapy significantly improved spiritual well-being, sense of meaning, and quality of life compared to both Supportive Psychotherapy and Enhanced Usual Care. Effects were small to moderate. The intervention was especially effective for existential and spiritual distress in patients with advanced cancer. No significant advantage was observed for Supportive Psychotherapy compared to Enhanced Usual Care.

No.	Authors	Design	Population	Intervention	Comparison	Outcome	Methods	Conclusion
						Depression, HADS-D)<br>Hopelessness (Hopelessness Assessment in Illness, HAI)<br>Desire for Hastened Death (Schedule of Attitudes toward Hastened Death, SAHD)		
**2**	Fauser et al., 2023, Germany	Randomized Controlled Trials	Total: 160 breast cancer patients; Structured Short-Term Psychotherapy group: 80; Non-Specific Group Discussion group: 80.	Structured Short-Term Psychotherapy – a manualized, short-duration group intervention involving mindfulness meditation and behavioral therapy techniques such as acceptance and self-commitment. Category: Structured Psychotherapeutic Interventions.	Structured Short-Term Psychotherapy versus Non-Specific Group Discussion.	**Primary outcome (continuous):** Anxiety (Hospital Anxiety and Depression Scale – Anxiety, HADS-A):<br>• End of rehabilitation: Difference = -0.2; 95% CI = -1.2 to 0.7; p = 0.618.<br>• 3-month follow-up: Difference = 0.2; 95% CI = -0.9 to 1.3; p = 0.748.<br>**Secondary outcomes:** Depression (Hospital Anxiety and Depression Scale – Depression, HADS-D):<br>• Subgroup with high baseline anxiety: Difference = -1.9; 95% CI = -3.5 to -0.3; p = 0.019<br>Other outcomes (e.g., Distress, Fatigue, EORTC QLQ-C30 domains): No significant group differences reported.	Randomization using computer-generated numbers; analysis via linear regression models; adjustment for baseline values; subgroup analyses; imputation of missing data via chained equations; complete-case analysis and power analysis for subgroups.	There was no significant overall difference between structured short-term psychotherapy and non-specific group discussion. However, patients with high baseline anxiety benefited from the structured intervention by showing reduced depressive symptoms at the end of rehabilitation. These findings support targeted interventions for high-risk patients.
**3**	Graham et al., 2024, UK	Randomized Controlled Trials	Total: 79 women with early-stage breast cancer prescribed	Acceptance and Commitment Therapy (ACT) – a manualized, co-	Usual Care plus ACT versus Usual Care.	**Primary outcome (continuous):** Adherence (Adherence Starts with Knowledge Questionnaire, ASK-12):	Randomization stratified by site, route, and age; ACT delivered	The ACTION intervention was found to be acceptable, feasible, and delivered with high

No.	Authors	Design	Population	Intervention	Comparison	Outcome	Methods	Conclusion
			adjuvant endocrine therapy (AET); ACTION group: 40; Usual Care group: 39.	designed intervention comprising one individual ACT session and three group-based ACT sessions, supplemented with a digital platform. Category: Structured Psychotherapeutic Interventions.		• 3 months: UC = 21.9 (95% CI: 20.3–23.4), ACTION = 21.7 (20.2–23.3); Difference = −0.2 (−2.3, 2.0)<br>• 6 months: UC = 22.4 (21.1–23.7), ACTION = 21.4 (20.0–22.8); Difference = −1.0 (−2.9, 0.9)<br>**Secondary outcomes (continuous):**<br>• Psychological distress (Generalised Anxiety Disorder Questionnaire – GAD-7; Patient Health Questionnaire – PHQ-9)<br>• Quality of life (Work and Social Adjustment Scale – WSAS, Functional Assessment of Cancer Therapy – General and Endocrine Subscales [FACT-G, FACT-ES-19/23])<br>• Psychological flexibility (Valuing Questionnaire – VQ)<br>• Symptom interference (Hot Flash Related Daily Interference Scale – HFRDIS; Multidimensional Assessment of Fatigue – MAF; PROMIS Pain Interference; Day-to-Day Impact of Vaginal Ageing Questionnaire – DIVA)	by trained clinical psychologists; analysis via adjusted means and 95% confidence intervals; outcome measures collected at baseline, 3 and 6 months; procedural and ACT fidelity assessed with ACT Fidelity Measure (ACT-FM); descriptive statistics without formal hypothesis testing.	fidelity. Promising signals of effectiveness were observed for medication adherence, quality of life, psychological distress, and psychological flexibility. A definitive phase III randomized controlled trial is warranted.
**4**	Gudenkauf et al., 2015, USA	Randomized Controlled Trials	Total: 183 women with stage 0–III	Cognitive-Behavioral Training (CBT) and	CBT vs. Health Education; RT vs. Health	**Primary outcomes (continuous):**<br>• Depressive Affect (Affects	3 × 2 repeated-measures ANOVA;	Both Cognitive-Behavioral Training and Relaxation Training

No.	Authors	Design	Population	Intervention	Comparison	Outcome	Methods	Conclusion
			breast cancer; Cognitive-Behavioral Training group: 55; Relaxation Training group: 70; Health Education group: 58.	Relaxation Training (RT) – two brief, manualized, 5-week group interventions modeled after Cognitive-Behavioral Stress Management. Category: Structured Psychotherapeutic Interventions.	Education; CBT vs. RT.	Balance Scale – ABS):<br>CBT vs. HE: F(1,131) = 10.51, p = .002, d = 0.69; RT vs. HE: F(1,131) = 4.91, p = .028, d = 0.48<br>• Cancer-Specific Distress (Impact of Event Scale – Intrusion Subscale, IES-I):<br>CBT vs. HE: F(1,131) = 8.65, p = .004, d = 0.62; CBT vs. RT: F(1,131) = 11.85, p < .001, d = 0.78<br>• Social Disruption (Sickness Impact Profile – Social Interaction, SIP-SI):<br>RT vs. HE: F(1,131) = 4.39, p = .038, d = 0.50<br>• Emotional Well-Being (Functional Assessment of Cancer Therapy – Emotional Well-Being, FACT-EWB):<br>CBT vs. HE: F(1,131) = 6.98, p = .009, d = 0.52; RT vs. HE: F(1,131) = 0.08, p = .777<br>**Stress Management Resources (continuous):**<br>• Interpersonal Support (Social Provisions Scale – Reliable Alliance, SPS-RA):<br>CBT vs. HE: F(1,130) = 8.32, p = .005, d = 0.61<br>• Perceived Relaxation Skills (Measure of Current Status –	analysis controlled for stage, time since surgery, and income; simple effects and pairwise interaction contrasts with Cohen's d; measures collected at baseline and post-intervention; randomization with concealed allocation; supervised fidelity checks for all conditions.	interventions led to significant improvements in psychological adaptation and stress management skills compared to an attention-matched Health Education control. CBT improved depressive affect, emotional well-being, and cancer-specific distress, while RT improved social disruption and perceived relaxation abilities. Findings support the use of brief, manualized group interventions post-surgery in breast cancer care.

No.	Authors	Design	Population	Intervention	Comparison	Outcome	Methods	Conclusion
						Relaxation Subscale, MOCS): RT vs. HE: F(1,131) = 20.81, p < .001, d = 0.92; CBT vs. HE: F(1,131) = 3.29, p = .072, d = 0.42		
**5**	Han et al., 2021, China	Randomized Controlled Trials	Total: 130 patients with advanced cancer; Combined Naikan and Morita Therapy group: 65; Control group (standard medical care): 65.	Combined Naikan Therapy and Morita Therapy – structured Eastern-origin psychotherapies focusing on introspection, acceptance, and adaptive behavior. Category: Coping and Psychological Resilience Interventions.	Combined Naikan and Morita Therapy versus standard medical care.	**Primary outcomes (continuous):**<br>• Psychological distress (Distress Thermometer):<br>Pretreatment – Treatment group: M = 4.62 (SD = 1.36), Control group: M = 4.91 (SD = 1.38)<br>Posttreatment – Treatment: M = 2.42 (SD = 1.03), Control: M = 4.81 (SD = 1.43); ANCOVA P < .001<br>• Posttraumatic growth (Posttraumatic Growth Inventory, PTGI – Total Score):<br>Pretreatment – Treatment: Median = 43, Control: Median = 41<br>Posttreatment – Treatment: Median = 74, Control: Median = 45; ANCOVA P < .001<br>**Subscales (all P < .001):**<br>Relating to others, New possibilities, Personal strength, Spiritual changes, Appreciation of life<br>**Secondary outcomes:**	Block randomization; ANCOVA and Wilcoxon tests; intention-to-treat; sample size based on meta-analytic effect size; adherence monitored; outcome assessors blinded; SPSS (Statistical Package for the Social Sciences) and SAS (Statistical Analysis System) for analysis.	Combined Naikan and Morita Therapies significantly reduced psychological distress and enhanced posttraumatic growth in advanced cancer patients. Improvements were observed across all subscales of growth and specific symptoms. The therapies offer a culturally congruent, effective palliative psychological intervention.

No.	Authors	Design	Population	Intervention	Comparison	Outcome	Methods	Conclusion
						Symptom checklist: Significant reductions in fear (p = .023), insomnia (p < .001), anxiety (p = .034), and appetite loss (p = .002).		
**6**	Huang et al., 2024, China	Randomized Controlled Trials	Total: 139 patients with esophageal or gastric cancer; Behavioral Activation (BA) + Care As Usual (CAU) group: 70; CAU group: 69.	Behavioral Activation (BA) – a brief, structured psychosocial intervention promoting meaningful activity engagement and reducing avoidance patterns, based on the Behavioral Activation for Depression framework. Category: Structured Psychotherapeutic Interventions.	BA + CAU versus CAU.	**Primary outcome (continuous):**<br>• Psychological distress (Psychological Distress Thermometer – DT):<br>• T1: β = −1.857, 95% CI [−2.248, −1.467], SE = 0.199, p < 0.001<br>• T2: β = −3.786, 95% CI [−4.333, −3.238], SE = 0.279, p < 0.001<br>**Secondary outcomes (continuous):**<br>• Anxiety (Generalized Anxiety Disorder 7-item scale – GAD-7):<br>• T1: β = −3.456, 95% CI [−4.342, −2.571], SE = 0.452, p < 0.001<br>• T2: β = −6.339, 95% CI [−7.559, −5.119], SE = 0.623, p < 0.001<br>• Self-efficacy (General Self-Efficacy Scale – GSES):<br>• T1: β = 0.366, 95% CI [0.256, 0.475], SE = 0.056, p < 0.001<br>• T2: β = 0.749, 95% CI [0.609, 0.889], SE = 0.072, p < 0.001<br>• Activation (Behavioral Activation for Depression Scale – Activation Subscale – BADS-A):	Single-blind randomization with concealed allocation; GEE models for repeated measures; mediation analysis with PROCESS macro; bootstrapping; adjustment for socio-demographic and clinical covariates; intention-to-treat principle; use of validated psychological scales.	Behavioral Activation significantly reduced psychological distress and anxiety, and increased self-efficacy and activation levels in cancer patients. The mediating role of self-efficacy between activation and psychological distress was confirmed. The intervention proved suitable for remote application and delivery by non-specialists, suggesting broad clinical and public health utility.

No.	Authors	Design	Population	Intervention	Comparison	Outcome	Methods	Conclusion
						• T1: β = 6.770, 95% CI [5.552, 7.989], SE = 0.622, p < 0.001<br>• T2: β = 12.522, 95% CI [10.565, 14.479], SE = 0.999, p < 0.001		
**7**	Isaka et al., 2021, Japan	Randomized Controlled Trials	Total: 972 women aged 20–69 undergoing cervical cancer screening; Intervention (leaflet) group: 493; Control group: 479.	Information Leaflet – a printed educational tool explaining screening process, positive result implications, and next steps, designed to reduce psychological distress. Category: Coping and Psychological Resilience Interventions.	Information Leaflet versus no leaflet (standard procedure).	**Primary outcome (dichotomous):**<br>• Psychological distress (Cancer Worry Scale, score ≥ 15):<br>Positive result recipients – Intervention: 60%, Control: 79%; Odds Ratio (OR) = 2.57, 95% Confidence Interval (CI) [1.87–3.54]<br>**Secondary outcome (dichotomous):**<br>• Intention to undergo further examination:<br>Positive result recipients – Intervention: 95%, Control: 97%	Random assignment via distributed questionnaires; outcome assessed via validated scales (Cancer Worry Scale, K6); analysis stratified by cytological grade; intention-to-treat analysis; ORs and 95% CIs estimated for group comparisons.	Providing educational information via leaflet reduced psychological distress without decreasing intention to seek further examination among women receiving a positive cervical cancer screening result. A low-cost, scalable communication tool with clinical and policy relevance.
**8**	Li et al., 2023, China	Randomized Controlled Trials	Total: 143 adolescent and young adult cancer patients; Physical Activity (PA) group: 47; Behavioral Activation (BA)	Physical Activity (PA) program using intelligent wearable devices; Behavioral Activation (BA) delivered via internet-based modules. Category:	PA vs. TAU; BA vs. TAU; PA vs. BA.	**Primary outcome (continuous):**<br>• Salivary cortisol: PA vs TAU – Cohen's d = 1.93 (T1), 0.54 (T2), p < 0.001 / 0.006; BA vs TAU – d = 1.71 (T1), 0.55 (T2)<br>Secondary outcomes (continuous):<br>• Psychological distress (Hospital Anxiety and	Linear Mixed Models (LMM); intention-to-treat; SPSS v24; comparisons using ANOVA, Kruskal-Wallis, Bonferroni-corrected post-	Both physical activity and behavioral activation significantly improved psychological distress markers (e.g., HADS, cortisol, IPAQ, GSES). The physical activity program showed greater and longer-lasting effects,

No.	Authors	Design	Population	Intervention	Comparison	Outcome	Methods	Conclusion
			group: 48; Treatment-as-usual (TAU): 48.	Coping and Psychological Resilience Interventions.		Depression Scale – HADS): Total score – PA vs TAU: $p < 0.001$, d = medium-large; Anxiety: $p < 0.001$; Depression: not significant<br>• Physical activity (International Physical Activity Questionnaire – IPAQ): Total PA – PA vs TAU: d = 1.47 (T1), 1.71 (T2), $p < 0.001$; PA vs BA: d = 0.53–0.79<br>• Social support (Social Support Rating Scale – SSRS): PA and BA groups: significant improvement ($p < 0.05$)<br>• Self-efficacy (General Self-Efficacy Scale – GSES): PA and BA groups: significant improvement ($p < 0.05$)<br>• Sleep quality (Pittsburgh Sleep Quality Index – PSQI): PA and BA groups: significant improvement ($p < 0.05$)	hoc tests; Cohen's d reported for effect sizes; validated scales: HADS, IPAQ, SSRS, GSES, PSQI; salivary biomarkers via ELISA; 3-point assessments (T0, T1, T2).	especially on physical activity and salivary cortisol. These interventions are feasible and beneficial for AYACPs, with physical activity proving superior in adherence and cost-effectiveness.
**9**	Lopez et al., 2023, USA	Randomized Controlled Trials	Total: 35 cancer patients with anxiety and/or depression; Meditation App group: 17; Waitlist control: 18.	Self-administered Meditation Application – mobile app allowing 5/10/15-minute guided meditation sessions. Category: Mindfulness and	Meditation App vs. Waitlist Control.	**Primary outcome (continuous):**<br>• Anxiety (pre-to-post session, 0–8 scale): $\Delta = -1.57$, 95% Confidence Interval (CI) [−2.45, −0.69]<br>**Secondary outcomes (continuous):**<br>• Fatigue (Edmonton Symptom Assessment Scale – ESAS): $\Delta =$	Minimization randomization; Wilcoxon and linear mixed models; effect sizes as Cohen's d and ε^2; SAS 9.4 software; feasibility	This pilot study demonstrated the feasibility and acceptability of a self-guided meditation App for cancer patients. While between-group effects on mood measures were limited, within-group

No.	Authors	Design	Population	Intervention	Comparison	Outcome	Methods	Conclusion
				Stress Reduction Intervention.		−1.5 (IQR = 2.5) vs. +0.5 (IQR = 2.0), p = .016, ε^2 = 0.19<br>• Depression (ESAS): p = .041 (within-group, Meditation)<br>• Anxiety (ESAS): p = .016 (within-group, Meditation)<br>• Appetite (ESAS): p = .031 (within-group, Meditation)<br>• Depression (Hospital Anxiety and Depression Scale – HADS): Δ = −0.69, Cohen's d = 0.11<br>• Anxiety (HADS): Δ = −1.33, Cohen's d = 0.27<br>• Sleep Quality (Pittsburgh Sleep Quality Index – PSQI): no significant changes	threshold: 70% adherence (≥2 sessions/week).	improvements in fatigue, depression, anxiety, and appetite were observed. App length influenced anxiety reduction, suggesting potential for dose-dependent effects.
**10**	Manne et al., 2017, USA	Randomized Controlled Trials	Total: 352 women newly diagnosed with gynecologic cancer; CCI: 118; SC: 118; UC: 116.	Coping and Communication-enhancing Intervention (CCI) – structured, 8-session cognitive-behavioral intervention focused on coping skills and communication. Category: Structured Psychotherapeutic Intervention.	CCI vs. Supportive Counseling (SC) vs. Usual Care (UC).	**Primary outcome (continuous):**<br>• Depression (Beck Depression Inventory – BDI): CCI reduced mean scores by 5.86 pts from baseline to 6 months vs. SC and UC<br>• Cancer-specific distress (Impact of Events Scale – IES): CCI reduced scores by 7.87 pts over 6 months<br>**Secondary outcomes (continuous):**<br>• Emotional well-being (Functional Assessment of Cancer Therapy – Emotional	Multilevel modeling (MLM) using SPSS v22; covariates included age, ethnicity, marital status, education, cancer stage, baseline depression, physical symptoms; linear slopes	CCI significantly reduced depression and cancer-specific distress, and improved emotional well-being over 6 months, outperforming both SC and UC. SC showed no significant benefits compared to UC. CCI is an effective structured intervention suitable for integration into standard oncological care. No long-term effects detected beyond 6 months.

No.	Authors	Design	Population	Intervention	Comparison	Outcome	Methods	Conclusion
						subscale): CCI improved scores by 2.28 pts<br>• Fear of recurrence (Concerns about Recurrence Scale): No significant effect	and random intercepts modeled; separate ANCOVAs per time-point; Tukey post-hoc comparisons; treatment fidelity assessed via session recordings.	
**11**	Marziliano et al., 2023, USA	Randomized Controlled Trials	Total: 617 women with early-stage breast cancer; Final sample with outcome data at 2 months: 388 (Intervention = 197; Control = 191)	Healing Choices – a multimedia interactive decision aid based on self-regulation theory, incorporating videos, education modules, and value clarification tools. Category: Coping and Psychological Resilience Interventions.	Healing Choices + standard care vs. standard care alone (NCI print materials).	**Primary outcome (continuous):**<br>• Psychological distress (Impact of Events Scale – Intrusion Subscale):<br>– Intervention: M = 16.09, SD = 10.25; Control: M = 14.37, SD = 8.73;<br>– B = 1.88, 95% Confidence Interval (CI) [−0.03, 3.80], t(383) = 1.94, p = .05<br>**Secondary outcome (continuous):**<br>• Decisional conflict (Decisional Conflict Scale – DCS):<br>– Total score: Intervention = 38.44 ± 13.09; Control = 38.79 ± 13.88; B = −0.06, 95% CI [−2.74, 2.63], p = ns<br>– Subscales (all p = ns):	SPSS v22 and SAS v9.4; t-tests, chi-square, linear and logistic regression; intent-to-treat and as-treated analysis; self-reported program usage; validated scales: DCS and IES.	Healing Choices did not significantly reduce decisional conflict and was associated with slightly increased psychological distress in intention-to-treat analysis. However, among actual users, the intervention significantly improved decisional support without increasing distress. Low engagement suggests future focus on usability and tailoring to avoid information overload.

No.	Authors	Design	Population	Intervention	Comparison	Outcome	Methods	Conclusion
						Uncertainty, Informed, Value Clarity, Decisional Support, Effective Decision<br>**As-treated analysis (program users vs non-users):**<br>• Decisional Support: B = −4.31 (SE = 2.09), p = .04<br>• Psychological distress: no significant difference		
**12**	Nissen et al., 2020, Denmark	Randomized Controlled Trials	Total: 150 breast and prostate cancer survivors (Intervention = 104; Control = 46); mean age = 54.55 (breast), 64.94 (prostate)	Internet-delivered Mindfulness-Based Cognitive Therapy (iMBCT) – 8-week online program with therapist feedback and cancer-specific content. Category: Mindfulness and Stress Reduction Intervention.	iMBCT vs. care-as-usual (waitlist control).	**Primary outcome (continuous):**<br>• Anxiety (State-Trait Anxiety Inventory – STAI-Y): d = 0.45, p = .017 (post); d = 0.40, p = .029 (6-month follow-up)<br>• Depression (Beck Depression Inventory – BDI-II): d = 0.42, p = .024 (post); d = 0.28, p = .131 (follow-up)<br>**Secondary outcomes (continuous):**<br>• Well-being (WHO-5): d = 0.48, p = .010 (6-month follow-up only)<br>• Perceived stress (Perceived Stress Scale – PSS-10) and insomnia (Insomnia Severity Index – ISI): no significant effects	Two-level multilevel modeling (MLM); intention-to-treat; SPSS v25; missing data sensitivity analysis via Zero-Effect from Baseline (ZEFB); model fit (linear, log-linear, quadratic); effect sizes from Group × Time F-tests; subgroup analyses by cancer type.	iMBCT significantly reduced anxiety and depression at post-intervention and anxiety and well-being at 6 months. No effects on stress or sleep. Despite high attrition, especially in the intervention group, adherence was high among completers. iMBCT appears effective and feasible, especially for anxiety, but selection and adherence remain challenges for broader implementation.
**13**	Park et al., 2020, Japan	Randomized Controlled Trials	Total: 74 women with Stage I–III breast cancer;	Mindfulness-Based Cognitive Therapy (MBCT) – 8-week	MBCT vs. wait-list control.	**Primary outcome (continuous):**<br>• Psychological distress (Hospital Anxiety and	Repeated-measures linear mixed-effects	MBCT significantly reduced psychological distress, anxiety,

No.	Authors	Design	Population	Intervention	Comparison	Outcome	Methods	Conclusion
			MBCT = 38; Wait-list control = 36.	group program with cognitive therapy-based psychoeducation and meditation practices tailored to breast cancer. Category: Mindfulness and Stress Reduction Intervention.		Depression Scale – HADS):<br>– T1: Δ = −7.82, 95% CI [−11.28, −6.35], $p < 0.001$, Cohen's d = 1.17<br>– T2: Δ = −6.56, 95% CI [−10.09, −5.22], $p < 0.001$, d = 1.03<br>**Secondary outcomes (continuous):**<br>• Anxiety (HADS-A): T1: Δ = −3.40, d = 1.01; T2: Δ = −2.81, d = 1.06, $p < 0.001$<br>• Depression (HADS-D): T1: Δ = −4.63, d = 1.22; T2: Δ = −3.75, d = 1.28, $p < 0.001$<br>• Fear of cancer recurrence (Concerns About Recurrence Scale – CARS): T1: Δ = −2.16, d = 0.43; T2: Δ = −2.74, d = 0.59<br>• Fatigue (Brief Fatigue Inventory – BFI): T1: Δ = −1.57, d = 0.66; T2: Δ = −1.23, d = 0.58<br>• Spiritual well-being (Functional Assessment of Chronic Illness Therapy – Spiritual – FACIT-Sp): T1: Δ = +8.09, d = 0.91; T2: Δ = +7.68, d = 0.82<br>• Quality of life (Functional Assessment of Cancer Therapy – General – FACT-G): T1: Δ = +13.35, d = 0.79; T2: Δ = +13.51, d	model; intention-to-treat; SPSS v25; Cohen's d for effect sizes; block randomization with anxiety stratification; group adherence evaluated via attendance and homework logs; mean intervention adherence: 6.76/8 sessions, 24.2 min/day home practice.	depression, fear of recurrence, and fatigue, and improved quality of life, spiritual well-being, and mindfulness skills. Effects were large and sustained at 4-week follow-up. This culturally adapted MBCT is effective for a wide range of symptoms and may be integrated into standard psycho-oncological care for Japanese breast cancer patients.

No.	Authors	Design	Population	Intervention	Comparison	Outcome	Methods	Conclusion
						= 0.78<br>• Mindfulness skills (Five Facet Mindfulness Questionnaire – FFMQ): T1: Δ = +13.2, d = 0.82; T2: Δ = +17.84, d = 1.09		
**14**	Rodin et al., 2018, Canada	Randomized Controlled Trials	Total: 305 patients with advanced cancer; CALM = 151; Usual Care = 154.	Managing Cancer and Living Meaningfully (CALM) – a brief, manualized supportive-existential psychotherapy addressing symptom management, self-concept, spiritual well-being, and end-of-life issues. Category: Structured Psychotherapeutic Intervention.	CALM plus usual care vs. usual care alone.	**Primary outcome (continuous):**<br>• Depression (Patient Health Questionnaire-9 – PHQ-9):<br>– t1: M = 7.01 (UC) vs. 5.97 (CALM); Δ = 1.09, p = .04, Cohen's d = 0.23<br>– t2: M = 6.64 (UC) vs. 5.35 (CALM); Δ = 1.29, p = .02, d = 0.29<br>– Remission at t2 (PHQ-9 < 8): UC = 37%, CALM = 66%, OR = 3.29, p = .005<br>**Secondary outcomes (continuous):**<br>• End-of-life preparation (QUAL-EC): Δ = 1.20 at t2, p = .001<br>• Anxiety (GAD-7), Death anxiety (DADDS), Demoralization (DS), Spiritual well-being (FACIT-Sp-12), etc. (details above)	Analysis of covariance; mixed-models; multiple imputation; SPSS and SAS v9.3; FDR corrections; intention-to-treat; subgroup analysis by death anxiety (DADDS). Outcomes assessed at baseline (t0), 3 months (t1), and 6 months (t2).	CALM significantly reduced depressive symptoms at 3 and 6 months and improved end-of-life preparation. Remission rates and clinically meaningful improvements were higher in the intervention group. Subgroup analysis showed enhanced benefits in patients with moderate death anxiety. CALM is a feasible, effective, and adaptable intervention for advanced cancer patients.
**15**	Ross et al., 2016, USA	Randomized Controlled Trials	Total: 29 patients with life-threatening cancer and	Single moderate-dose Psilocybin (0.3 mg/kg) in conjunction with	Psilocybin + psychotherapy vs. Niacin + psychotherapy	**Primary outcomes (continuous):**<br>• Anxiety and Depression (Hospital Anxiety and	Mixed-effect repeated measurement model	Psilocybin produced rapid, robust, and sustained reductions in cancer-related anxiety

No.	Authors	Design	Population	Intervention	Comparison	Outcome	Methods	Conclusion
			clinically significant anxiety/depression; Psilocybin-first group = 14; Niacin-first group = 15	targeted psychotherapy. Category: Structured Psychotherapeutic Intervention (pharmacological-psychosocial model).	(crossover after 7 weeks)	Depression Scale – HADS, Beck Depression Inventory – BDI, State-Trait Anxiety Inventory – STAI):<br>– Psilocybin group showed large effect size improvements: HADS-A d = 1.03, HADS-D d = 0.92, BDI d = 1.06, STAI-S d = 1.12, all $p < 0.01$.<br>– Significant sustained reductions at 6.5-month follow-up (60–80% maintained clinical response).<br>**Secondary outcomes:**<br>• Existential distress (Demoralization Scale – DEM, Hopelessness Assessment in Illness – HAI, Death Anxiety Scale – DAS, Death Transcendence Scale – DTS)<br>• Quality of life (WHO-Bref), Spiritual well-being (FACIT-Sp), Mystical Experience (MEQ 30)	(MMRM), SAS PROC MIXED; Cohen's d; bootstrap mediation (PROCESS macro); Spearman correlations; within/between-group t-tests; subgroup analyses.	and depression. Improvements in existential distress, spiritual well-being, and quality of life were also observed. The intensity of mystical experience mediated therapeutic outcomes. The intervention is safe and highly meaningful.
				**INTERVENTION: Mindfulness and Stress Reduction**<br>Mindfulness-Based Stress Reduction (MBSR); Mindfulness-Based Cognitive Therapy (MBCT); Relaxation therapy and guided meditation				
**16**	Bagherzadeh et al., 2022, Iran	Randomized Controlled Trials	Total: 46 women with breast cancer (Intervention = 22; Control = 24); mean age:	Mindfulness-Based Stress Reduction (MBSR) – eight 90-min sessions based on Kabat-Zinn protocol. Category:	MBSR vs. control group (no intervention; received pamphlet/CD post-study)	**Primary outcome (continuous):**<br>• Rumination (Rumination Questionnaire – Nolen-Hoeksema):<br>– Intervention: 43.18 ± 13.59 (pre), 40.36 ± 10.28 (post), 40.82 ±	SPSS v19; Shapiro–Wilk for normality; chi-square, Mann–Whitney U for	MBSR training was associated with stabilization of rumination, whereas the control group showed a significant increase.

No.	Authors	Design	Population	Intervention	Comparison	Outcome	Methods	Conclusion
			44.50 ± 7.72 (intervention), 49 ± 7.48 (control)	Mindfulness and Stress Reduction Intervention.		9.67 (follow-up); repeated-measures ANOVA: p = 0.302 (ns)<br>– Control: 42.96 ± 13.93 (pre), 45.63 ± 12.54 (post), 47.29 ± 12.58 (follow-up); ANOVA: p < 0.001<br>• ANCOVA (adjusting pretest): Between-group difference significant at post (p = .01) and follow-up (p < .001)	demographics; repeated-measures ANOVA and ANCOVA for outcomes; intention-to-treat and per-protocol overlap; effect of pretest controlled in ANCOVA.	Although within-group change was not significant in the intervention group, the between-group difference showed MBSR's protective effect. Continued practice and long-term follow-up recommended for sustainable outcomes.
**17**	Bower et al., 2015, USA	Randomized Controlled Trials	Total: 71 premenopausal women with early-stage breast cancer; MAPS = 39, Wait-list control = 32	Mindful Awareness Practices (MAPS) – six weekly 2-hour group sessions with mindfulness meditation and psychoeducation. Category: Mindfulness and Stress Reduction Intervention.	MAPS vs. wait-list control	**Primary outcomes (continuous):**<br>• Perceived stress (Perceived Stress Scale – PSS): Δ = −4.17 (post), p = .004, d = 0.67<br>• Depressive symptoms (Center for Epidemiologic Studies Depression Scale – CES-D): Δ = −4.5, p = .095, d = 0.54<br>**Secondary outcomes:**<br>• Fatigue (Fatigue Symptom Inventory – FSI): p = .007<br>• Sleep quality (Pittsburgh Sleep Quality Index – PSQI): p = .015<br>• Hot flashes (Breast Cancer Prevention Trial Checklist – BCPT): p = .015<br>• Positive affect (Positive and	Linear mixed-effects models (SAS 9.1, Stata 12); transcriptional profiling and promoter-based bioinformatics (TELiS); ELISA assays for IL-6, CRP, sTNF-RII; intention-to-treat; RNA markers and transcription factor activity analyses; exploratory	MAPS improved stress, fatigue, sleep, and inflammatory gene expression (NF-κB, GR activity). Effects on circulating markers were nonsignificant. Short-term psychological and genomic benefits were observed, but were not maintained at 3-month follow-up, except for reduced cancer-related distress. More support may be needed for long-term practice among younger survivors.

No.	Authors	Design	Population	Intervention	Comparison	Outcome	Methods	Conclusion
						Negative Affect Schedule – PANAS-PA): p = .033<br>• Peace and meaning (Functional Assessment of Chronic Illness Therapy – FACIT): p = .001<br>• Fear of recurrence (Quality of Life in Adult Cancer Survivors – QLACS): 3-month follow-up: p = .048<br>• Cancer-related intrusive thoughts (Impact of Events Scale – IES): p = .002	dose-response by practice level.	
**18**	Cillessen et al., 2018, Netherlands	Randomized Controlled Trials	Total N = 245 distressed cancer patients; MBCT = 120, eMBCT = 125. Inclusion: HADS ≥11. Cancer types: mixed; Gender: ~85% women.	Mindfulness-Based Cognitive Therapy (MBCT) and eMBCT (online format), both adapted for cancer patients (8-week program). Category: Mindfulness and Stress Reduction Intervention.	MBCT vs. eMBCT (individual online) – both compared to TAU initially; analysis focuses on MBCT vs. eMBCT after TAU patients crossed over.	**Primary outcome (continuous):**<br>• Psychological distress (Hospital Anxiety and Depression Scale – HADS): MBCT T3 = 12.6 (6.7); eMBCT T3 = 9.6 (6.4); p = .049, Cohen's d = 0.22<br>**Secondary outcomes (continuous):**<br>• Rumination (Rumination Response Questionnaire – RRQ): MBCT = 34.6 (5.8); eMBCT = 33.9 (6.1)<br>• Positive mental health (Mental Health Continuum – Short Form – MHC-SF): MBCT = 43.6 (13.7); eMBCT = 48.3 (12.2)<br>• QoL (Short Form Health	Linear mixed-effect models (SPSS v22); hierarchical linear regressions; residual change score analyses for working mechanisms; effect sizes (Cohen's d); imputation for missing data; significance threshold p < .05.	Both MBCT and eMBCT reduced psychological distress and improved mental health over 9 months. eMBCT slightly superior, especially for patients with low initial mindfulness skills and conscientiousness. Working mechanisms included improvements in mindfulness, reduced rumination, and less fear of cancer recurrence.

No.	Authors	Design	Population	Intervention	Comparison	Outcome	Methods	Conclusion
						Survey – SF-12, Mental Component): MBCT = 46.5 (11.5); eMBCT = 48.9 (10.6)<br>• Fear of Cancer Recurrence (FCRI), Mindfulness (FFMQ), and predictors/moderators also tested		
**19**	Duval et al., 2022, Canada	Randomized Controlled Trials	Total N = 60 breast cancer survivors with chronic neuropathic pain; Intervention = 30, Control = 30; age range: 33–71 years	Mindfulness-Based Stress Reduction (MBSR) – eight 2.5-hour sessions + 6-hour retreat; guided by psychologist/social worker with MBSR and chronic pain experience. Category: Mindfulness and Stress Reduction Intervention.	MBSR vs. waitlist control group	**Primary outcome (continuous):**<br>• Cognitive function – CNS Vital Signs (CNS-VS, objective): no significant group*time interaction (p = .197, .497)<br>• Perceived cognition – Functional Assessment of Cancer Therapy-Cognitive Function (FACT-Cog): no significant group*time interaction (p = .142, .702)<br>• Subjective memory – Prospective and Retrospective Memory Questionnaire (PRMQ):<br>– PRMQ Total: significant interaction at 2-week post (b = −5.11, SE = 2.03, p = .013); not maintained at 3 months (p = .232)<br>– Retrospective: significant at 2-week (p = .016), ns at 3-month<br>– Prospective: marginal 2-week (p = .058), not sustained (p = .207)	SPSS v27; Mixed-level models (autoregressive covariance, Satterthwaite approximation); covariates: age, education; intent-to-treat; raw and standardized cognitive scores; condition-coded regression; group*time interaction tested.	MBSR did not significantly affect overall objective or subjective cognitive outcomes. However, short-term reductions in subjective prospective and retrospective memory difficulties were observed, suggesting potential for MBSR in addressing memory-related cognitive complaints. Effects not sustained at 3-month follow-up. Booster sessions may be needed for longer-term impact.

No.	Authors	Design	Population	Intervention	Comparison	Outcome	Methods	Conclusion
**20**	Gu et al., 2024, China	Randomized Controlled Trials	Total N = 78 patients with cervical cancer; Intervention = 40, Control = 38; age range: 33–71 years	Mindfulness-Based Stress Reduction (MBSR) – 8-week online program with videos, breathing, body scan, yoga, sitting meditation, mindfulness walking. Category: Mindfulness and Stress Reduction Intervention.	MBSR vs. routine nursing (admission/discharge education, supportive care, no psychological intervention)	**Primary outcome (continuous):**<br>• Cancer-Related Fatigue (Cancer Fatigue Scale – CFS):<br>– MBSR: 37.70±2.70 → 31.25±1.59 → 31.00±3.40 → 31.25±2.52<br>– Control: 36.74±4.21 → 36.53±4.24 → 33.74±4.18 → 36.79±4.98<br>– $p < 0.001$; partial $\eta^2 = 0.582$<br>**Secondary outcomes:**<br>• Sense of Coherence (SOC-13): MBSR group significant improvement ($p < 0.001$)<br>• Coping styles (Medical Coping Modes Questionnaire – MCMQ):<br>– Facing ↑ ($p < 0.001$), Yielding ↓ ($p < 0.001$), Avoidance = ns<br>• Uncertainty in Illness (Medical Uncertainty in Illness Scale – MUIS): ↓ post, ↑ at 3/6 months<br>• Perceived Social Support (Multidimensional Scale of Perceived Social Support – MSPSS): no significant change	SPSS v26; repeated-measures ANOVA; Bonferroni post-hoc; independent sample t-tests; intent-to-treat; η^2 for effect size; online WeChat delivery; single-blind design.	MBSR significantly improved Cancer-Related Fatigue, Sense of Coherence, and adaptive coping (facing↑, yielding↓) with sustained effects up to 6 months. Uncertainty in illness reduced immediately post-intervention, but rebounded at follow-up. No significant impact on perceived social support. Findings support MBSR integration in post-treatment care for cervical cancer patients.
**21**	Johns et al., 2015, USA	Randomized Controlled Trials	Total N = 35 cancer survivors with clinically-significant cancer-related fatigue; MBSR =	Mindfulness-Based Stress Reduction (MBSR-CRF) – 7-week program adapted for cancer-related fatigue with	MBSR vs. wait-list control; controls received MBSR post-T3	**Primary outcome (continuous):**<br>• Fatigue interference (Fatigue Symptom Inventory – FSI):<br>– T2: $d = -1.43$, $p < 0.001$<br>– T3: $d = -1.34$, $p < 0.001$<br>**Secondary outcomes:**	ANCOVA adjusting for T1 scores and mindfulness subscale differences;	MBSR significantly reduced cancer-related fatigue interference and improved related outcomes (depression, vitality, sleep, disability,

No.	Authors	Design	Population	Intervention	Comparison	Outcome	Methods	Conclusion
			18, Wait-list = 17; mostly women (94%) and breast cancer (86%)	2-hour sessions; mindfulness meditation, yoga, self-regulatory techniques. Category: Mindfulness and Stress Reduction Intervention.		• Fatigue severity: T2 d = −1.55, T3 d = −1.54<br>• Fatigue days: T2 d = −1.08, T3 d = −1.22<br>• Percent day fatigued: T2 d = −1.83, T3 d = −1.73<br>• Vitality (SF-36): T2 d = 1.29, T3 d = 1.73<br>• Depression (PHQ-8): T2 d = −1.30, T3 d = −1.71<br>• Sleep disturbance (Insomnia Severity Index – ISI): T2 d = −0.74, T3 d = −1.00<br>• Disability (Sheehan Disability Scale – SDS): T3 d = −1.22, p = 0.0013<br>• Anxiety (GAD-7): T3 d = −0.98, p = 0.002	Bonferroni correction for multiple comparisons (significance p < 0.00278); paired t-tests for within-group; SAS v9.3.	anxiety), with effects maintained at 6-months. High adherence and acceptability observed. Strong support for MBSR in post-treatment cancer fatigue.
**22**	Johns et al., 2016, USA	Randomized Controlled Trials	Total N = 71 post-treatment breast (n=60) and colorectal (n=11) cancer survivors with moderate-to-severe fatigue; MBSR = 35, Education/Support (ES) = 36	Mindfulness-Based Stress Reduction (MBSR) – 8-week group program including meditation (sitting, body scan, lovingkindness), hatha yoga, and self-regulation strategies. Category: Mindfulness and	MBSR vs. Education and Support (ES) – an active control involving fatigue education and self-care planning	**Primary outcomes (continuous):**<br>• Attentional Function Index (AFI – total and subscales):<br>– Total score: T2 d = 0.83, p = 0.001; T3 d = 0.55, p = 0.021<br>– Effective Action: T2 d = 0.72, p = 0.004; T3 d = 0.55, p = 0.027<br>– Attentional Lapses: T2 d = 0.90, p < 0.001; T3 d = 0.59, p = 0.017<br>– Interpersonal Effectiveness: ns<br>• Stroop accuracy (objective	ANCOVA with baseline scores and covariates (age, education, income, cancer type); paired t-tests and Wilcoxon signed-rank for within-group; bootstrapped mediation; Cohen's d and	MBSR significantly improved perceived cognitive functioning and Stroop accuracy over ES, with durable effects at 6 months. Acting with awareness, observing, and non-reactivity mediated improvements in AFI. Supports use of MBSR for cancer-related cognitive impairment

No.	Authors	Design	Population	Intervention	Comparison	Outcome	Methods	Conclusion
				Stress Reduction Intervention.		cognitive function): T2 r = 0.34, p = 0.005; T3 r = 0.28, p = 0.030<br>• Stroop Reaction Time (RT): not significant<br>**Mediators:**<br>• Increases in Five Facet Mindfulness Questionnaire (FFMQ) subscales: Acting with Awareness, Observing, Non-reactivity	Standardized Response Mean (SRM) effect sizes; SAS software.	(CRCI) among survivors with fatigue.
**23**	Kenne et al., 2017, Sweden	Randomized Controlled Trials	Total N = 166 women diagnosed with breast cancer post-adjuvant therapy; MBSR = 62, Active Controls = 52 (self-instructing MBSR), Non-MBSR = 52; mean age 57.2 (SD = 10.2)	Mindfulness-Based Stress Reduction (MBSR) – 8-week standardized group program with weekly sessions + daily homework; included meditation, yoga, reflection.<br>Category: Mindfulness and Stress Reduction Intervention.	MBSR vs. Active Controls (self-instructing MBSR) vs. Non-MBSR (no intervention)	**Primary outcome (continuous):**<br>• Depression (Hospital Anxiety and Depression Scale – HADS):<br>– MBSR: 4.3 → 3.3 (p = 0.001); Between-group (vs. non-MBSR): p = 0.015<br>– No significant change in anxiety.<br>**Secondary outcomes:**<br>• Symptom Experience (Memorial Symptom Assessment Scale – MSAS):<br>– Psychological symptoms: p = 0.008 (within), p = 0.019 (between)<br>– Physical: p = 0.007; Total burden: p = 0.004<br>• Health Status (SF-36): Vitality ↑ (p < 0.001), Physical Function ↑ (p < 0.001), Mental Health ↑ (p < 0.001)	Nonparametric statistics (Wilcoxon and Mann–Whitney tests); Spearman correlations; Analysis of flow cytometry for immune cell phenotyping; ELISA for cytokines.	MBSR led to significant improvements in depression, psychological symptoms, vitality, coping, mindfulness and posttraumatic growth, and immune function (NK activity, CD19+). Strong support for MBSR as effective intervention for psychological and biological outcomes in breast cancer survivorship.

No.	Authors	Design	Population	Intervention	Comparison	Outcome	Methods	Conclusion
						• Coping (Sense of Coherence – SOC): p = 0.028<br>• Mindfulness (Five Facet Mindfulness Questionnaire – FFMQ): Observe and Nonreactivity improved (p < 0.001)<br>• Posttraumatic Growth (Posttraumatic Growth Inventory – PTGI): p = 0.005<br>• Biological: NK-cell activity ↑ (p = 0.015); CD19+ B-cells ↑ (p = 0.001); Cytokines (IL-6, IL-8): NS		
**24**	Lengacher et al., 2016, USA	Randomized Controlled Trials	Total N = 322 breast cancer survivors (Stage 0–III); MBSR = 167, Usual Care = 155; aged 21+; within 2 weeks to 2 years post-treatment	Mindfulness-Based Stress Reduction for Breast Cancer (MBSR[BC]) – 6-week adapted program with weekly 2-hour sessions; meditation (sitting, walking, body scan), Hatha yoga, group discussions. Category: Mindfulness and Stress Reduction Intervention.	MBSR vs. Usual Care (standard post-treatment clinical visits; MBSR offered post-study)	**Primary outcomes (continuous):**<br>• Anxiety (State-Trait Anxiety Inventory – State): d = 0.27 (T3), p = 0.007<br>• Fear of Recurrence – Overall (Concerns About Recurrence Scale – CARS): d = 0.28, p = 0.001<br>• Fear of Recurrence – Problems: d = 0.35, p = 0.001<br>• Depression (Center for Epidemiologic Studies Depression Scale – CES-D): p = 0.06<br>• Fatigue severity (Fatigue Symptom Inventory – FSI): d = 0.27, p = 0.002<br>• Fatigue interference (FSI): d =	Linear mixed models with full-information maximum-likelihood estimation (Mplus v7.1); adjusted for anxiety medication use; sensitivity analysis (correlation structures); intent-to-treat; moderator analysis for perceived stress;	MBSR(BC) significantly improved anxiety, fear of recurrence (overall and problems), and fatigue (severity and interference) at both 6 and 12 weeks. Depression and pain did not significantly differ. Effects strongest for those with high baseline stress. Supports MBSR(BC) for alleviating multiple co-occurring psychological and physical symptoms in breast cancer survivorship.

No.	Authors	Design	Population	Intervention	Comparison	Outcome	Methods	Conclusion
						0.23, p = 0.006<br>• Pain (Brief Pain Inventory – BPI): not significant<br>• Quality of Life (Medical Outcomes Study Short Form – MOS SF-36): p = 0.05	p < .01 threshold for significance.	
**25**	Mirmahmoodi et al., 2020, Iran	Randomized Controlled Trials	Total N = 44 women with breast cancer (non-metastatic); MBSR = 22, Control = 22; age range: 18–70; post-chemotherapy or surgery; recruited from multiple oncology centers in Kerman, Iran	Mindfulness-Based Stress Reduction (MBSR) – 8-week group counseling, 90-min sessions; includes body scan, sitting meditation, breathing, mindful walking, yoga. Category: Mindfulness and Stress Reduction Intervention.	MBSR vs. Routine care (consultation with nurses/psychologists, referral info only, no structured psychosocial intervention)	**Primary outcomes (continuous):**<br>• Anxiety (Beck Anxiety Inventory – BAI):<br>– Intervention: 31.18 ± 14.05 → 23.50 ± 11.35 (p = 0.007)<br>– Control: 25.41 ± 14.99 → 35.00 ± 13.52 (p = 0.006)<br>– Between-group post-test: p = 0.004; ANCOVA: p = 0.01<br>• Depression (Beck Depression Inventory-II – BDI-II):<br>– Intervention: 29.00 ± 13.01 → 17.18 ± 9.46 (p < 0.001)<br>– Control: 21.04 ± 11.17 → 21.59 ± 11.97 (ns); ANCOVA: p = 0.15<br>• Perceived Stress (Perceived Stress Scale – PSS):<br>– Intervention: 33.68 ± 7.29 → 28.09 ± 4.82 (p = 0.001)<br>– Control: 28.64 ± 3.00 → 28.04 ± 2.28 (ns); ANCOVA: p = 0.14<br>• Inflammatory markers:<br>– Cortisol (μg/dL): ns (ANCOVA p = 0.08)	SPSS v18; Wilcoxon and Mann–Whitney for stress; independent and paired t-tests for anxiety, depression, CRP, cortisol; ANCOVA for confounding adjustment (e.g., treatment type); significance level: p < .05.	MBSR significantly reduced anxiety; clinical but not statistical improvement in depression and perceived stress. No significant effect on cortisol or CRP. Supports MBSR use for anxiety management in breast cancer patients; more studies needed on physiological markers.

No.	Authors	Design	Population	Intervention	Comparison	Outcome	Methods	Conclusion
						– CRP (mg/L): ns (ANCOVA p = 0.61)		
**26**	Reich et al., 2017, USA	Randomized Controlled Trials	Total N = 322 breast cancer survivors (Stage 0–III); MBSR = 167, Usual Care = 155; age ≥ 21; within 2 years post-treatment; exclusion: stage IV or recurrence	Mindfulness-Based Stress Reduction for Breast Cancer (MBSR[BC]) – 6-week program with 2-hour weekly sessions; includes education, group meditation (sitting, walking, yoga, body scan), and informal practices. Category: Mindfulness and Stress Reduction Intervention.	MBSR(BC) vs. Usual Care (routine clinical follow-up, no psychosocial intervention)	**Primary outcomes (continuous):**<br>• Psychological Cluster (Depression – CESD, Anxiety – STAI, Stress – PSS, Emotional Wellbeing – SF-36): MBSR(BC) vs. UC – significant improvement ($p = 0.007$), Cohen's $d = 0.35$<br>• Fatigue Cluster (Fatigue – FSI, Sleep – PSQI, Drowsiness – MDASI): MBSR(BC) vs. UC – significant improvement ($p < 0.001$), Cohen's $d = 0.50$<br>• Pain Cluster (Brief Pain Inventory, SF-36 pain): no significant difference ($p = 0.97$)<br>• Cognition Cluster (ECog, CAMS-R): no significant difference ($p = 0.78$)<br>Effects sustained but not enhanced at 12 weeks follow-up.	Structural Equation Modeling (SEM) with latent variable modeling (Mplus v7.1); factor analysis for symptom clusters; RMSEA, CFI, SRMR for model fit; intent-to-treat analysis; adjusted for baseline differences using difference scores.	MBSR(BC) significantly improved psychological and fatigue symptom clusters with medium effect sizes during the intervention; effects maintained but not further improved at 12 weeks. No significant effects on pain or cognition. Highlights cluster-targeted benefits of MBSR(BC).
**27**	Shergill et al., 2022, Canada	Randomized Controlled Trials	Total N = 98 women breast cancer survivors with chronic neuropathic pain; Intervention =	Mindfulness-Based Stress Reduction (MBSR) – 8-week program, 2.5h/week + 6h retreat; includes body scan,	MBSR vs. Waitlist (control group later received MBSR)	**Primary outcome (dichotomous):**<br>• Proportion with ≥1 point reduction on Brief Pain Inventory – Interference: Intervention = 35.5% (11/31), Control = 20.5% (8/39); OR =	Mixed effects logistic and linear regression (SAS v9.4); adjusted for pain severity and etiology;	No significant benefits of MBSR over waitlist control on any outcomes. MBSR did not reduce pain-related disability or psychological distress. Highlights publication of

No.	Authors	Design	Population	Intervention	Comparison	Outcome	Methods	Conclusion
			49, Waitlist = 49; mean age: 51.3 (SD = 11.4) intervention, 55.1 (SD = 9.6) control; average pain duration ~3 years	meditation, discussion. Category: Mindfulness and Stress Reduction Intervention.		1.96, 95% CI [0.60, 6.41], p = 0.2633<br><br>**Secondary outcomes (continuous):**<br>• Pain interference (BPI): No significant difference at 3 months (mean Δ = 0.04, p = 0.9312)<br>• Mood (Profile of Mood States – POMS): NS<br>• Depression (Patient Health Questionnaire-9 – PHQ-9): NS<br>• Pain catastrophizing (Pain Catastrophizing Scale – PCS): NS<br>• Mindfulness (Five Facet Mindfulness Questionnaire – FFMQ): NS<br>• Neuropathic pain (Neuropathic Pain Symptom Inventory – NPSI): NS<br>• Physical/mental health (Short-Form-12 – SF-12): NS<br>• Patient Global Impression of Change (PGIC): NS	stratified randomization; intention-to-treat; exploratory analysis with continuous modeling and pooled intervention data.	null results and need for more tailored interventions for chronic neuropathic pain (CNP).
**28**	Victorson et al., 2020, USA	Randomized Controlled Trials	Total N = 126 young adults with cancer (age 18–39); MBSR = 67, Waitlist = 59;	Mindfulness-Based Stress Reduction (MBSR) – 8-week in-person program (2.5h weekly	MBSR vs. Waitlist control (delayed MBSR after 16 weeks)	**Primary outcomes (continuous):**<br>• Self-Kindness (Self-Compassion Scale – SCS): MBSR group showed significant	SPSS v25; descriptive statistics, bivariate analyses (t-tests,	MBSR was feasible and acceptable for participants who enrolled. Significant improvements in self-

No.	Authors	Design	Population	Intervention	Comparison	Outcome	Methods	Conclusion
			78.6% female; majority had completed treatment; recruited from a Midwestern cancer center	sessions + half-day retreat); includes guided meditation, body scan, yoga. Category: Mindfulness and Stress Reduction Intervention.		improvement over time F(2, 79.48) = 3.64, P = .031, Cohen's d = 0.44<br>• Sleep Disturbance (PROMIS): F(2, 79.75) = 4.39, P = .016, Cohen's d = 0.47<br>• Intolerance of Uncertainty: F(2, 82.34) = 3.50, P = .035, Cohen's d = 0.51<br>• Posttraumatic Growth (PTGI-SF): F(2, 82.95) = 3.41, P = .038, Cohen's d = 0.41<br>• Perceived Stress (PSS-4): not significantly different between groups; within-group change noted<br>• Depression (PROMIS): Within-group change in MBSR, but not between groups<br>• No significant group differences observed for anxiety, fatigue, pain interference, or social support domains	chi-square); longitudinal mixed models adjusted for covariates (meditation history, attendance, follow-up email exposure); intent-to-treat analysis.	kindness, sleep disturbance, intolerance of uncertainty, and posttraumatic growth observed within group. Online format recommended for greater feasibility in young adult cancer survivors.
**INTERVENTION TYPE: Coping and Psychological Resilience**								
PRISM (Promoting Resilience in Stress Management); Coping skills training; Therapeutic journaling, art therapy, or narrative therapy (if validated and quantifiable)								
**29**	Cafaro et al., 2024 Italy	Randomized Controlled Trials	Total N = 102 cancer patients (breast or colon, stages I–III); randomized: GDP = 49,	Guided Disclosure Protocol (GDP) – 3 writing sessions, 20 minutes each, every two weeks; structured	GDP vs. Generic Writing (non-emotional, detached factual writing)	**Primary outcome (continuous):**<br>• Post-traumatic Growth (Post-traumatic Growth Inventory – PTGI): assessed at T0 (baseline), T1 (3 months), and T2 (6 months).	SPSS; 2×3 mixed factorial ANOVA to assess time × group interactions;	GDP did not show statistically significant effects on post-traumatic growth or psychological distress outcomes. Study likely underpowered;

No.	Authors	Design	Population	Intervention	Comparison	Outcome	Methods	Conclusion
			control writing = 53; 79.4% women; mean age ~56; recruited across five cancer centers in Italy	reflection on illness and life meaning. Category: Coping and Psychological Resilience Interventions.		**Secondary outcomes (continuous):**<br>• Meaning-making (Constructed Meaning Scale – CMS)<br>• Psychological distress (Impact of Event Scale – IES)<br>• Emotional distress (Hospital Anxiety and Depression Scale – HADS)<br>Results: no statistically significant group-by-time effects. Mean PTGI difference at T1: −5.8 [95% CI −14 to 2.4], p = 0.166. Similar non-significant results for CMS, IES, and HADS.	mean differences tested with independent t-tests; primary outcome PTGI, secondary outcomes CMS, IES, HADS; intent-to-treat analysis.	trends suggest GDP may enhance PTG. Larger trials recommended to confirm efficacy.
**30**	Cheung et al., 2017, USA	Randomized Controlled Trials	Total N = 39 women with metastatic breast cancer; In-person LILAC = 14, Online LILAC = 12, In-person control = 13; mean age = 53.35 (SD = 11.22); at UCSF Cancer Center; post-diagnosis	Lessons in Linking Affect and Coping (LILAC) – 5-session intervention (1 h/week), skills include noticing positive events, gratitude, mindfulness, positive reappraisal, self-kindness. Delivered in-person or online. Category: Coping	LILAC (in-person/online) vs. attention-matched in-person control (qualitative thematic sessions, no skills training)	**Primary outcomes (continuous):**<br>• Depressive Mood (Center for Epidemiologic Studies Depression Scale – CES-D):<br>– LILAC group: M = 18.58 → 13.64 → 11.61; p = 0.03; d = −0.81 (1-month follow-up)<br>– Control group: M = 20.62 → 17.82 → 17.10; p > 0.42<br>• Negative Affect (Differential Emotions Scale – DES):<br>– LILAC group: M = 1.26 → 0.85 → 0.87; p < 0.02; d = −0.81	Longitudinal growth modeling; one-sample t-tests vs. clinical threshold; SPSS v24; analysis of within-group change and between-group contrasts (baseline to post and 1-month follow-up);	LILAC showed feasibility, acceptability, and reduced depressive symptoms and negative affect. Online format was comparably effective to in-person delivery. Intervention supports use of positive-affect skills in metastatic cancer coping. Larger trial needed.

No.	Authors	Design	Population	Intervention	Comparison	Outcome	Methods	Conclusion
			time ~2.58 years; 64% receiving active treatment	and Psychological Resilience Interventions.		• Positive Affect: NS<br>• Cancer-specific QoL (Multidimensional Quality of Life Scale – Cancer Version): NS<br>**Positive coping outcomes (continuous):**<br>• Positive-Affect Skill Use, Mindfulness, Self-Compassion (Self-Compassion Scale – Short Form):<br>– LILAC: medium-to-large within-group effects (0.50 < d > 0.91), p < 0.08<br>– Control: NS; Between-group differences not statistically significant	effect sizes (Cohen's d).	
**31**	Graboyes et al., 2023, USA	Randomized Controlled Trials	Total N = 44 head and neck cancer survivors with body image distress (BID); BRIGHT = 20, AC = 24; mean age = 63; 61% female; 50% oral cavity cancer; 61% stage III/IV; 61% received adjuvant therapy	BRIGHT (Building a Renewed ImaGe after Head & neck cancer Treatment) – 5-session weekly tele-CBT delivered by psychologist; focuses on psychoeducation, self-monitoring, cognitive restructuring, coping strategies, relapse prevention. Category: Coping	BRIGHT vs. Attention Control (AC) – 5-session educational tele-support program unrelated to body image	**Primary outcome (continuous):**<br>• Head and Neck Cancer–related Body Image Distress (IMAGE-HN): Mean = 43 (SD = 18) at baseline; changes measured at 1 and 3 months<br>• Cancer-related Body Image Distress (Body Image Scale): Mean = 17 (SD = 6) at baseline<br>**Mediators (continuous):**<br>• Body Image Coping Skills (Body Image Coping Skills Inventory – BICSI):<br>– Avoidance subscale (0–24): BRIGHT vs AC Δ = −2.8, 90% CI	Linear models and causal mediation analyses with 90% CI; RStudio; adjusted for baseline IMAGE-HN and BICSI values. Simulation approach (mediation package); ANCOVA-type	BRIGHT significantly reduced avoidant body image coping, mediating reduction in BID at 3 months. Preliminary evidence supports CBT targeting maladaptive coping as a mechanism. Larger trials needed for efficacy and generalization.

No.	Authors	Design	Population	Intervention	Comparison	Outcome	Methods	Conclusion
				and Psychological Resilience Interventions.		[−4.9, −0.7], p = 0.029 (1-month)<br>– Appearance Fixing subscale (0–30): Δ = −2.6, 90% CI [−5.4, 0.2], p = 0.13<br>– Positive Rational Acceptance subscale (0–33): Δ = 0.9, 90% CI [−1.9, 3.7], p = 0.60<br>Significant causal mediation: Decrease in BICSI-Avoidance at 1 month mediates BRIGHT's effect on IMAGE-HN at 3 months (Δ = −4.5, 90% CI [−9.7, −0.6])	regression for between-group comparisons.	
**32**	Jensen-Johansen et al., 2018, Denmark	Randomized Controlled Trials	Total N = 507 Danish women with primary breast cancer, stage I–II; randomized: EWI = 253, CTRL = 254; age range: 27–70; post-surgery (lumpectomy or mastectomy), with/without adjuvant therapy	Expressive Writing Intervention (EWI) – 3×20 min home-based writing sessions over 3 weeks; emotional disclosure about traumatic experiences (own cancer or other). Category: Coping and Psychological Resilience Interventions.	EWI vs. Neutral Writing Control (non-disclosing factual writing about daily routines)	**Primary outcomes (continuous):**<br>• Physical Symptoms (Patient Health Questionnaire – PHQ-15):<br>– No significant group × time interaction; PHQ mean scores T1: 6.81 (SD = 4.02) vs. 6.40 (SD = 4.03); T2: 7.00 vs. 6.62; T3: 7.08 vs. 6.72<br>• Healthcare utilization (monthly rate):<br>– GP Visits: EWI vs CTRL at T1 = 2.08 vs. 2.03; T2 = 1.61 vs. 1.66; T3 = 1.21 vs. 1.32<br>– GP Phone Calls: EWI vs CTRL at T1 = 1.12 vs. 1.23; T2 = 0.89 vs. 0.86; T3 = 0.80 vs. 0.95	Multilevel linear modeling (MLM) and negative binomial models for count data; intent-to-treat; moderation analyses with repressive coping (MCSD-TMAS), alexithymia (TAS-20), social constraints (SCS), rumination	EWI showed no significant effects on physical symptoms or healthcare use. Subgroup benefits were identified for low-alexithymic participants and those writing about their own cancer. General application of EWI for physical health not supported; targeted use may be beneficial.

No.	Authors	Design	Population	Intervention	Comparison	Outcome	Methods	Conclusion
						**Moderation (continuous):**<br>• Alexithymia (Toronto Alexithymia Scale – TAS-20): Significant moderation for GP phone calls (p = 0.029)<br>• Writing topic (own cancer vs. other): Women writing about own cancer showed greater decrease in GP use (p = 0.012/0.001)<br>• No significant moderation by repressive coping, social constraints, or rumination	(ECQ); SPSS v24 and Stata v14.	
**33**	Lu et al., 2023, USA	Randomized Controlled Trials	Total N = 136 Chinese American breast cancer survivors (CABCS); randomized: ESR = 54, SR = 46, CF = 36; mean age ≈ 58; inclusion: completed treatment ≤5 years ago, Mandarin or Cantonese speakers	Enhanced Self-Regulation (ESR): 3 weekly expressive writing prompts (Week 1: stress/coping; Week 2: emotions; Week 3: benefits); culturally adapted order. Category: Coping and Psychological Resilience Interventions.	ESR vs. Self-Regulation (SR) vs. Cancer-Facts (CF) control group	**Primary outcomes (continuous):**<br>• Depressive symptoms (Center for Epidemiologic Studies Depression Scale – CES-D):<br>– ESR vs. CF: significant reductions at 1, 3, and 6 months (b from −1.60 to −2.22, p < .05)<br>• Anxiety symptoms (Brief Symptom Inventory – BSI-Anxiety Subscale):<br>– ESR vs. CF: reductions at all timepoints (b from −0.11 to −0.17, p < .05)<br>**Mediators (continuous):**<br>• Perceived stress (Perceived Stress Scale – PSS): ESR vs. CF –	Residual change regression and multiple mediation modeling (SPSS PROCESS macro); intent-to-treat; bootstrapping (5,000 resamples); linguistic content analysis using LIWC2007 with Chinese dictionary.	Only the ESR condition significantly reduced depressive and anxiety symptoms vs. CF; SR not significantly different. Perceived stress fully mediated ESR effects. Ordering of prompts critical in culturally adapted interventions.

No.	Authors	Design	Population	Intervention	Comparison	Outcome	Methods	Conclusion
						significant reductions at 1, 3, and 6 months (b = −1.06 to −0.91)<br>• Intrusive thoughts (Impact of Event Scale – IES-Intrusion): ESR vs. CF – reduced at 1-month (b = −2.05)<br><br>**Mediation:**<br>• Perceived stress significantly mediated effect of ESR on depressive and anxiety symptoms (95%CI did not contain 0)<br>• Intrusive thoughts did not mediate outcomes		
**34**	Nairn and Merluzzi, 2019, USA	Randomized Controlled Trials	Total N = 134 adult cancer patients receiving active treatment; randomized: intervention = 66, control = 68; screened for low coping self-efficacy (CBI < 245); multiple cancer types; mean age ≈ 56	Mastery Enhancement Therapy: 4-session intervention based on self-regulation and self-efficacy theory; personalized recall of successful coping behaviors; integrated elements of Solution Focused Therapy. Category: Coping and Psychological	Mastery Enhancement Therapy vs. usual medical care (control)	**Primary outcomes (continuous):**<br>• Self-efficacy for coping with cancer (Cancer Behavior Inventory – CBI):<br>– Significant Group × Time interaction ($F = 2.79$, $p = .04$); greater gains in treatment group by post-test (T3), equalized by follow-up (T4)<br>**Secondary outcomes (continuous):**<br>• Depression (Beck Depression Inventory – BDI):<br>– Three-way interaction Group × Time × Symptom Frequency (F =	Multilevel modeling with unstructured covariance matrix; covariate: Symptom Impact Inventory – Frequency (SII-F); effect sizes calculated; fidelity rating of recorded sessions; intent-to-treat sample.	Mastery Enhancement Therapy significantly accelerated self-efficacy for coping, with delayed benefits in depression and adjustment for high-symptom participants. Results support integration into routine oncology care with brief, tailored sessions.

No.	Authors	Design	Population	Intervention	Comparison	Outcome	Methods	Conclusion
				Resilience Interventions.		2.66, p = .05): treatment more effective at T4 among patients with high symptoms<br>• Psychosocial Adjustment (Psychosocial Adjustment to Illness Scale – PAIS):<br>– Three-way interaction (F = 3.14, p = .03): greater gains for high-symptom treatment participants<br>• Quality of life (Functional Assessment of Cancer Therapy – General – FACT-G):<br>– Small ES (d = .24) but non-significant<br>• CBI Subscales: Significant Time × Group interactions on Coping with Side Effects and Accepting Cancer scales		
**35**	Nelson et al., 2021, USA	Randomized Controlled Trials	Total N = 160 adults with high-risk acute myeloid leukemia (AML) undergoing intensive chemotherapy; IPC = 86, usual care = 74; median age = 64.4 years; 60%	Integrated Palliative and Oncology Care (IPC): at least 2×/week consultations during hospitalization addressing symptoms, coping, goals of care, and end-of-life	IPC vs. Usual Care (standard leukemia treatment, with palliative care only upon request)	**Primary outcomes (continuous):**<br>• Quality of life (Functional Assessment of Cancer Therapy–Leukemia – FACT-Leukemia):<br>– IPC vs. usual care at week 2: B = 8.86, SE = 4.26, P < .05<br>– Mediated by approach-oriented coping: B = 5.13, SE = 2.35, 95% CI [1.25, 10.44]<br>• Depression symptoms (Hospital Anxiety and	Linear regression and linear mixed-effects models; parallel multiple mediation models with bootstrapped 95% CI; Stata 9.3; intent-to-treat sample;	IPC improved QOL and reduced depression/anxiety at week 2; effects were mediated primarily through enhanced approach-oriented coping. Avoidant coping did not significantly mediate outcomes. Highlights coping as a

No.	Authors	Design	Population	Intervention	Comparison	Outcome	Methods	Conclusion
			male; 86.2% White; inclusion: newly diagnosed, relapsed, or refractory AML; hospitalized	planning. Category: Coping and Psychological Resilience Interventions.		Depression Scale – HADS-D):<br>– IPC vs. usual care at week 2: B = –1.52, SE = 0.65, P < .05<br>– Mediated by approach-oriented coping: B = –0.88, SE = 0.37, 95% CI [–1.67, –0.26]<br>• Anxiety symptoms (Hospital Anxiety and Depression Scale – HADS-A):<br>– IPC vs. usual care at week 2: B = –1.41, SE = 0.59, P < .05<br>– Mediated by approach-oriented coping: B = –0.37, SE = 0.23, 95% CI [–0.93, –0.01]<br>**Mediators (continuous):**<br>• Approach-oriented coping (Brief COPE): B = 1.85, SE = 0.62, P = .004<br>• Avoidant coping: B = –0.70, SE = 0.29, P = .02	adjustment for baseline scores.	key mechanism of palliative care in AML.
**36**	Rosenberg et al., 2018, USA	Randomized Controlled Trials	Total N = 92 adolescents and young adults (AYAs) with cancer, aged 12–25; PRISM = 48, UC = 44; 73% aged 12–17; 62% with leukemia/lymph	Promoting Resilience in Stress Management (PRISM): 4×30–50 min sessions biweekly, targeting mindfulness, goal setting, cognitive reframing, and meaning-making. Optional family	PRISM vs. Usual Care (UC) – standard psychosocial oncology care at pediatric AYA center	**Primary outcomes (continuous):**<br>• Resilience (Connor-Davidson Resilience Scale – CDRISC-10): Δ = +3.0 points; 95% CI [0.5–5.4], P = .02<br>**Secondary outcomes (continuous):**<br>• Cancer-specific quality of life (Pediatric Quality of Life Cancer Module – PedsQL-CM): Δ = +9.6;	Unadjusted linear and logistic mixed-effects regression models with random intercepts/slopes; intent-to-treat; Cohen's d effect sizes; MCID = ½	PRISM significantly improved resilience, cancer-specific quality of life, and reduced distress vs. UC. Depression trends favored PRISM. Findings support brief, skill-based interventions to enhance psychosocial well-being among AYA with cancer.

No.	Authors	Design	Population	Intervention	Comparison	Outcome	Methods	Conclusion
			oma; 26% with advanced cancer	meeting and monthly boosters. Delivered by trained lay staff. Category: Coping and Psychological Resilience Interventions.		95% CI [2.6–16.7], P = .01<br>• Generic quality of life (PedsQL SF-15): Δ = +7.2; 95% CI [–0.8 to 15.2], P = .08<br>• Psychological distress (Kessler-6): Δ = –2.1; 95% CI [–4.1 to –0.2], P = .03<br>• Depression (Hospital Anxiety and Depression Scale – HADS-D): PRISM = 6% vs UC = 21%; OR = 0.09; 95% CI [0.01–1.09], P = .06<br>• Anxiety (HADS-A): PRISM = 17% vs UC = 26%; OR = 0.36; 95% CI [0.07–1.96], P = .24	SD of baseline; conducted using Stata 14.	
**37**	Rosenberg et al., 2021, USA	Randomized Controlled Trials	Total N = 92 adolescents and young adults (AYAs) with cancer; PRISM = 48, UC = 44; analysis at 24 months: n = 57 survivors; age 13–25; 65% leukemia/lymphoma; 26% advanced disease	Promoting Resilience in Stress Management (PRISM): 4 sessions (stress management, goal setting, reframing, meaning-making), each 20–50 mins, delivered biweekly by trained coaches. Category: Coping and Psychological Resilience Interventions.	PRISM vs. Usual Care (standard psychosocial support)	**Primary outcomes (continuous):**<br>• Cancer-related quality of life (Pediatric Quality of Life Inventory – PedsQL): PRISM vs. UC at 6 months: β = 9.1 (95% CI: 2.8 to 15.4, P = .01); 12 months: β = 7.4 (95% CI: 0.8 to 14, P = .03); 24 months: β = 5.9 (95% CI: –1.1 to 12.9, P = .10)<br>• Hope (Hope Scale): 6 months: β = 3.5 (95% CI: 0.3 to 6.8, P = .04); 12 months: β = 6.2 (95% CI: 2.7 to 9.6, P < .001); 24 months: β = 4.6 (95% CI: 1.0 to 8.3, P = .01)<br>• Resilience (Connor-Davidson	Linear mixed-effects regression models with group-by-time interaction; random intercepts; Kenward-Roger approach; R software; intent-to-treat; subgroup follow-up with repeated measures.	PRISM was associated with sustained improvements in quality of life and hope over 24 months; resilience and distress effects not statistically significant, but trends favored PRISM. Early response predicted longer-term well-being benefits.

No.	Authors	Design	Population	Intervention	Comparison	Outcome	Methods	Conclusion
						Resilience Scale – CDRISC-10): NS across timepoints<br>• Psychological distress (Kessler-6): NS across timepoints<br>• Sustained improvements from 6 to 24 months: QoL (76% PRISM vs 47% UC), Hope (58% vs 43%), Resilience (50% vs 29%), Distress (74% vs 32%)		
**38**	Samami et al., 2021, Iran	Randomized Controlled Trials	Total N = 60 women with breast cancer; Intervention = 30, Control = 30; Final analyzed: Intervention = 27, Control = 30; Age M = 43.81 (SD 7.41); undergoing chemotherapy	Supportive Program: 6 weekly 90-min group sessions, covering education, stress management, relaxation techniques, coping strategies (problem- and emotion-focused), social and spiritual support. Delivered by MSc counselor under psychiatric supervision. Category: Coping and Psychological Resilience Interventions.	Supportive Program vs. Routine Care (standard education by nurse or oncologist on physical/nutritional issues post-chemotherapy)	**Primary outcomes (continuous):**<br>• Problem-Focused Coping (PFC; Ways of Coping Questionnaire – WOCQ):<br>– Immediately post-intervention: Intervention = 73.69 ± 7.62; Control = 46.57 ± 11.70; Effect size = 2.75; P < .001<br>– One month post-intervention: Intervention = 72.73 ± 7.56; Control = 46.57 ± 12.63; Effect size = 2.48; P < .001<br>• Emotion-Focused Coping (EFC; WOCQ):<br>– Immediately post-intervention: Intervention = 41.92 ± 6.42; Control = 55.14 ± 8.44; Effect size = 1.70; P < .001<br>– One month post-intervention: Intervention = 40.32 ± 6.34;	Generalized Estimating Equations (GEE) to evaluate intervention effects over time; Mann–Whitney U test, independent t test for group comparisons; SPSS v20; significance level set at p < 0.05.	The supportive program significantly improved problem-focused coping and reduced emotion-focused coping and stress levels both immediately and one month after the intervention, with large to very large effect sizes. The findings support structured supportive care as an effective non-pharmacological strategy for women with breast cancer.

No.	Authors	Design	Population	Intervention	Comparison	Outcome	Methods	Conclusion
						Control = 53.08 ± 8.60; Effect size = 1.67; P < .001<br>**Secondary outcomes (continuous):**<br>• Stress (Depression-Anxiety-Stress Scale – DASS-21):<br>– Immediately post-intervention: Intervention = 8.59 ± 3.17; Control = 15.86 ± 4.19; Effect size = 1.94; P < .001<br>– One month post-intervention: Intervention = 8.22 ± 2.95; Control = 17.26 ± 4.28; Effect size = 2.43; P < .001		
**39**	Santoyo-Olsson et al., 2022, USA	Randomized Controlled Trials	N = 151 Spanish-speaking Latina women with nonmetastatic breast cancer, within 1 year of diagnosis, recruited from 5 counties in Northern California	Nuevo Amanecer (A New Dawn) stress management intervention – peer-delivered, cognitive behavioral strategies; focus on coping resource identification. Category: Coping and Psychological Resilience Interventions.	Descriptive comparison at baseline (no comparison intervention group analyzed here)	**Primary outcomes (continuous):**<br>• Health distress (Stanford Patient Education Research Center scale): M = 3.14 (SD = 1.03)<br>• Anxiety (Brief Symptom Inventory – 6 items): M = 0.97 (SD = 0.86)<br>**Regression findings:**<br>• Self-efficacy for managing breast cancer treatment: β = –0.11, SE = 0.05, p < 0.05 (health distress); β = –0.12, SE = 0.04, p < 0.01 (anxiety)<br>• Coping confidence: β = –0.34, SE = 0.11, p < 0.01 (health	Hierarchical linear regression modeling; bivariate correlations; SAS v9.4; model fit via R^2 and p-values; variable selection based on literature support and bivariate p < 0.2.	Intrapersonal (self-efficacy, coping confidence) and interpersonal (neighborhood cohesion) coping resources were inversely associated with distress. Interventions enhancing control and environmental support are critical for reducing psychosocial morbidity in low-income Spanish-speaking Latinas with breast cancer.

No.	Authors	Design	Population	Intervention	Comparison	Outcome	Methods	Conclusion
						distress)<br>• Perceived neighborhood cohesion: β = –0.19, SE = 0.07, $p < 0.01$ (health distress)<br>• Financial hardship and English-language acculturation positively associated with anxiety<br>• Organizational/community level coping resources: not significant predictors		
**40**	Tutino et al., 2022, USA	Randomized Controlled Trials	Cancer and Aging Reflections for Elders – Expressive Writing Intervention (CARE-Express): 5×45 min sessions over 7 weeks, telephone-delivered writing sessions by trained writing mentors from MSK's Visible Ink program. Based on coping	CARE-Express vs. Enhanced Social Work Control (ESWC): 5 phone sessions with social worker specialized in geriatrics (historical control)	**Primary outcomes (continuous):**<br>• Depression (Hospital Anxiety and Depression Scale – HADS): d = 0.69, p = 0.01<br>• Demoralization (Demoralization Scale – Total): d = 0.50, p = 0.06<br>• Spiritual well-being (Functional Assessment of Chronic Illness Therapy –	ANCOVA with baseline adjustment, t-tests, effect sizes (Cohen's d); feasibility based on Leon et al. criteria (≥80% retention, fidelity); SAS used for analyses.	CARE-Express was feasible, with high retention and fidelity. It showed moderate effects in reducing depression and demoralization, and improving spiritual well-being. Results support its accessibility and potential utility in older cancer patients.	

No.	Authors	Design	Population	Intervention	Comparison	Outcome	Methods	Conclusion
			theory and developmental psychology. Category: Coping and Psychological Resilience Interventions.		Spiritual Well-Being, FACIT-Sp-12):<br>– Total: d = 0.41, p = 0.07; Meaning/Peace: d = 0.32, p = 0.20; Faith: d = 0.35, p = 0.07<br>• Behavioral disengagement (COPE): d = 0.44, p = 0.06<br>• Anxiety (HADS): d = 0.08; Loneliness (UCLA): d = 0.01 – not significant<br>**Four-month follow-up:**<br>• Depression: d = 0.24; Demoralization (Loss of Meaning): d = 0.31; Sense of Failure: d = 0.30<br>• FACIT-Sp-12: Total: d = 0.27; Faith: d = 0.33			

No.	Authors	Design	Population	Intervention	Comparison	Outcome	Methods	Conclusion
**41**	Winger et al., 2023, USA	Randomized Controlled Trials	Total N = 59 patients with stage IV solid tumor cancers and moderate-severe pain; MCPC = 29, Usual Care = 30; Mean age = 61 (SD 11.1); 53% female; various cancer types incl. prostate, breast, colorectal, lung	Meaning-Centered Pain Coping Skills Training (MCPC): 4×60 min weekly sessions delivered remotely by trained therapist; integrates Pain Coping Skills Training and Meaning-Centered Psychotherapy. Category: Coping and Psychological Resilience Interventions.	MCPC + Usual Care vs. Usual Care only (standard medical treatment and written support materials)	**Primary outcomes (continuous):**<br>• Pain Severity (Brief Pain Inventory – BPI): 10-weeks: MCPC = 3.01 (95% CI: 2.38–3.65); UC = 4.55 (3.92–5.18); Cohen's d = −0.75 (−1.36, −0.14)<br>• Pain Interference (PROMIS-PI): 10-weeks: MCPC = 58.71 (56.84–60.58); UC = 63.30 (61.41–65.19); d = −0.82 (−1.45, −0.20)<br>• Pain Self-Efficacy (CPSS): 10-weeks: MCPC = 59.38 (52.63–66.13); UC = 46.89 (40.20–53.59); d = 0.74 (0.13, 1.35)<br>• Spiritual Well-being (FACIT-Sp-12): Meaning: d = 0.24; Peace: d = 0.45; Faith: d = 0.12<br>• Psychological distress: Anxiety (GAD-7): d = −0.23; Depression (PHQ-8): d = −0.29; Hopelessness (HAIQ): d = −0.09	Repeated measures linear models (PROC MIXED, SAS v9.4); intention-to-treat; Cohen's d effect sizes; feasibility metrics pre-defined; no hypothesis testing conducted due to pilot design.	MCPC was highly feasible with strong retention and engagement. Moderate-to-large effect sizes indicated improvements in pain severity, interference, and self-efficacy. Modest gains in spiritual well-being and reduced distress. Supports further efficacy trials.
**42**	Wittmann et al., 2022, USA	Randomized Controlled Trials	N = 142 couples (Intervention = 60, Control = 82) coping with localized prostate cancer. Median age = 61. Most had surgery (85%),	TrueNTH Sexual Recovery Intervention – 6-module web-based intervention tailored by treatment type and sexual orientation; focused on	Online intervention vs. access to American Cancer Society's standard webpage on sexuality after cancer	**Primary outcome (continuous):**<br>• Global Satisfaction With Sex Life (PROMIS):<br>– 6 months: Patients: 53 (95% CI: 51–55) vs. Control: 51 (49–53), p = 0.4<br>– Partners: 53 (50–56) vs. Control: 55 (52–57), p = 0.5	T-tests and ANCOVA (baseline-adjusted) for continuous outcomes; χ^2 tests for categorical activity	No significant differences in sexual satisfaction, but greater engagement in sexual activity (esp. nonpenetrative) was observed in intervention couples. High satisfaction and recommendation rates for the program.

No.	Authors	Design	Population	Intervention	Comparison	Outcome	Methods	Conclusion
			followed by radiation (11%), and combined therapy (4%).	education, emotional and sexual recovery. Category: Coping and Psychological Resilience Interventions.		**Secondary outcomes:**<br>• Sexual Activity at 3 months:<br>– Nonpenetrative activity increased in 68% (intervention) vs. 53% (control), $p = 0.07$<br>– Partner increase: 73% (intervention) vs. 60% (control), $p = 0.037$<br>• PROMIS Sexual Interest: no significant between-arm differences<br>• EPIC-26 Sexual Domain: no significant between-arm differences<br>• FSFI (Female Sexual Function Index): higher in intervention arm at 3 months, not significant<br>• Use of sexual aids and nonpenetrative activities greater in intervention arm	increases; PROMIS, EPIC-26, FSFI; SAS 9.4 used for analysis.	Highlights importance of addressing sexual recovery post-treatment.

Notes:

- **ACT: Acceptance and Commitment Therapy** – a third-wave behavioral therapy that encourages psychological flexibility by fostering acceptance of unpleasant experiences and commitment to value-based action.
- **ANCOVA:** Analysis of Covariance – a statistical method used to compare group means while controlling for one or more covariates.
- **CALM: Managing Cancer and Living Meaningfully** – a manualized supportive-existential psychotherapy developed for patients with advanced cancer, addressing emotional distress, existential concerns, and adaptation to illness.
- **CBT: Cognitive Behavioral Therapy** – an evidence-based, structured psychotherapeutic approach focused on modifying dysfunctional thoughts, behaviors, and emotional responses through cognitive restructuring and behavioral techniques.
- **eMBCT**: Online Mindfulness-Based Cognitive Therapy.
- **FACIT-Sp**: Functional Assessment of Chronic Illness Therapy – Spiritual Well-being.
- **FCRI**: Fear of Cancer Recurrence Inventory.
- **FFMQ**: Five Facet Mindfulness Questionnaire.

No.	Authors	Design	Population	Intervention	Comparison	Outcome	Methods	Conclusion

- **HADS**: Hospital Anxiety and Depression Scale – a validated questionnaire used to detect and quantify symptoms of anxiety and depression in hospital settings.
- **IES-R**: **Impact of Event Scale – Revised** – a validated instrument for measuring subjective distress caused by traumatic events.
- **IMCP**: Individual Meaning-Centered Psychotherapy.
- **IPT: Interpersonal Therapy** – a time-limited, evidence-based psychotherapy focused on improving interpersonal functioning and resolving current relational problems to reduce psychological symptoms, especially depression.
- **MBCT: Mindfulness-Based Cognitive Therapy** – an integrative approach combining mindfulness training with cognitive-behavioral techniques to prevent relapse in depression and alleviate psychological distress.
- **MBSR: Mindfulness-Based Stress Reduction** – an eight-week, standardized intervention incorporating mindfulness meditation and yoga to reduce stress, anxiety, and somatic symptoms.
- **MDASI-HN**: **MD Anderson Symptom Inventory – Head and Neck module** – a validated symptom assessment tool specifically designed to measure the severity and impact of symptoms in patients with head and neck cancer. It includes both core cancer-related symptoms and site-specific concerns, such as difficulty swallowing and speech problems.
- **MHC-SF**: Mental Health Continuum – Short Form.
- **PRISM: Promoting Resilience in Stress Management** – a structured psychosocial intervention designed to strengthen resilience and adaptive coping skills in patients with serious medical illness, including cancer.
- **PROMIS**: **Patient-Reported Outcomes Measurement Information System** – a standardized system for measuring health status including depression, anxiety, and physical function.
- **RRQ**: Rumination Response Questionnaire.
- **SF-12:** Short Form Health Survey – 12 items.
- **SHARE program**: Spouses coping with the Head And neck Radiation Experience – a dyadic psycho-oncological intervention focusing on self-management, coping, and communication.
- **Wait-list control**: A control group design in which participants do not receive the experimental treatment during the study period but are scheduled to receive it afterward.

References: All studies referenced herein are fully cited within the main body of the manuscript.

Table S5. Risk of Bias Assessment (RoB 2) and Certainty of Evidence Ratings (GRADE) for Included Randomized Controlled Trials.

No.	ID	Study Design	RoB 2 D1 (Judgement/ Support)	RoB 2 D2 (Judgement/ Support)	RoB 2 D3 (Judgement/ Support)	RoB 2 D4 (Judgement/ Support)	RoB 2 D5 (Judgement/ Support)	Overall RoB 2 (Judgement/ Support)	GRADE Certainty (Judgement/ Support)
**INTERVENTION: Structured Psychotherapeutic (evidence-based)** Cognitive Behavioral Therapy (CBT); Acceptance and Commitment Therapy (ACT); Interpersonal Therapy (IPT); Supportive-existential or psychodynamic therapies (e.g., CALM)									
**1**	Breitbart et al., 2018, USA	Randomized Controlled Trials	Low risk – Computer-generated random allocation with stratification by distress level and physical functioning.	Low risk – Adherence to manualized IMCP; fidelity monitored via session recordings; therapists only trained for one condition to prevent contamination.	Low risk – 321 enrolled; attrition described; ITT analysis applied; dropouts balanced across arms.	Low risk – Blinding not feasible, but outcome measures are validated self-reports.	Low risk – Pre-specified primary and secondary outcomes; multiple timepoints analyzed; statistical correction for multiple testing via False Discovery Rate method.	**Low risk** – All domains individually low; rigorous RCT design; fidelity, analysis, and transparency ensured.	**Moderate certainty** – Well-designed RCT with robust methodology, but generalizability limited due to single-center and overrepresentation of women; effect sizes small to moderate. No blinding possible in psychotherapy trials.
**2**	Fauser et al., 2023, Germany	Randomized Controlled Trials	Low risk – Computerized randomization with permuted blocks, sealed opaque envelopes used for allocation concealment; randomization	Some concerns – Structured sessions delivered by a dual-role therapist (study initiator and intervention provider); fidelity check not externally audited;	Low risk – ITT analysis; 160 randomized; 3-month attrition acknowledged and multiple imputation	Some concerns – Patient-reported outcomes via HADS; blinding not feasible; outcomes might be influenced by expectations; no	Low risk – All outcomes pre-specified; subgroup analysis predefined; statistical models appropriate;	**Some concerns** – Some risks in D2 and D4 related to implementation fidelity and blinding; however,	**Low certainty** – No significant group differences; high internal validity but limited generalizability due to single center, dual roles, and small intervention dose.

No.	ID	Study Design	RoB 2 D1 (Judgement/ Support)	RoB 2 D2 (Judgement/ Support)	RoB 2 D3 (Judgement/ Support)	RoB 2 D4 (Judgement/ Support)	RoB 2 D5 (Judgement/ Support)	Overall RoB 2 (Judgement/ Support)	GRADE Certainty (Judgement/ Support)
			occurred after eligibility confirmed.	potential allegiance bias present.	used; no major imbalance reported.	assessor blinding discussed.	results transparently reported.	methodology and analysis remain largely rigorous.	Effect observed only in subgroup with high anxiety.
**3**	Graham et al., 2024, UK	Randomized Controlled Trials	Low risk – Centralized computer-generated randomization; stratified by site, route, and age; allocation via independent CTRU team.	Low risk – Intervention manualized, therapist training well documented, high procedural and ACT fidelity; remote delivery standardized; consistent supervision provided.	Low risk – 79 randomized; >88% data at 6 months; ITT analyses with descriptive outcomes; minimal attrition.	Some concerns – Blinding not possible; patient-reported outcomes (e.g., QoL, distress) could be influenced by expectancy; fidelity of self-assessment not independently validated.	Low risk – Pre-specified outcomes, statistical methods clear, exploratory proof-of-principle design acknowledged; no selective reporting.	**Some concerns** – Strong methodology and execution overall; minor risk from unblinded outcomes.	**Moderate certainty** – Pilot trial with excellent adherence and fidelity; however, small sample size and exploratory design lower confidence in precision of estimated effects.
**4**	Gudenkauf et al., 2015, USA	Randomized Controlled Trials	Low risk – Random sequence generation and allocation by independent coordinator not involved in assessments or intervention; sequence predefined to avoid allocation bias.	Low risk – Manualized protocols delivered by trained master's level clinicians; fidelity monitored by independent psychologists; adherence supervised weekly.	Some concerns – 183 randomized, 138 completed posttest; no imputation of missing data; partial data exclusion (listwise deletion in ANOVA); differences in	Some concerns – Self-reported outcomes, no blinded assessors; possible performance and detection bias; high internal consistency, but no objective verification of outcomes.	Low risk – Predefined outcomes, validated scales, controlled covariates; statistical reporting complete; pre-registered.	**Some concerns** – Well-conducted, but risk from lack of imputation and unblinded self-report outcomes; baseline group imbalance introduces uncertainty.	**Moderate certainty** – Well-structured dismantling trial with clear differential effects for CBT and RT; findings affected by group imbalance and lack of long-term follow-up.

No.	ID	Study Design	RoB 2 D1 (Judgement/ Support)	RoB 2 D2 (Judgement/ Support)	RoB 2 D3 (Judgement/ Support)	RoB 2 D4 (Judgement/ Support)	RoB 2 D5 (Judgement/ Support)	Overall RoB 2 (Judgement/ Support)	GRADE Certainty (Judgement/ Support)
					baseline adjustment measures.				
**5**	Han et al., 2021, China	Randomized Controlled Trials	Some concerns – Randomized block allocation by investigator; sequence not concealed; risk of allocation prediction.	Some concerns – Therapies delivered by same team who conducted study; fidelity not externally verified; no adherence documentation beyond attendance.	Some concerns – Dropout rates: 15% (treatment), 18% (control); no imputation; per-protocol analysis only; unclear handling of missing values.	Low risk – Outcome assessors blinded; validated Chinese versions of DT and PTGI used; measurements consistent across groups.	Low risk – Pre-specified primary/secondary outcomes; ANCOVA used to adjust baselines; trial pre-registered.	**High** – Allocation and analysis limitations due to internal delivery and absence of missing data management; outcome evaluation robust.	**Low certainty** – Promising results but design limitations (e.g., lack of imputation, internal therapist bias, non-concealed allocation) reduce generalizability and confidence.
**6**	Huang et al., 2024, China	Randomized Controlled Trials	Low risk – Random allocation sequence generated by independent statistician using computer; sealed envelopes ensured allocation concealment; outcome assessors blinded.	Low risk – 8-session BA program standardized with fidelity checks via audio recordings; delivered by trained psychological consultant; consistent implementation documented.	Low risk – 139 randomized; 2.2% dropout; ITT applied; missing data minimal and transparently handled.	Low risk – Outcome measures (DT, GAD-7, GSES, BADS-A) validated; assessors blinded; consistent application across conditions.	Low risk – Outcomes pre-specified; mediation and GEE models pre-planned; clear reporting and statistical adjustment for multiple comparisons (Bonferroni).	**Low risk** – Rigorous RCT with excellent implementation, fidelity, and statistical integrity; strong internal validity.	**Moderate certainty** – Strong effect sizes, well-executed RCT; however, generalizability limited due to single-site and cultural context; replication needed across diverse populations.

No.	ID	Study Design	RoB 2 D1 (Judgement/ Support)	RoB 2 D2 (Judgement/ Support)	RoB 2 D3 (Judgement/ Support)	RoB 2 D4 (Judgement/ Support)	RoB 2 D5 (Judgement/ Support)	Overall RoB 2 (Judgement/ Support)	GRADE Certainty (Judgement/ Support)
**7**	Isaka et al., 2021, Japan	Randomized Controlled Trials	Some concerns – Participants randomized via envelope method, but full allocation sequence and concealment not detailed; partial blinding only.	Low risk – Leaflet was standardized, evidence-informed, piloted, and uniformly distributed; content tailored using validated message design methods.	Low risk – 1133 randomized, 972 included in analysis; missing data < 7%; ITT used; flowchart and exclusions reported transparently.	Low risk – Validated scales (Cancer Worry Scale, K6) applied consistently; survey-based; no differential measurement between arms; blinding unlikely to affect measurement.	Low risk – Outcomes pre-specified; statistical plan includes ORs with CIs by cytological grade; CONSORT reporting followed.	**Some concerns** – Minor concerns over randomization concealment; outcome assessment and analysis highly structured and transparent.	**Moderate certainty** – Evidence of leaflet efficacy in reducing distress; robust design and measurement but generalizability limited due to hypothetical results and localized sample.
**8**	Li et al., 2023, China	Randomized Controlled Trials	Low risk – Randomization done via computer sequence by third-party; allocation concealed; participants blinded to group prior to intervention; trial registered.	Low risk – Structured 8-week interventions; session content validated and described; fidelity ensured through actigraphy and group moderation via WeChat.	Some concerns – ITT applied but with 26.8% attrition; unclear impact of missingness at 3-month follow-up; not fully addressed missingness by group.	Low risk – Validated measures (HADS, PSQI, GSES, SSRS, IPAQ); objective biomarkers (cortisol, testosterone) used; consistent protocol timing (7–8 a.m. sample).	Low risk – Pre-specified outcomes; detailed statistical methods (LMM, ANOVA, Bonferroni corrections); multiple timepoints evaluated; reporting adheres to CONSORT.	**Some concerns** – Despite strong protocol and measures, high attrition and imbalance in data completion raise moderate bias risk.	**Moderate certainty** – Innovative dual-intervention RCT with physiological outcomes; strong internal methods; limited external validity due to single region and sample age band.
**9**	Lopez et al., 2023, USA	Randomized Controlled Trials	Low risk – Randomization via adaptive	Low risk – APP-based delivery with standardized	Low risk – 35 participants randomized;	Low risk – Validated instruments used	Low risk – Clearly defined primary/second	**Low risk** – Methodologica lly robust	**Moderate certainty** – Promising findings on meditation feasibility;

No.	ID	Study Design	RoB 2 D1 (Judgement/ Support)	RoB 2 D2 (Judgement/ Support)	RoB 2 D3 (Judgement/ Support)	RoB 2 D4 (Judgement/ Support)	RoB 2 D5 (Judgement/ Support)	Overall RoB 2 (Judgement/ Support)	GRADE Certainty (Judgement/ Support)
			minimization with allocation concealment; participant enrollment independent of assessment; trial registered.	meditation durations; intervention fidelity ensured through automated tracking and fixed instructional materials.	71% adherence; ITT applied; missing data minimal; pre-post design consistent across arms.	(ESAS-FS, HADS, PSQI); consistent timing and blinded outcome data collection; electronic entry ensured data fidelity.	ary outcomes; CONSORT diagram provided; pre-planned analyses using appropriate statistical models (LMM, Wilcoxon, t-tests).	design and reporting; clearly implemented protocol and minimal missing data; well-controlled despite small sample size.	however, limited generalizability due to pilot design, short duration, and predominantly female, breast cancer sample.
**10**	Manne et al., 2017, USA	Randomized Controlled Trials	Low risk – Stratified randomization using BDI scores; computer-generated; allocation concealed; randomization overseen across seven US sites.	Low risk – CCI and SC both manualized; 8-session structure; fidelity ratings and audiotaped sessions for compliance monitoring.	Some concerns – Moderate attrition over 18 months (24%); primary analyses used multilevel modeling which handles missingness, but some survey return imbalance noted.	Low risk – Validated instruments (BDI, IES, FACT-G, CARS); longitudinal multilevel modeling used; standard timepoints applied across groups.	Low risk – Pre-specified outcomes; MLM used with well-defined moderators and covariates; data reported in detail for each follow-up point.	Some concerns – Excellent intervention fidelity and pre-specification; however, moderate attrition over time introduces possible bias in long-term conclusions.	Moderate certainty – Strong design with multiple follow-ups; long-term effects less robust; highly generalizable within gynecological oncology but attrition and absence of sustained impact reduce certainty.
**11**	Marziliano et al., 2023, USA	Randomized Controlled Trials	Low risk – Computer-generated randomization; allocation	Low risk – Multimedia tool (Healing Choices) standardized with defined modules;	Low risk – ITT applied; moderate dropout (~37% from 617 to	Low risk – Psychological distress measured with validated IES-Intrusion;	Low risk – Pre-specified primary outcomes; regression	**Low risk** – Strong implementation and analysis rigor;	**Moderate certainty** – Well-structured multimedia intervention; results mixed due to limited

No.	ID	Study Design	RoB 2 D1 (Judgement/ Support)	RoB 2 D2 (Judgement/ Support)	RoB 2 D3 (Judgement/ Support)	RoB 2 D4 (Judgement/ Support)	RoB 2 D5 (Judgement/ Support)	Overall RoB 2 (Judgement/ Support)	GRADE Certainty (Judgement/ Support)
			described; intent-to-treat and as-treated analyses presented; trial registered.	support tools (letters, CD-ROM, online access) equally distributed; fidelity supported by user tracking.	388 analyzed); handled with subgroup (as-treated) analyses; engagement monitored.	outcome assessors blinded; high internal consistency (α = 0.82).	models detailed; both ITT and as-treated analyses transparently reported.	transparency in limitations; sample heterogeneity acknowledged.	usage and possible information overload; robust design enhances confidence in partial effects.
**12**	Nissen et al., 2020, Denmark	Randomized Controlled Trials	Low risk – Computerized block randomization stratified by cancer type; allocation sequence generated independently; research assistants were blinded to group allocation.	Low risk – 8-week iMBCT intervention with standardized modules; therapist feedback protocolized; participant adherence monitored; control group waitlisted; reminders standardized.	Some concerns – Higher attrition in intervention group (up to 31%) vs. control (≤20%); missing data imputed via zero-effect baseline (ZEFB) method; ITT applied.	Low risk – Primary outcomes assessed using validated STAI-Y and BDI-II; high internal consistency (α = .94 and .85); consistent timing across assessments.	Low risk – Pre-specified outcomes; detailed analysis plan using MLM; effect sizes calculated and reported transparently; CONSORT flowchart included.	**Some concerns** – While rigorous in most domains, differential attrition and imputation assumptions slightly limit confidence in final estimates.	**Moderate certainty** – Strong methodological rigor and follow-up design; effects on anxiety robust; limited power for prostate subgroup and small effect on depression reduce certainty.
**13**	Park et al., 2020, Japan	Randomized Controlled Trials	Low risk – Block randomization stratified by anxiety severity; computer-generated; sequence managed by an independent	Low risk – 8-week MBCT with fidelity ensured by therapist training, adherence checks, and structured protocol; no restrictions on cointerventions.	Low risk – Very low attrition (7.9%); ITT applied; missing data <5%; clearly reported with reasons;	Low risk – Validated scales used (HADS, CARS, BFI, FACIT-Sp, FACT-G); consistent timing; effect sizes and CI reported; blinded	Low risk – Pre-specified outcomes; repeated measures LMM used; comprehensive reporting of Cohen's d, CI,	**Low risk** – High methodological rigor across domains; minimal attrition and well-validated instruments	**Moderate certainty** – MBCT yielded strong short-term effects on distress, fatigue, QOL, and spirituality; limited by small sample and use of WLC instead of active control.

No.	ID	Study Design	RoB 2 D1 (Judgement/ Support)	RoB 2 D2 (Judgement/ Support)	RoB 2 D3 (Judgement/ Support)	RoB 2 D4 (Judgement/ Support)	RoB 2 D5 (Judgement/ Support)	Overall RoB 2 (Judgement/ Support)	GRADE Certainty (Judgement/ Support)
			center; clearly registered.		consistent attendance (~6.76 sessions).	outcome analysis implied.	and p-values; CONSORT diagram provided.	support internal validity.	
**14**	Rodin et al., 2018, Canada	Randomized Controlled Trials	Low risk – Permuted block randomization stratified by PHQ-9; computer-generated by independent biostatistics unit; allocation concealed; clearly preregistered.	Low risk – CALM delivered via manualized, structured, therapist-supervised format; fidelity monitored through audio recordings and rating scale assessments.	Some concerns – 25% attrition by 6 months due to death/loss; ITT used; mixed models and multiple imputation applied; death-related attrition largely non-differential.	Low risk – Primary and secondary outcomes assessed using validated measures (PHQ-9, GAD-7, DADDS, FACIT-Sp-12, QUAL-EC, etc.); consistent timepoints and independent assessors.	Low risk – Detailed reporting of analytic strategy including ANCOVA, mixed models, and FDR-adjusted sensitivity; clearly pre-specified outcomes and subgroup analysis.	**Some concerns** – Methodological strengths overall, though attrition from mortality introduces nontrivial limitations despite appropriate handling.	**Moderate certainty** – Significant benefit on depression and end-of-life prep; robust design; however, attrition, mortality, and single-center generalizability limit full certainty.
**15**	Ross et al., 2016, USA	Randomized Controlled Trials	Low risk – Randomized, double-blind crossover design; computer-generated assignment; sequence concealed; trial registered (NCT00957359).	Low risk – Manualized psychotherapy included in both arms; standard dosing (psilocybin or niacin); rigorous session procedures and supervision implemented.	Low risk – Very low attrition (minor dropouts only post-crossover); ITT applied; missing data minimal and handled by	Low risk – Validated measures used (HADS, BDI, STAI); consistent follow-up; outcome assessments blinded; Cohen's d and mediation analysis applied.	Low risk – Clearly pre-specified outcomes; comprehensive statistical reporting; mediation analysis conducted to	**Low risk** – Thorough design and follow-up; well-controlled crossover model; effective blinding; robust	**Moderate certainty** – Strong evidence of durable effect on anxiety/depression; generalizability limited by small, homogenous sample and active control limitations.

No.	ID	Study Design	RoB 2 D1 (Judgement/ Support)	RoB 2 D2 (Judgement/ Support)	RoB 2 D3 (Judgement/ Support)	RoB 2 D4 (Judgement/ Support)	RoB 2 D5 (Judgement/ Support)	Overall RoB 2 (Judgement/ Support)	GRADE Certainty (Judgement/ Support)
					mixed-effects modeling.		assess effect mechanisms.	statistical strategy increases confidence in results.	
		**INTERVENTION: Mindfulness and Stress Reduction**<br>Mindfulness-Based Stress Reduction (MBSR); Mindfulness-Based Cognitive Therapy (MBCT); Relaxation therapy and guided meditation							
**16**	Bagherzadeh et al., 2022, Iran	Randomized Controlled Trials	Low risk – Randomization using random number table; equal group size; sequence concealed; eligibility confirmed before assignment.	Low risk – MBSR delivered per Kabat-Zinn protocol; session fidelity via standardized structure and same facilitator; adherence monitored via notebooks and WhatsApp.	Some concerns – 6 participants dropped; analysis included 46 patients (22 vs. 24); missingness managed but limited long-term follow-up.	Low risk – Rumination assessed with validated Persian adaptation of Nolen-Hoeksema scale; Cronbach's $\alpha = 0.88$; same tool at all timepoints.	Low risk – Outcomes pre-specified; repeated-measures ANOVA and ANCOVA applied with pre-test as covariate; effect sizes shown; CONSORT diagram included.	**Some concerns** – Strong delivery and analysis; lack of long-term follow-up and dropout pattern create minor uncertainty.	**Moderate certainty** – Culturally specific MBSR application yielded effects on rumination; results constrained by short follow-up and small sample.
**17**	Bower et al., 2015, USA	Randomized Controlled Trials	Low risk – Randomized 2-arm trial (MAPS vs. WLC); block randomization with concealed envelopes; stratified enrollment and IRB	Low risk – Standardized 6-week MAPS intervention; session adherence high (87% attendance); fidelity monitored via home practice	Low risk – 92% post-treatment retention, 83% at 3-month follow-up; ITT and LMM applied; missingness	Low risk – Validated psychological and inflammatory biomarkers used (PSS, CES-D, IL-6, CRP, NF-κB, etc.); repeated assessments;	Low risk – Pre-specified outcomes; adjusted mixed model analysis; multiple comparisons controlled (Hommel	**Low risk** – Strong internal validity, low attrition, rigorous design and transparent statistical handling	**Moderate certainty** – Strong results on acute stress and inflammation; longer-term maintenance limited; small homogeneous sample and passive control limit generalizability.

No.	ID	Study Design	RoB 2 D1 (Judgement/ Support)	RoB 2 D2 (Judgement/ Support)	RoB 2 D3 (Judgement/ Support)	RoB 2 D4 (Judgement/ Support)	RoB 2 D5 (Judgement/ Support)	Overall RoB 2 (Judgement/ Support)	GRADE Certainty (Judgement/ Support)
			approval; preregistered.	reports and consistent delivery by trained instructors.	handled appropriately; baseline equivalence confirmed.	blinding implied; consistent tools at all timepoints.	correction); transcriptional profiling transparent and reproducible.	across domains.	
**18**	Cillessen et al., 2018, Netherlands	Randomized Controlled Trials	Low risk – Multicenter 3-arm RCT; computer-generated randomization; concealed stratified assignment; preregistered OSF protocol; IRB approved.	Low risk – MBCT and eMBCT manualized from Segal et al.; fidelity ensured by supervision, therapist certification, and MBI-TAC ratings; consistent delivery confirmed.	Some concerns – ~25–30% missing data at T3; not MCAR; MAR assumption used with multiple imputation; sensitivity analyses conducted but differential dropout noted.	Low risk – HADS, FCRI, RRQ, MHC-SF, SF-12 validated tools applied; consistent intervals; Cronbach α reported; assessments standardized across timepoints.	Low risk – Pre-registered outcomes; prespecified moderator/predictor models; detailed regression/interaction analyses; residual change scores applied.	**Some concerns** – High design quality with strong standardization; dropout imbalance and MAR reliance introduce mild residual uncertainty.	**Moderate certainty** – Longitudinal data confirm MBCT/eMBCT efficacy; online version slightly superior; dropout bias and generalizability limitations reduce certainty.
**19**	Duval et al., 2022, Canada	Randomized Controlled Trials	Low risk – Randomized waitlist-controlled design; stratified permuted block randomization (block sizes 2/4/6); allocation concealed; IRB approved and preregistered.	Low risk – Standard MBSR protocol with expert facilitator; session adherence monitored; fidelity via structured delivery; 8 weekly sessions + retreat.	Some concerns – Limited info on missing data strategy; ~30% of control group opted into MBSR after 3-month mark; effects not sustained	Low risk – CNS-VS, FACT-Cog, and PRMQ tools validated; high internal consistency (Cronbach α > .90); consistent assessments across three timepoints.	Low risk – Outcomes clearly defined; mixed models with covariates used; baseline equivalence confirmed; prespecified cognitive endpoints.	**Some concerns** – Strong intervention quality, but cross-over contamination and uncertain dropout handling	**Moderate certainty** – Significant short-term memory benefits (PRMQ), but effects not durable; objective tests (CNS-VS) showed no between-group effects; CNP comorbidity limits generalizability.

No.	ID	Study Design	RoB 2 D1 (Judgement/ Support)	RoB 2 D2 (Judgement/ Support)	RoB 2 D3 (Judgement/ Support)	RoB 2 D4 (Judgement/ Support)	RoB 2 D5 (Judgement/ Support)	Overall RoB 2 (Judgement/ Support)	GRADE Certainty (Judgement/ Support)
					long-term; dropout analysis not detailed.			reduce certainty.	
**20**	Gu et al., 2024, China	Randomized Controlled Trials	Low risk – Random allocation via sealed envelopes and random number generator; stratified assignment; preregistered; IRB approved.	Low risk – 8-week online MBSR standardized from Kabat-Zinn; fidelity via video/manual delivery and WeChat monitoring; informal and formal practices logged.	Some concerns – Dropout not fully characterized; 78/102 completed; no imputation; follow-up at 3 and 6 months but with differential attrition; MCAR not evaluated.	Low risk – All scales validated (CFS, MUIS, MCMQ, MSPSS, SOC-13); Cronbach $\alpha > 0.7$–0.8; consistent timing; tools reliable and appropriate.	Low risk – Repeated-measures ANOVA and Bonferroni corrections applied; primary outcomes clearly defined and statistically analyzed; effect sizes reported.	**Some concerns** – Strong methodology but incomplete reporting of dropout and attrition mechanism slightly lowers confidence.	**Moderate certainty** – CRF and SOC improvements maintained up to 6 months; uncertainty in illness rebounded post-intervention; PSS showed no significant change.
**21**	Johns et al., 2015, USA	Randomized Controlled Trials	Low risk – 1:1 randomized pilot RCT with concealed assignment via sealed envelopes; block randomization used; IRB-approved; preregistered.	Low risk – MBSR-CRF intervention adapted from Kabat-Zinn; high adherence (90% attendance); formal/informal home practice logged; experienced instructor blinded to logs/outcomes.	Low risk – Minimal attrition; 34/35 completed 6-month follow-up; no imputation needed; adherence and retention were high; T1 covariates	Low risk – Validated tools (FSI, PHQ-8, GAD-7, SDS, ISI, SF-36); Cronbach α reported; self-report standardized; consistent timepoints and tools across groups.	Low risk – Bonferroni correction for 18 tests; ANCOVA applied with T1 adjustment; primary and secondary outcomes clearly pre-defined; effect sizes calculated.	**Low risk** – High-quality design, robust statistical control, excellent retention, strong outcome reporting with proper correction for	**Moderate certainty** – Strong effect sizes sustained at 6 months; generalizability limited by small, homogeneous sample and absence of active control.

No.	ID	Study Design	RoB 2 D1 (Judgement/ Support)	RoB 2 D2 (Judgement/ Support)	RoB 2 D3 (Judgement/ Support)	RoB 2 D4 (Judgement/ Support)	RoB 2 D5 (Judgement/ Support)	Overall RoB 2 (Judgement/ Support)	GRADE Certainty (Judgement/ Support)
					adjusted in analysis.			multiple comparisons.	
**22**	Johns et al., 2016, USA	Randomized Controlled Trials	Low risk – 1:1 randomization with block sizes, concealed allocation via opaque sealed envelopes; preregistered; IRB-approved; eligibility rigorously defined.	Low risk – Fidelity ensured by manualized 8-week MBSR and ES protocols; sessions audio-recorded, reviewed; logs tracked practice; instructor blinded to logs.	Low risk – Retention at T2 = 97%, T3 = 94%; intent-to-treat used; minimal missingness; imputation applied; no systematic bias detected in dropout pattern.	Low risk – AFI, Stroop, FFMQ used; validated with reported Cronbach α (AFI = 0.87–0.90); assessments consistent; measures psychometrically strong.	Low risk – ANCOVA used for group differences; bootstrapped mediation models; clearly defined endpoints; correction for multiple comparisons; robust statistical approach.	**Low risk** – Excellent design and analytical rigor; high retention and valid tools; appropriate handling of dropouts and statistical analysis.	**Moderate certainty** – Strong within- and between-group cognitive improvements maintained at 6 months; generalizability limited by small sample and focus on fatigued survivors.
**23**	Kenne et al., 2017, Sweden	Randomized Controlled Trials	Low risk – Randomized three-arm design using concealed envelopes; block randomization; IRB-approved; preregistered; baseline equivalence ensured.	Low risk – Manualized 8-week MBSR with instructor vs. self-instructing and control; adherence tracked via diaries and CD/audio logs; fidelity monitored.	Low risk – Minimal attrition (6.2%); dropout reasons reported; data collected from 165/177 participants; statistical analysis used ITT and Wilcoxon/Man	Low risk – Validated tools (HAD, MSAS, SOC, FFMQ, PTGI); biological markers (NK cell activity, CD lymphocytes, cytokines) used; Cronbach α reported (0.72–0.96).	Low risk – All primary and secondary outcomes pre-specified; multiple comparisons accounted; Mann-Whitney and within-group Wilcoxon tests applied; effect sizes reported.	**Low risk** – Comprehensive and rigorous methodology; randomized controlled three-arm design with active and passive comparators; biological and psychological integration.	**Moderate certainty** – Strong design and dual psychological/biological outcomes; limited by short follow-up and moderate clinical generalizability.

No.	ID	Study Design	RoB 2 D1 (Judgement/ Support)	RoB 2 D2 (Judgement/ Support)	RoB 2 D3 (Judgement/ Support)	RoB 2 D4 (Judgement/ Support)	RoB 2 D5 (Judgement/ Support)	Overall RoB 2 (Judgement/ Support)	GRADE Certainty (Judgement/ Support)
					n-Whitney nonparametric tests.				
**24**	Lengacher et al., 2016, USA	Randomized Controlled Trials	Low risk – Large N (n=322), stratified block randomization with 1:1 allocation; preregistered; IRB-approved; baseline covariate equivalence confirmed.	Low risk – MBSR(BC) adapted from Kabat-Zinn's 8-week protocol; structured 6-week delivery by trained psychologists; fidelity ensured via session observation and diary reviews.	Low risk – 9% attrition; intent-to-treat applied; linear mixed models using full-information maximum-likelihood; missing at random assumption justified.	Low risk – Validated measures (CES-D, STAI-S, FSI, CARS, SF-36); Cronbach α values reported; consistent timing; biological and self-report integration.	Low risk – Primary/secondary outcomes prespecified; mixed models used; sensitivity analysis with unstructured correlation structure conducted; moderator analyses explored.	**Low risk** – Robust statistical design, excellent retention, transparent handling of outcomes and dropouts; analytical rigor across all phases.	**High certainty** – Largest RCT of MBSR(BC) to date; significant symptom improvements sustained over 12 weeks.
**25**	Mirmahmoodi et al., 2020, Iran	Randomized Controlled Trials	Low risk – Parallel RCT; block randomization (size 4) via sealed envelopes; allocation list generated by third party using online randomizer; registered; IRB approved.	Low risk – Manualized 8-session MBSR protocol with group delivery; structured training, standardized scripts, and trained facilitator; session content explicitly described; adherence ensured.	Some concerns – Small sample (n=44); no imputation strategy described; dropout prevention attempted via exclusion criteria and monitoring; some	Low risk – Validated Persian versions of BAI, BDI-II, and PSS; appropriate lab tests for CRP and cortisol; repeated measures design; high internal consistency reported for tools.	Low risk – ANCOVA, t-tests, and Wilcoxon applied; mean differences and significance reported; outcomes explicitly predefined in protocol; appropriate	**Some concerns** – Methodologically sound and transparent, but limited by small sample, no imputation for missing data, and baseline imbalance.	**Moderate certainty** – Clear reduction in anxiety, no effect on depression, stress, or biomarkers; small sample limits generalizability despite solid protocol and measurements.

No.	ID	Study Design	RoB 2 D1 (Judgement/ Support)	RoB 2 D2 (Judgement/ Support)	RoB 2 D3 (Judgement/ Support)	RoB 2 D4 (Judgement/ Support)	RoB 2 D5 (Judgement/ Support)	Overall RoB 2 (Judgement/ Support)	GRADE Certainty (Judgement/ Support)
					imbalance in pre-test psychological scores.		stats for covariates used.		
**26**	Reich et al., 2017, USA	Randomized Controlled Trials	Low risk – 2-arm RCT, stratified by surgery type, stage, and treatment; preregistered; IRB-approved; randomization and allocation details well documented.	Low risk – 6-week MBSR(BC) manualized and delivered by trained psychologist; fidelity checklist used; adherence monitored through diaries; participants refrained from MBSR practice in UC group.	Low risk – High retention (93%); ITT analysis; Full Information Maximum Likelihood estimation; MAR assumption justified; outcome completeness documented at all points.	Low risk – Broad psychometric battery (CES-D, STAI, PSS, CAMS-R, ECog); high internal consistency (α = 0.74–0.95); validated measures for each symptom domain; appropriate frequency.	Low risk – All outcomes predefined; structural equation modeling applied; effect sizes and latent variables tested; robust model fit indices (CFI > 0.95, RMSEA < 0.07, SRMR < 0.08).	**Low risk** – Strong design, large N, robust SEM and clustering methods, comprehensive outcome measurement, and good retention rates support low overall risk.	**Moderate certainty** – Demonstrated medium effect on psychological and fatigue symptom clusters; improvements sustained to 12 weeks; generalizability limited to BCS population.
**27**	Shergill et al., 2022, Canada	Randomized Controlled Trials	Low risk – RCT with stratified, permuted block randomization; preregistered; allocation concealed; IRB-approved; robust stratification on	Low risk – Manualized 8-week MBSR protocol by certified clinicians with 5+ years of experience; consistent format and structure across sites; adherence and fidelity rated via	Some concerns – Missing data on primary outcome for 20%+ participants; no imputation strategy described; intent-to-treat implied but	Low risk – Validated tools (BPI, PHQ-9, PCS, FFMQ, SF-12, POMS, NPSI); Cronbach α consistently reported (>0.85); multiple timepoint	Low risk – Predefined outcomes and stratification; mixed effects regression and SEM used; sensitivity analyses for missing data; effect sizes and	**Some concerns** – Rigorous design and implementation, but attrition and missing data weaken overall reliability despite strong	**Moderate certainty** – No statistically significant between-group differences in primary or secondary outcomes; exploratory subgroup data indicate trends; study was underpowered for firm conclusions.

No.	ID	Study Design	RoB 2 D1 (Judgement/ Support)	RoB 2 D2 (Judgement/ Support)	RoB 2 D3 (Judgement/ Support)	RoB 2 D4 (Judgement/ Support)	RoB 2 D5 (Judgement/ Support)	Overall RoB 2 (Judgement/ Support)	GRADE Certainty (Judgement/ Support)
			pain etiology/severity.	MBI:TAC with average 5.49 score (proficient).	not clearly stated; high attrition (24%).	assessment; good psychometrics.	confidence intervals reported.	analytic framework.	
**28**	Victorson et al., 2020, USA	Randomized Controlled Trials	Low risk – RCT with waitlist control, 1:1 allocation via computerized randomization; preregistered; IRB-approved; baseline comparability examined.	Low risk – Manualized 8-week MBSR delivered by trained instructors with weekly sessions and retreat; adherence monitored; fidelity ensured via recording and supervision.	Some concerns – 25% dropout post-randomization ; no imputation strategy stated; attrition due to time/health burdens; analyses adjusted for attendance and baseline disparities.	Low risk – Validated instruments (PROMIS CATs, MAAS, SCS, PTGI-SF); good psychometrics; assessments at 3 timepoints; measures described in full.	Low risk – Mixed models with covariate adjustments; outcomes predefined and well described; Cohen's d and significance reported; within and between group contrasts tested.	**Some concerns** – Pilot trial with solid structure and fidelity, but retention limitations and missing data decrease confidence in estimates.	**Moderate certainty** – Pilot findings show improvements in self-kindness, sleep, and PTG; underpowered for robust between-group comparisons; valuable for feasibility insights.
			**INTERVENTION TYPE: Coping and Psychological Resilience**<br>PRISM (Promoting Resilience in Stress Management); Coping skills training; Therapeutic journaling, art therapy, or narrative therapy (if validated and quantifiable)						
**29**	Cafaro et al., 2024 Italy	Randomized Controlled Trials	Low risk – Centralized computer-generated randomization, allocation concealment via phone; preregistered trial;	Low risk – Manualized GDP and control writing protocol; session reminders and standardization by study coordinator; consistent	Some concerns – Moderate dropout at T1 and T2 (21 total dropouts); no imputation strategy for missing data	Low risk – Validated scales (PTGI, CMS, IES, HADS); Italian versions with prior psychometric validation; scale properties and	Low risk – Outcomes predefined; primary endpoint clearly stated; statistical plan detailed including ANOVA and t-	**Some concerns** – Despite robust design, moderate attrition and lack of imputation strategy lower	**Moderate certainty** – Underpowered sample; no statistically significant differences in outcomes; study shows trends in favor of GDP; replication recommended.

No.	ID	Study Design	RoB 2 D1 (Judgement/ Support)	RoB 2 D2 (Judgement/ Support)	RoB 2 D3 (Judgement/ Support)	RoB 2 D4 (Judgement/ Support)	RoB 2 D5 (Judgement/ Support)	Overall RoB 2 (Judgement/ Support)	GRADE Certainty (Judgement/ Support)
			approved by multiple ethics committees.	implementation across centers.	described; dropout not always explained.	reliability reported in protocol and prior studies.	tests with 95% CIs and p-values.	confidence in findings.	
**30**	Cheung et al., 2017, USA	Randomized Controlled Trials	Low risk – Simple randomization to three arms; schedule generated by blinded statistician; allocation by independent project director; IRB approved and clearly described.	Low risk – LILAC intervention manualized; delivered in-person or online; control group was attention-matched; trained facilitators with fidelity monitoring via audiotapes.	Some concerns – 4 deaths and moderate attrition (77% retained at 1-month); no imputation strategy detailed; causes of dropout reported; retention balanced across groups.	Low risk – Validated tools (CES-D, Differential Emotions Scale, SCS-SF, MQoL-C); internal consistency $\alpha >$ .74; measurements at 3 timepoints with appropriate spacing.	Low risk – Outcomes prespecified; analyses conducted using longitudinal growth models; contrasts and effect sizes reported; marginal trends noted transparently.	**Some concerns** – High design fidelity and measurement, but small sample and limited statistical power impact certainty of findings.	**Moderate certainty** – Pilot trial with trends favoring intervention in depressive symptoms and negative affect; underpowered for detecting between-group differences.
**31**	Graboyes et al., 2023, USA	Randomized Controlled Trials	Low risk – 1:1 randomization using permuted blocks (sizes 4 or 6); allocation by project director; CONSORT-aligned; trial registered; IRB approved.	Low risk – Manualized BRIGHT CBT and AC protocols; 5 weekly 60-min telehealth sessions; licensed psychologists; dose and delivery matched; fidelity described.	Low risk – Minimal missing data; one imputed BICSI item; dropout well-documented and causes reported; analyses adjusted for	Low risk – Validated IMAGE-HN, Body Image Scale, BICSI subscales; psychometric evidence provided; repeated measures at	Low risk – Primary outcomes predefined; causal mediation analysis with multiple baselines; ITT approach; all pathways and	**Low risk** – Excellent design, fidelity, reporting, measurement, and statistical rigor in this pilot trial despite small sample size.	**Moderate certainty** – Rigorous mechanistic pilot RCT with validated outcomes and sound mediation analysis; limited by small sample and short follow-up.

No.	ID	Study Design	RoB 2 D1 (Judgement/ Support)	RoB 2 D2 (Judgement/ Support)	RoB 2 D3 (Judgement/ Support)	RoB 2 D4 (Judgement/ Support)	RoB 2 D5 (Judgement/ Support)	Overall RoB 2 (Judgement/ Support)	GRADE Certainty (Judgement/ Support)
					baseline scores.	multiple timepoints.	models statistically specified.		
**32**	Jensen-Johansen et al., 2018, Denmark	Randomized Controlled Trials	Low risk – Computerized stratified randomization by adjuvant protocol; group concealment maintained; nationwide RCT; CONSORT-compliant; ethics-approved.	Low risk – Structured Pennebaker EWI protocol; weekly 20-min sessions over 3 weeks; telephone-based facilitation standardized; manipulation check confirmed mood impact.	Some concerns – 17.2% total dropout; dropout associated with age and baseline depression; no imputation used; mixed model ITT analyses adjusted but not robustly explained.	Low risk – PHQ-15, GP-visit metrics, and validated Danish versions of TAS-20, ECQ, and BDI-SF used; strong psychometrics (α = 0.63–0.90); manipulation checks included.	Low risk – A priori outcomes and moderators; stratified and moderator subgroup analyses; detailed MLM models; sample size justified by power analysis.	**Some concerns** – Generally rigorous trial but notable dropout and lack of imputation lower confidence; selective benefits for specific subgroups suggest heterogeneity.	**Moderate certainty** – No group effects on outcomes overall, but relevant moderator effects detected (e.g., alexithymia, writing topic); findings hypothesis-consistent but modest.
**33**	Lu et al., 2023, USA	Randomized Controlled Trials	Low risk – 1:1:1 randomization with balanced baseline; preregistered; stratified by site; IRB-approved; CONSORT-compliant.	Low risk – Culturally adapted expressive writing (EW) intervention using SMEW model; three structured prompts delivered weekly; protocol co-designed with community	Low risk – ITT analysis conducted; missingness tested MAR; EM imputation applied; dropout 15.4% balanced across arms;	Low risk – Validated CES-D (α=.92), BSI-Anxiety (α=.96), PSS, and IES-Intrusion used; bilingual Chinese versions with strong psychometrics; LIWC used for	Low risk – Hypothesis-driven primary and secondary outcomes; mediation via PROCESS bootstrapping; significant indirect effects for stress as	**Low risk** – Robust culturally adapted RCT with multiple follow-ups, validated measures, and rigorous analysis including	**Moderate certain**ty – Strong theoretical model (SMEW), consistent benefits for ESR over time; limited by sample specificity (CABCS) and moderate attrition.

No.	ID	Study Design	RoB 2 D1 (Judgement/ Support)	RoB 2 D2 (Judgement/ Support)	RoB 2 D3 (Judgement/ Support)	RoB 2 D4 (Judgement/ Support)	RoB 2 D5 (Judgement/ Support)	Overall RoB 2 (Judgement/ Support)	GRADE Certainty (Judgement/ Support)
				stakeholders; fidelity maintained.	no baseline group differences in completers vs. non-completers.	emotional content analysis.	mediator; SR vs ESR comparisons conducted.	mediation and emotion word analysis.	
**34**	Nairn and Merluzzi, 2019, USA	Randomized Controlled Trials	Low risk – 1:1 randomization from pre-established allocation list; baseline equivalence confirmed; treatment fidelity high; IRB-approved; CONSORT diagram provided.	Low risk – Manualized 4-session Mastery Enhancement Therapy; based on self-regulation and self-efficacy theories; delivered by trained MA-level therapists; session protocol rigorously followed (91% fidelity).	Some concerns – 12.7% dropout and 14.9% death-related attrition; intention-to-treat and multilevel modeling used; dropout group differed at baseline on coping self-efficacy and stressors.	Low risk – Validated instruments used (CBI, BDI-BF, FACT-G, PAIS-SR, SII); high internal consistency (CBI α=.96, FACT-G α=.90, PAIS-SR α=.91); strong outcome-target fit.	Low risk – Clear primary outcome (CBI); predefined hypotheses and moderators; robust statistical modeling including 3-way interactions and effect sizes.	**Some concerns** – Pilot design with small sample and differential attrition in treatment group; otherwise rigorous methods and fidelity support internal validity.	**Moderate certainty** – Promising pilot RCT with brief, well-theorized intervention showing early efficacy on coping and moderator-sensitive gains in depression/adjustment; generalizability limited.
**35**	Nelson et al., 2021, USA	Randomized Controlled Trials	Low risk – 1:1 stratified randomization (by site and disease status); baseline characteristics balanced; trial preregistered; IRB-	Low risk – Manualized Integrated Palliative Care (IPC) intervention; bi-weekly inpatient sessions with standardized focus	Low risk – Follow-up at 5 timepoints; 68.1% enrollment rate; missing data addressed via	Low risk – Validated tools: Brief COPE (coping), FACT-Leukemia (QOL), HADS (anxiety/depression); bilingual	Low risk – All outcomes prespecified; mediation models specified a priori; PROCESS and	**Low risk** – Strong methodology and analysis, rigorous design, thorough mediation	**Moderate certainty** – Mediational analysis shows robust indirect effects for QOL and mood; limited by homogeneous AML population and unblinded design.

No.	ID	Study Design	RoB 2 D1 (Judgement/ Support)	RoB 2 D2 (Judgement/ Support)	RoB 2 D3 (Judgement/ Support)	RoB 2 D4 (Judgement/ Support)	RoB 2 D5 (Judgement/ Support)	Overall RoB 2 (Judgement/ Support)	GRADE Certainty (Judgement/ Support)
			approved; multicenter design with high external validity.	on symptom management, coping, decision-making; intervention fidelity monitored.	linear mixed effects models with maximum likelihood estimation; group balance and attrition analyzed.	where needed; high internal consistency; psychometric properties confirmed.	bootstrapping for indirect effects; CIs reported; effect decomposition robust.	modeling, and well-executed longitudinal follow-up.	
**36**	Rosenberg et al., 2018, USA	Randomized Controlled Trials	Low risk – Randomized (1:1) using permuted blocks stratified by age group (12–17 vs 18–25); baseline characteristics well balanced; trial registered (NCT02340884); IRB-approved.	Low risk – PRISM was a manualized, brief, 4-session intervention based on resilience theory; delivered by trained nonclinical staff; >90% completed full protocol; fidelity audio-monitored.	Low risk – Follow-up at 6 months; 92% completed baseline; attrition due to medical complications/ death; no significant imbalance between groups; sensitivity analyses confirmed robustness.	Low risk – Validated outcome tools (CD-RISC-10, Kessler-6, HADS, PedsQL); MCID estimated; psychometric evidence strong; outcome measures developmentally appropriate.	Low risk – Primary and secondary outcomes predefined; linear and logistic mixed models applied; effect sizes and MCIDs calculated; exploratory trajectories plotted.	**Low risk** – High-quality, developmentally tailored RCT with validated measures, strong fidelity, and transparent analyses supports internal validity.	**Moderate certainty** – Statistically and clinically significant improvements in resilience and distress; limited by single-center design and unblinded delivery.
**37**	Rosenberg et al., 2021, USA	Randomized Controlled Trials	Low risk – 1:1 randomization stratified by age (13–17 vs 18–25);	Low risk – PRISM intervention targeted stress management, goal	Low risk – ITT analysis; dropout mostly due to	Low risk – Validated, age-appropriate instruments:	Low risk – Outcomes predefined; repeated	**Low risk** – Strong follow-up design with robust	**Moderate certainty** – PRISM recipients showed durable gains in QoL and hope;

No.	ID	Study Design	RoB 2 D1 (Judgement/ Support)	RoB 2 D2 (Judgement/ Support)	RoB 2 D3 (Judgement/ Support)	RoB 2 D4 (Judgement/ Support)	RoB 2 D5 (Judgement/ Support)	Overall RoB 2 (Judgement/ Support)	GRADE Certainty (Judgement/ Support)
			permuted blocks; baseline balance confirmed; IRB-approved; trial registered (NCT02340884); CONSORT-compliant.	setting, cognitive reframing, and meaning-making; delivered by trained coaches; fidelity ensured via standardized training and protocol adherence.	medical complications or death (not related to intervention); missingness not at random (NMAR) acknowledged; survey follow-up robust at 6, 12, and 24 months.	PedsQL, Hope Scale, CDRISC-10, Kessler-6; psychometrics confirmed; MCID defined; R software used for analysis.	measures via mixed effects models; Kenward-Roger approximation used; significant interactions identified and explored.	modeling, valid outcomes, and well-handled missing data; high methodological rigor.	distress and resilience improvements were non-significant long-term; limited by attrition and single-site design.
**38**	Samami et al., 2021, Iran	Randomized Controlled Trials	Low risk – Permuted block randomization; balanced baseline; allocation concealment with sealed envelopes; approved by Ethics Committee and trial registered.	Low risk – Structured 6-week supportive program based on validated protocol; included stress management, relaxation, and coping strategies; delivered consistently by trained staff; high intervention fidelity.	Low risk – Three participants lost (intervention arm); ITT used; reasons for dropout stated (medical issues); GEE used for longitudinal analysis with robust	Low risk – Validated tools (WOCQ, DASS-21, MOS-SSS); reliability and construct validity confirmed in Iranian population; outcome measures psychometrically strong.	Low risk – Predefined outcomes and timepoints; repeated-measures tests and GEE modeling applied; clear statistical methodology and effect sizes reported.	**Low risk** – Methodologically rigorous RCT with strong design, delivery, follow-up, and valid outcome measures.	**Moderate certainty** – Strong within- and between-group effects on coping and stress; limited by unblinded design and short follow-up duration (1 month).

No.	ID	Study Design	RoB 2 D1 (Judgement/ Support)	RoB 2 D2 (Judgement/ Support)	RoB 2 D3 (Judgement/ Support)	RoB 2 D4 (Judgement/ Support)	RoB 2 D5 (Judgement/ Support)	Overall RoB 2 (Judgement/ Support)	GRADE Certainty (Judgement/ Support)
					handling of attrition.				
**39**	Santoyo-Olsson et al., 2022, USA	Randomized Controlled Trials	Low risk – Participants randomized using computer-generated permuted block sequence; stratification by time since diagnosis; allocation concealed; ethics approved (UCSF IRB); trial registered.	Low risk – Peer-delivered, culturally tailored cognitive-behavioral stress management (Nuevo Amanecer) intervention; fidelity maintained through standardized manuals and bilingual staff.	Low risk – Baseline-only analysis reported; high completion of pre-intervention surveys; missingness addressed; robust psychometric data.	Low risk – Validated Spanish instruments (e.g., BSI-Anxiety, Health Distress Scale, MOS-SSS); high reliability (Cronbach $\alpha >$ 0.85); measures culturally adapted and tested in pilot.	Low risk – Outcomes and predictors prespecified; hierarchical regression used; model variance and significance reported for each step; detailed handling of covariates and scales.	**Low risk** – Strong methodology, rigorous cultural tailoring, and thorough psychometric support ensure high internal validity.	**Moderate certainty** – Strong associations identified for intrapersonal and interpersonal resources; limitations include cross-sectional design and generalizability restricted to low-income Spanish-speaking Latinas.
**40**	Tutino et al., 2022, USA	Randomized Controlled Trials	High risk – Participants were not randomized; comparison used a historical control (ESWC); differences in recruitment periods may introduce selection bias; no allocation concealment.	Low risk – CARE-Express intervention structured across 5 sessions using trained writing mentors; fidelity high (97%); content aligned with the developmental and coping frameworks.	Some concerns – Retention high (90%), but only 70% and 63% completed post and 4-month assessments, respectively; potential bias from missing data; no	Low risk – Validated scales (HADS, Demoralization, FACIT-Sp-12, COPE, UCLA Loneliness); internal consistency strong; tools developmentally and clinically appropriate.	Some concerns – Outcomes not preregistered as primary/secondary distinctions; statistical models appropriate but exploratory effect size focus; p-values mostly non-significant.	**High risk** – Lack of randomization and use of historical control introduce substantial bias risk despite strong intervention and analysis design.	**Low certainty** – Pilot data showed trends favoring CARE-Express on depression, demoralization, and spirituality, but limited by design, sample homogeneity, and power.

No.	ID	Study Design	RoB 2 D1 (Judgement/ Support)	RoB 2 D2 (Judgement/ Support)	RoB 2 D3 (Judgement/ Support)	RoB 2 D4 (Judgement/ Support)	RoB 2 D5 (Judgement/ Support)	Overall RoB 2 (Judgement/ Support)	GRADE Certainty (Judgement/ Support)
					imputation reported.				
**41**	Winger et al., 2023, USA	Randomized Controlled Trials	Low risk – Parallel RCT with 1:1 allocation using REDCap system; sequence generated independently; baseline groups equivalent; IRB-approved and trial preregistered.	Low risk – Manualized 4-session MCPC intervention via telehealth; therapist trained and supervised; fidelity high (mean adherence=4.87/5); 93% completed all sessions.	Low risk – Attrition ≤25% at 5- and 10-weeks; analyses followed ITT; mixed models applied assuming MAR; no imbalance in dropout between groups.	Low risk – Validated outcome measures (BPI, PROMIS, Self-Efficacy, FACIT-Sp, GAD-7, PHQ-8, HAIQ); Cronbach α = 0.72–0.93; tools psychometrically validated for cancer patients.	Low risk – Predefined outcomes; effect sizes (Cohen's d) and 95% CIs reported; significance testing avoided per pilot guidelines; PROC MIXED used appropriately.	**Low risk** – Excellent implementation, remote delivery feasibility, strong therapist fidelity, and valid measurement support robust trial design.	**Moderate certainty** – Moderate-to-large improvements in pain outcomes and self-efficacy; limited by pilot scale and homogenous, well-resourced sample.
**42**	Wittmann et al., 2022, USA	Randomized Controlled Trials	Low risk – Centralized computer-generated 1:1 randomization with block sizes; allocation concealed; multi-center trial; stratified by site; trial registered.	Low risk – TrueNTH Sexual Recovery Intervention was modular, online, tailored by treatment and sexual orientation; validated content based on conceptual model and pilot-tested; high user	Some concerns – 46% attrition at 6 months limits interpretability; mixed-model ANCOVA used; baseline equivalence supported; intention-to-treat for available data.	Low risk – Validated PROMIS GSSL, FSFI, IIEF, EPIC-26, Sexual Interest scales; psychometrically strong tools with adequate internal consistency; baseline and follow-up assessments.	Low risk – Prespecified primary and secondary outcomes; adjusted comparisons using t-tests and ANCOVA; effect sizes and 95% CIs reported for PROMIS GSSL and sexual	**Some concerns** – Strong intervention design and psychometrics; high attrition reduces confidence in outcome validity and GRADE certainty.	**Low certainty** – PROMIS GSSL unchanged; improved sexual activity at 3 months in intervention group; positive user feedback; limited by attrition, modest effects, and homogeneity.

No.	ID	Study Design	RoB 2 D1 (Judgement/ Support)	RoB 2 D2 (Judgement/ Support)	RoB 2 D3 (Judgement/ Support)	RoB 2 D4 (Judgement/ Support)	RoB 2 D5 (Judgement/ Support)	Overall RoB 2 (Judgement/ Support)	GRADE Certainty (Judgement/ Support)
				engagement early in trial.			activity outcomes.		

Notes:

- **AAQ-II:** Acceptance and Action Questionnaire-II (experiential avoidance).
- **AC**: Attention Control – dose-matched survivorship education without behavior change mechanisms
- **ACT: Acceptance and Commitment Therapy** – a third-wave behavioral therapy that encourages psychological flexibility by fostering acceptance of unpleasant experiences and commitment to value-based action.
- **ACT-FM (ACT Fidelity Measure):** Evaluates therapist adherence to ACT principles.
- **ACTION trial**: A co-designed ACT-based intervention to support adherence to endocrine therapy in breast cancer; includes one individual and three group sessions plus web-based components.
- **Active control group**: A comparator arm that receives an intervention (non-specific group discussion) rather than a placebo or no treatment, reducing expectancy bias but possibly minimizing detectable differences.
- **AFI: Attentional Function Index** – evaluates perceived cognitive effectiveness in daily activities.
- **AML**: Acute Myeloid Leukemia.
- **ANCOVA:** Analysis of Covariance – A statistical method adjusting outcome differences for baseline values.
- **ANOVA (Analysis of Variance):** A statistical method used to compare means across multiple groups and determine whether any of those group means are statistically significantly different from each other. In this study, repeated-measures ANOVA was used to assess group × time interactions for psychological outcomes.
- **APP: Application** – mobile or computer-based program for intervention delivery and participant tracking.
- **ASK-12 (Adherence Starts with Knowledge questionnaire):** A validated tool measuring adherence behavior, beliefs, and barriers.
- **BA: Behavioral Activation** – a structured psychotherapy that encourages engagement in meaningful activities to counter depressive and anxiety symptoms.
- **BADS-A**: Behavioral Activation for Depression Scale – Activation subscale; evaluates engagement in positive behavior.
- **BAI:** Beck Anxiety Inventory – measures severity of anxiety symptoms.
- **BDI: Beck Depression Inventory** – 21-item scale measuring depressive symptoms severity.
- **BDI-II: Beck Depression Inventory-II** – a validated measure of depressive symptoms.
- **BFI: Brief Fatigue Inventory** – assesses fatigue severity and impact.
- **BICSI**: Body Image Coping Skills Inventory – Avoidance, Appearance Fixing, Rational Acceptance
- **BID**: Body Image Distress.
- **Bonferroni:** Statistical correction method for multiple comparisons.
- **Bonferroni Correction:** Adjustment for multiple comparisons to control Type I error.
- **BPI:** Brief Pain Inventory – pain intensity and interference.
- **BRCA: BRCA1/2** – breast and ovarian cancer susceptibility genes.
- **BRIGHT:** Building a Renewed ImaGe after Head & neck cancer Treatment.
- **BSI**: Brief Symptom Inventory.

No.	ID	Study Design	RoB 2 D1 (Judgement/ Support)	RoB 2 D2 (Judgement/ Support)	RoB 2 D3 (Judgement/ Support)	RoB 2 D4 (Judgement/ Support)	RoB 2 D5 (Judgement/ Support)	Overall RoB 2 (Judgement/ Support)	GRADE Certainty (Judgement/ Support)

- **CABCS**: Chinese American Breast Cancer Survivors
- **CALM: Managing Cancer and Living Meaningfully** – a manualized supportive-existential psychotherapy developed for patients with advanced cancer, addressing emotional distress, existential concerns, and adaptation to illness.
- **CAMS-R**: Cognitive and Affective Mindfulness Scale-Revised – mindfulness trait assessment.
- **CARE-Express**: Cancer and Aging Reflections for Elders – Expressive Writing.
- **CARS: Concerns About Recurrence Scale** – 4-item fear of recurrence measure.
- **CBT: Cognitive Behavioral Therapy** – an evidence-based, structured psychotherapeutic approach focused on modifying dysfunctional thoughts, behaviors, and emotional responses through cognitive restructuring and behavioral techniques.
- **CCI: Coping and Communication-enhancing Intervention** – structured, CBT-based therapy targeting emotional coping, communication, and fear of recurrence.
- **CCS: Couple Communication Scale** – assesses dyadic communication quality.
- **CD-RISC-10**: Connor-Davidson Resilience Scale – 10 items.
- **CES-D: Center for Epidemiologic Studies Depression Scale** – screens for depressive symptoms.
- **CFI/RMSEA/SRMR**: Model fit indices.
- **CFS: Cancer Fatigue Scale** – 15-item tool for evaluating fatigue in cancer patients.
- **CI: Confidence Interval** – indicates the range of values within which the true effect likely lies.
- **CMS**: Constructed Meaning Scale – evaluates meaning-making in illness.
- **CNP: Chronic Neuropathic Pain** – long-term nerve pain often caused by chemotherap
- **CNS-VS: CNS Vital Signs** – computerized battery for objective neurocognitive testing.
- **Cochrane Risk of Bias 2 (RoB 2) Framework:** The Cochrane Risk of Bias 2 (RoB 2) tool is the current gold-standard methodology for assessing the risk of bias in randomized controlled trials (RCTs). Unlike earlier instruments that relied on numerical scoring systems, RoB 2 adopts a domain-based qualitative assessment approach. Each trial is evaluated across five predefined domains of potential bias, with judgments categorized as Low Risk of Bias, Some Concerns, or High Risk of Bias for each domain. The five domains assessed under RoB 2 are as follows:
    - D1: Bias arising from the randomization process;
    - D2: Bias due to deviations from intended interventions;
    - D3: Bias due to missing outcome data;
    - D4: Bias in measurement of the outcome;
    - D5: Bias in selection of the reported result.

  The overall risk of bias judgment is determined in accordance with guidance from the Cochrane Handbook for Systematic Reviews of Interventions. A study is rated as:
    - Low Risk of Bias if all five domains are individually judged to be at low risk;
    - Some Concerns if at least one domain raises concerns but none are classified as high risk;
    - High Risk of Bias if one or more domains are rated as high risk, or if multiple domains present issues that together reduce confidence in the study's validity.

  This comprehensive tool enables nuanced evaluations that inform evidence grading and inclusion decisions in systematic reviews and meta-analyses.
- **Cohen's d**: Standardized measure of effect size.

No.	ID	Study Design	RoB 2 D1 (Judgement/ Support)	RoB 2 D2 (Judgement/ Support)	RoB 2 D3 (Judgement/ Support)	RoB 2 D4 (Judgement/ Support)	RoB 2 D5 (Judgement/ Support)	Overall RoB 2 (Judgement/ Support)	GRADE Certainty (Judgement/ Support)

- **CONSORT (Consolidated Standards of Reporting Trials)**: A standardized set of recommendations for reporting randomized controlled trials. It improves transparency, reproducibility, and clarity of clinical trial reporting, ensuring essential elements like participant flow, randomization, and outcomes are clearly described.
- **COPE**: Coping Orientation to Problems Experienced Inventory.
- **Cortisol:** Hormone indicating physiological stress levels.
- **CREB:** cAMP Response Element-Binding Protein – transcription factor involved in stress signaling.
- **CRF: Cancer-Related Fatigue** – persistent, subjective sense of tiredness related to cancer or its treatment.
- **Cronbach α (Alpha)**: A statistic used to measure the internal consistency (reliability) of a psychometric instrument. It reflects how closely related a set of items are as a group. Values above 0.70 are generally considered acceptable, with >0.90 indicating excellent reliability.
- **CRP: C-Reactive Protein** – biomarker of systemic inflammation.
- **CSQ-8:** Client Satisfaction Questionnaire – measures patient satisfaction with care.
- **CTRU (Clinical Trials Research Unit):** A specialized academic unit (in this case, at the University of Leeds) responsible for trial design, data management, and oversight of randomization procedures to ensure methodological rigor and allocation concealment in clinical trials.
- **DADDS: Death and Dying Distress Scale** – measures anxiety related to dying and end-of-life.
- **DASS-21**: Depression Anxiety Stress Scales – Short Form.
- **DCS: Decisional Conflict Scale** – evaluates uncertainty in making health-related decisions, including perceived effectiveness of information and support.
- **DS: Demoralization Scale** – assesses loss of meaning and helplessness.
- **DT: Distress Thermometer** – A self-reported screening tool measuring psychological distress on a scale from 0 (none) to 10 (extreme).
- **Dual-role therapist bias**: A potential source of bias where the therapist is also a study investigator, potentially influencing outcomes due to allegiance or expectancy.
- **DUACRS:** Drexel University ACT Therapist Adherence and Competence Rating Scale.
- **ECog**: Everyday Cognition – assesses memory and cognitive impairment.
- **ECQ-R**: Emotional Control Questionnaire – Rehearsal Subscale.
- **ECR-M16: Modified Experiences in Close Relationships** – evaluates attachment insecurity.
- **ELISA: Enzyme-Linked Immunosorbent Assay** – quantifies cytokines (IL-6, IL-8).
- **EM Algorithm**: Expectation Maximization – an iterative method for imputing missing data.
- **eMBCT: Electronic delivery of MBCT** – self-paced, therapist-supported online version.
- **EORTC QLQ-C30:** European Organization for Research and Treatment of Cancer Quality of Life Questionnaire – measures cancer-specific QOL.
- **EPIC-26**: Expanded Prostate Cancer Index Composite.
- **EQ-5D**: EuroQol 5 Dimensions – utility measure for QALY estimation.
- **ESAS-FS: Edmonton Symptom Assessment Scale** – Financial-Spiritual; assesses symptom burden including distress, fatigue, depression, and anxiety.
- **ESWC**: Enhanced Social Work Control.
- **EWI**: Expressive Writing Intervention.
- **FACIT-Sp: Functional Assessment of Chronic Illness Therapy** – Spiritual Well-being scale.
- **FACT (Functional Assessment of Cancer Therapy)**: A multi-domain tool assessing cancer-specific QoL.

No.	ID	Study Design	RoB 2 D1 (Judgement/ Support)	RoB 2 D2 (Judgement/ Support)	RoB 2 D3 (Judgement/ Support)	RoB 2 D4 (Judgement/ Support)	RoB 2 D5 (Judgement/ Support)	Overall RoB 2 (Judgement/ Support)	GRADE Certainty (Judgement/ Support)

- **FACT-Cog**: **Functional Assessment of Cancer Therapy –** Cognitive Function – evaluates subjective cognitive complaints.
- **FACT-H&N**: Functional Assessment of Cancer Therapy – Head & Neck.
- **FANKIA**: Flow-cytometric Assay for NK-cell Immune Activity – measures cytotoxic NK cell performance.
- **FCRI**: Fear of Cancer Recurrence Inventory – measures recurrence-related anxiety.
- **FDR (False Discovery Rate):** A statistical method used to control for type I errors when multiple hypotheses are tested simultaneously.
- **FFMQ: Five Facet Mindfulness Questionnaire** – assesses mindfulness skills across five dimensions.
- **FIML: Full Information Maximum Likelihood** – a method used to estimate model parameters with missing data.
- **FSFI**: Female Sexual Function Index.
- **FSI:** Fatigue Symptom Inventory – primary outcome measure for fatigue interference and severity.
- **GAD-7 / PHQ-9:** Tools for assessing anxiety and depression severity, respectively.
- **GDP**: Guided Disclosure Protocol – structured therapeutic writing to process trauma.
- **GEE: Generalized Estimating Equations** – a statistical technique for repeated-measures data.
- **GP**: General Practitioner.
- **GR: Glucocorticoid Receptor** – anti-inflammatory pathway regulator.
- **GRADE**: Grading of Recommendations Assessment, Development and Evaluation. GRADE is a rigorous framework designed to evaluate the certainty (or quality) of evidence in systematic reviews, health technology assessments, and clinical guidelines. Unlike tools that assess individual studies (e.g., NOS or RoB 2), GRADE appraises the confidence in the body of evidence for each outcome across all relevant studies. GRADE starts with an initial rating depending on study design: Randomized trials → High certainty; Observational studies → Low certainty. Certainty can be downgraded based on the following five domains: Risk of Bias: Serious methodological flaws in included studies; Inconsistency: Substantial variability or heterogeneity in results across studies; Indirectness: Differences in population, intervention, comparator, or outcomes from the question of interest; Imprecision: Wide confidence intervals, small sample sizes, or few events reducing confidence in the effect estimate; Publication Bias: Evidence of selective reporting or failure to publish relevant studies. GRADE Levels of Certainty – Interpretation: High (We are highly confident that the true effect lies close to that of the estimate); Moderate (We believe the effect is likely close to the estimate, but there is a possibility it is substantially different); Low (Our confidence is limited: the true effect may be substantially different from the estimate); and Very Low (We have very little confidence in the effect estimate; the true effect is likely substantially different).
- **GSES: General Self-Efficacy Scale** – assesses perceived ability to cope with difficult situations.
- **HADS (Hospital Anxiety and Depression Scale):** A widely used, validated 14-item questionnaire that assesses anxiety (7 items) and depression (7 items) symptoms in medical patients.
- **HAIQ**: Hopelessness Assessment in Illness Questionnaire.
- **HE**: Health Education – control condition involving general educational sessions without psychotherapeutic elements, used to control for attention and group interaction.
- **HHI**: Herth Hope Index – assesses dimensions of hope, primary outcome.
- **HOPE**: Dispositional Hope Scale.
- **ICER:** Incremental Cost-Effectiveness Ratio – cost per additional effect.
- **IES-R: Impact of Event Scale-Revised** – a validated 22-item self-report scale assessing symptoms of post-traumatic stress, including intrusion, avoidance, and hyperarousal.
- **IIEF**: International Index of Erectile Function.
- **IL-6: Interleukin-6** – pro-inflammatory cytokine linked to stress and cancer outcomes.

No.	ID	Study Design	RoB 2 D1 (Judgement/ Support)	RoB 2 D2 (Judgement/ Support)	RoB 2 D3 (Judgement/ Support)	RoB 2 D4 (Judgement/ Support)	RoB 2 D5 (Judgement/ Support)	Overall RoB 2 (Judgement/ Support)	GRADE Certainty (Judgement/ Support)

- **IMAGE-HN**: Inventory to Measure and Assess imaGe disturbancE – Head and Neck.
- **iMBCT: Internet-based Mindfulness-Based Cognitive Therapy** – a digital adaptation of MBCT for remote delivery with therapist feedback.
- **IMCP (Individual Meaning-Centered Psychotherapy):** A manualized, structured psychotherapeutic intervention derived from existential logotherapy, developed specifically for patients with advanced cancer to enhance a sense of meaning, purpose, and spiritual well-being.
- **IPAQ: International Physical Activity Questionnaire** – captures levels of physical activity across multiple domains.
- **IPC**: Internal-Powerful Others-Chance Scales – locus of control.
- **IPT: Interpersonal Therapy** – a time-limited, evidence-based psychotherapy focused on improving interpersonal functioning and resolving current relational problems to reduce psychological symptoms, especially depression.
- **IRB:** Institutional Review Board – body that reviews ethical aspects of clinical studies.
- **IRCT**: Iranian Registry of Clinical Trials.
- **ITT (Intention-To-Treat)**: A principle in trial analysis where all participants are included in the group to which they were randomized, regardless of whether they completed the intervention as planned.
- **K6: Kessler Psychological Distress Scale** – a validated 6-item screening tool for depression and anxiety symptoms.
- **Kabat-Zinn (Jon Kabat-Zinn)**: A professor emeritus of medicine and the founder of the Mindfulness-Based Stress Reduction (MBSR) program at the University of Massachusetts Medical School. He developed MBSR in the late 1970s as an 8-week structured program combining mindfulness meditation, body awareness, and yoga to support individuals coping with stress, pain, and illness. His protocol is the standard framework for most clinical mindfulness interventions used in research today.
- **LILAC**: Lessons in Linking Affect and Coping – 5-session intervention teaching 8 skills to boost positive affect.
- **LIWC**: Linguistic Inquiry and Word Count
- **LMM: Linear Mixed Models** – statistical approach accounting for both fixed and random effects in repeated measures.
- **LOT-R**: Life Orientation Test–Revised (optimism).
- **LSC-R: Life Stressor Checklist-Revised** – an inventory assessing lifetime exposure to stressful events and their perceived impact.
- **MA**: Master's level.
- **MAPS: Mindful Awareness Practices** – structured program developed at UCLA for mindfulness cultivation.
- **MAAS**: Mindful Attention Awareness Scale – measures dispositional mindfulness.
- **MAR (Missing At Random)**: A statistical assumption in which the probability of missing data on a variable is related to other observed data but not to the value of the variable itself. Under MAR, methods like FIML and EM can yield unbiased parameter estimates if model assumptions are met.
- **MCMQ:** Medical Coping Modes Questionnaire – evaluates coping strategies (facing, avoidance, yielding).
- **MCPC**: Meaning-Centered Pain Coping Skills Training.
- **MBCT: Mindfulness-Based Cognitive Therapy** – an integrative approach combining mindfulness training with cognitive-behavioral techniques to prevent relapse in depression and alleviate psychological distress.
- **MBI-TAC**: Mindfulness-Based Interventions – Teachers Assessment Criteria – rates instructor competency.
- **MBSR: Mindfulness-Based Stress Reduction** – an eight-week, standardized intervention incorporating mindfulness meditation and yoga to reduce stress, anxiety, and somatic symptoms.
- **MBSR-CRF**: Mindfulness-Based Stress Reduction adapted specifically for cancer-related fatigue.

No.	ID	Study Design	RoB 2 D1 (Judgement/ Support)	RoB 2 D2 (Judgement/ Support)	RoB 2 D3 (Judgement/ Support)	RoB 2 D4 (Judgement/ Support)	RoB 2 D5 (Judgement/ Support)	Overall RoB 2 (Judgement/ Support)	GRADE Certainty (Judgement/ Support)

- **MCAR:** Missing Completely At Random – ideal scenario for missing data handling.
- **MCID: Minimal Clinically Important Difference** – threshold for meaningful symptom improvement.
- **MCMQ:** Medical Coping Modes Questionnaire – evaluates coping strategies (facing, avoidance, yielding).
- **MDASI:** M.D. Anderson Symptom Inventory – symptom interference and severity.
- **MHC-SF:** Mental Health Continuum – Short Form – gauges overall mental health.
- **MICE: Multiple Imputation by Chained Equations** – advanced missing data handling method.
- **MLM: Multilevel Modeling** – statistical technique used for repeated measures over time.
- **MMAS: Morisky Medication Adherence Scale** – 8-item tool for medication compliance.
- **MOS-SSS**: Medical Outcomes Study – Social Support Survey.
- **MQoL-C:** Multidimensional Quality of Life Scale – Cancer Version – evaluates cancer-related well-being.
- **MSAS: Memorial Symptom Assessment Scale –** measures physical and psychological symptom distress
- **MSPSS**: **Multidimensional Scale of Perceived Social Support** – measures perceived emotional and practical support.
- **MT: Morita Therapy** – Japanese acceptance-based therapy aimed at tolerating unpleasant feelings and promoting purposeful action.
- **MUIS**: Mishel Uncertainty in Illness Scale – measures uncertainty due to illness progression or treatment.
- **Multiple imputation:** A statistical technique used to handle missing data by creating multiple complete datasets and combining results to reduce bias.
- **NF-κB: Nuclear Factor kappa**-light-chain-enhancer of activated B cells – transcription factor regulating inflammation.
- **NPSI: Neuropathic Pain Symptom Inventory** – specific neuropathic symptom quantification.
- **NT: Naikan Therapy** – Japanese introspective psychotherapy focusing on gratitude and self-reflection via structured questioning.
- **Nuevo Amanecer**: Peer-delivered CBT-based intervention for Spanish-speaking Latinas
- **OR: Odds Ratio** – a measure of association between exposure and outcome.
- **OSF: Open Science Framework** – platform for preregistering analysis plans.
- **PAIS-SR**: Psychosocial Adjustment to Illness Scale – Self Report.
- **PedsQL**: Pediatric Quality of Life Inventory.
- **PHQ-8**: 8-item depression scale from Patient Health Questionnaire – assesses depressive symptoms.
- **PHQ-9: Patient Health Questionnaire-9** – standard depression screening and severity measure.
- **PHQ-15:** Patient Health Questionnaire – Somatic Symptoms.
- **POMS**: Profile of Mood States – mood-related symptoms.
- **PRISM: Promoting Resilience in Stress Management** – a structured psychosocial intervention designed to strengthen resilience and adaptive coping skills in patients with serious medical illness, including cancer.
- **PRMQ:** Prospective and Retrospective Memory Questionnaire – assesses daily memory failures.
- **Procedural fidelity checklist**: Measures how closely intervention delivery adheres to the study protocol.
- **PROC MIXED**: SAS Procedure for Mixed Models.
- **PROMIS CATs**: Patient-Reported Outcomes Measurement Information System Computer Adaptive Tests – adaptive assessments across multiple health domains.

No.	ID	Study Design	RoB 2 D1 (Judgement/ Support)	RoB 2 D2 (Judgement/ Support)	RoB 2 D3 (Judgement/ Support)	RoB 2 D4 (Judgement/ Support)	RoB 2 D5 (Judgement/ Support)	Overall RoB 2 (Judgement/ Support)	GRADE Certainty (Judgement/ Support)

- **PROMIS GSSL**: Patient-Reported Outcomes Measurement Information System – Global Satisfaction With Sex Life.
- **PSS: Perceived Stress Scale** – assesses perceived stress over the past month.
- **PSQI: Pittsburgh Sleep Quality Index** – a validated questionnaire assessing subjective sleep quality.
- **PTGI: Posttraumatic Growth Inventory** – A validated questionnaire assessing positive psychological changes following trauma.
- **QALY: Quality-Adjusted Life Year** – combines quantity and quality of life.
- **QoL (Quality of Life):** A multidimensional concept reflecting individuals' overall well-being, encompassing physical, psychological, social, and functional aspects of health, especially important in cancer survivorship and treatment evaluation contexts.
- **QOL Index-III: Quality of Life Index** – evaluates satisfaction across health, social, and psychological domains.
- **QUAL-EC: Quality of Life at the End of Life Cancer Scale** – assesses end-of-life preparation, healthcare provider relationships, and life completion.
- **RAND-36:** 36-item generic health-related quality of life questionnaire.
- **RCT**: Randomized Controlled Trial.
- **REDCap**: Research Electronic Data Capture
- **RRQ**: Rumination and Reflection Questionnaire – measures repetitive negative thinking.
- **RT: Relaxation Training** – intervention focused on reducing anxiety and promoting calm through techniques such as progressive muscle relaxation, guided imagery, and breathing exercises.
- **SC: Supportive Counseling** – nondirective intervention emphasizing emotional expression and validation without skills teaching.
- **SCS-SF:** Self-Compassion Scale – Short Form – assesses self-kindness and emotional regulation.
- **SD**: Standard Deviation.
- **SDS/SAS: Self-Rating Depression/Anxiety Scales** – assess mood disturbances on a 4-point Likert scale.
- **Self-reported measures**: Outcomes based on patient responses, often used in psychosocial interventions; while valuable, they are vulnerable to expectation and social desirability biases.
- **SEM: Structural Equation Modeling** – statistical technique used to evaluate relationships between observed and latent variables.
- **SF-12:** 12-item Short Form Health Survey – evaluates physical and mental health-related quality of life.
- **SF-36 Vitality**: 4-item subscale of SF-36 – measures energy and fatigue.
- **SII**: Symptom Impact Inventory.
- **SMEW**: Self-Regulation Model of Expressive Writing.
- **SOC-13:** Sense of Coherence 13-item Scale – measures resilience and comprehensibility/manageability of stress.
- **STAI-S:** State-Trait Anxiety Inventory (State subscale) – assesses transient anxiety levels.
- **STAI-Y: State-Trait Anxiety Inventory (Form Y) –** widely used to assess state anxiety.
- **Stroop Test**: Measures cognitive flexibility and selective attention by evaluating response to congruent/incongruent stimuli.
- **T1/T2:** Assessment timepoints – 3 months and 6 months post-baseline.
- **TAS-20**: Toronto Alexithymia Scale – 20 items.
- **TELiS: Transcription Element Listening System** – bioinformatics tool for transcription factor activity inference.
- **TES: Targeted selection, Enhanced care, and Stepped care** – multilevel psychological intervention for distress in metastatic CRC.
- **TIDieR (Template for Intervention Description and Replication):** A standardized checklist to improve the completeness and replicability of intervention reporting.

No.	ID	Study Design	RoB 2 D1 (Judgement/ Support)	RoB 2 D2 (Judgement/ Support)	RoB 2 D3 (Judgement/ Support)	RoB 2 D4 (Judgement/ Support)	RoB 2 D5 (Judgement/ Support)	Overall RoB 2 (Judgement/ Support)	GRADE Certainty (Judgement/ Support)

- **TrueNTH:** Global initiative supporting men's health and cancer survivorship.
- **UC**: Usual Care – non-intervention comparator group.
- **UCLA Loneliness**: UCLA Loneliness Scale.
- **UCSF**: University of California, San Francisco.
- **WCC**: Ways of Coping Checklist – measures coping strategies.
- **WHOQOL-BREF:** World Health Organization Quality of Life (26-item version).
- **WOCQ**: Ways of Coping Questionnaire.
- **ZEFB: Zero-Effect from Baseline** – imputation method assuming no change in missing data.

References: All studies referenced herein are fully cited within the main body of the manuscript.

Table S6. Structured Psychotherapeutic Interventions – Effect Estimates (Forest Plot).

Meta-Analysis - Structured Psychotherapeutic Interventions (Forest Plot)												
**No.**	**ID**	**Design**	**Experimental**			**Control**			**Weight**	**SMD**	**t**	**p-value**
			**Mean**	**SD**	**Total**	**Mean**	**SD**	**Total**	**(%)**	**(95%CI)**		
**1**	Breitbart et al., 2018, USA	RCT	7.33	1.7	78	6.49	1.5	60	6.80	0.52 [0.175, 0.859]		
**2**	Fauser et al., 2023, Germany	RCT	7.3	2.68	80	7.6	2.68	80	6.84	-0.11 [-0.422, 0.199]		
**3**	Graham et al., 2024, UK	RCT	88.1	9.34	33	81.4	10.10	34	6.57	0.68 [0.187, 1.174]		
**4**	Gudenkauf et al., 2015, USA	RCT	85	9.62	42	80	9.62	45	6.68	0.52 [0.087, 0.943]		
**5**	Han et al., 2021, China	RCT	2.42	1.03	55	4.81	1.43	53	6.63	-1.91 [-2.368, -1.452]		
**6**	Huang et al., 2024, China	RCT	0.714	1.4	70	4.5	1.0	69	6.57	-3.09 [-3.588, -2.595]		
**7**	Isaka et al., 2021, Japan	RCT	15.96	9.32	423	17.91	7.73	407	6.99	-0.23 [-0.364, -0.091]		
**8**	Li et al., 2023, China	RCT	9.84	2.57	48	10.98	2.87	48	6.72	-0.16 [-0.557, 0.245]		
**9**	Lopez et al., 2023, USA	RCT	6.94	2.64	17	8.67	2.31	18	6.20	-0.68 [-1.367, -0.001]		
**10**	Manne et al., 2017, USA	RCT	7.94	7.39	118	10.81	7.11	116	6.89	-0.39 [-0.653, -0.136]		
**11**	Marziliano et al., 2023, USA	RCT	16.09	10.25	197	14.37	8.73	191	6.95	0.18 [–0.019, 0.38]		
**12**	Nissen et al., 2020, Denmark	RCT	10.7	6.6	73	15.6	9.2	38	6.72	-0.64 [-1.043, -0.24]		
**13**	Park et al., 2020, Japan	RCT	6.18	4.83	38	14.00	8.00	36	6.57	-1.18 [-1.674, -0.683]		
**14**	Rodin et al., 2018, Canada	RCT	5.35	3.99	107	6.64	4.97	118	6.89	-0.28 [-0.547, -0.021]		
**15**	Ross et al., 2016, USA	RCT	15.38	6.33	14	23.00	7.74	15	5.99	-1.04 [-1.827, -0.261]		
	**Random Effects Model**	REM	19.016	5.797	1393	20.452	5.666	1328	100	-0.51 [-1.057, 0.039]	-1.99	0.06629
	**Prediction Interval**	PI								[-2.66, 1.64]		

Table S7. Assessment of Heterogeneity in Randomized Controlled Trials Using Funnel Plot Analysis (Structured Psychotherapeutic Interventions).

**Heterogeneity (Structured Psychotherapeutic Interventions)**			
**No.**	Parameter	Value	95% CI
**1**	Tau^2	0.92	0.472-2.399
**2**	Tau	0.96	0.687-1.549
**3**	I^2	0.95	0.927-0.962
**4**	H	4.35	3.697-5.109
**Test of Overall Efect: t_{14} = -1.99 (p = 0.07)**			

Table S8. Cochran's Q Test for Heterogeneity in Randomized Controlled Trials: Funnel Plot-Based Analysis (Structured Psychotherapeutic Interventions).

**Cochran's Q Test (Structured Psychotherapeutic Interventions)**			
**No.**	Q	d.f.	p-value
**1**	264.45	14	< 0.01

Table S9. Mindfulness-Based and Stress Reduction Interventions – Effect Estimates (Forest Plot).

Meta-Analysis - Mindfulness and Stress Reduction Interventions (Forest Plot)												
**No.**	**ID**	**Design**	**Experimental**			**Control**			**Weight**	**SMD**	**t**	**p-value**
			**Mean**	**SD**	**Total**	**Mean**	**SD**	**Total**	**(%)**	**(95%CI)**		
**1**	Bagherzadeh et al., 2022, Iran	RCT	40.36	10.28	22	45.63	12.54	24	7.14	-0.45 [−1.036, 0.137]		
**2**	Bower et al., 2015, USA	RCT	9.99	10.24	39	18.47	10.18	32	7.59	-0.82 [-1.309, -0.334]		
**3**	Cillessen et al., 2018, Netherlands	RCT	12.6	6.7	120	9.6	6.4	125	8.45	0.46 [0.203, 0.71]		
**4**	Duval et al., 2022, Canada	RCT	44.14	11.75	30	42.81	12.52	30	7.51	0.11 [−0.398, 0.615]		
**5**	Gu et al., 2024, China	RCT	31.25	1.59	40	36.53	4.24	38	7.46	-1.65 [-2.166, -1.132]		
**6**	Johns et al., 2015, USA	RCT	4.58	4.18	18	10.03	4.18	17	6.45	-1.27 [−2.008, −0.54]		
**7**	Johns et al., 2016, USA	RCT	64.64	15.39	35	52.28	14.90	36	7.60	0.81 [0.322, 1.292]		
**8**	Kenne et al., 2017, Sweden	RCT	3.3	3.3	62	3.8	3.8	52	8.07	-0.14 [−0.509, 0.229]		
**9**	Lengacher et al., 2016, USA	RCT	8.12	5.45	154	8.82	6.05	146	8.52	-0.12 [−0.348, 0.105]		
**10**	Mirmahmoodi et al., 2020, Iran	RCT	23.50	11.35	22	35.00	13.52	22	6.97	-0.90 [−1.528, −0.282]		
**11**	Reich et al., 2017, USA	RCT	8.12	5.45	154	8.82	6.05	146	8.52	-0.12 [−0.348, 0.105]		
**12**	Shergill et al., 2022, Canada	RCT	7.61	4.73	49	7.85	5.34	49	7.97	-0.05 [−0.443, 0.349]		
**13**	Victorson et al., 2020, USA	RCT	53.65	8.15	35	54.15	9.23	42	7.75	-0.06 [−0.505, 0.392]		
	**Random Effect Model**	REM	23.989	7.581	780	25.676	8.380	759	100	-0.29 [-0.7, 0.111]	-1.58	0.13933
	**Prediction Interval**	PI								[-1.71, 1.12]		

Table S10. Assessment of Heterogeneity in Randomized Controlled Trials Using Funnel Plot Analysis (Mindfulness and Stress Reduction Interventions).

Heterogeneity (Mindfulness and Stress Reduction Interventions)			
**No.**	**Parameter**	**Value**	**95% CI**
**1**	Tau^2	0.38	0.17-1.199
**2**	Tau	0.62	0.412-1.095
**3**	I^2	0.87	0.797-0.918
**4**	H	2.79	2.221-3.496
**Test of Overall Efect: t_{12} = -1.58 ($p = 0.14$)**			

Table S11. Cochran's Q Test for Heterogeneity in Randomized Controlled Trials: Funnel Plot-Based Analysis (Mindfulness and Stress Reduction Interventions).

Cochran's Q Test (Mindfulness and Stress Reduction Interventions)			
**No.**	**Q**	**d.f.**	**p-value**
**1**	93.19	12	< 0.01

Table S12. Coping and Psychological Resilience Interventions – Effect Estimates (Forest Plot).

Meta-Analysis - Coping and Psychological Resilience Interventions (Forest Plot)												
**No.**	**ID**	**Design**	**Experimental**			**Control**			**Weight**	**SMD**	**t**	**p-value**
			**Mean**	**SD**	**Total**	**Mean**	**SD**	**Total**	**(%)**	**(95%CI)**		
**1**	Cafaro et al., 2024 Italy	RCT	48.3	23.4	39	51.5	25.4	42	7.37	-0.13 [−0.566, 0.307]		
**2**	Cheung et al., 2017, USA	RCT	11.61	10.39	18	17.10	10.40	10	5.71	-0.51 [−1.299, 0.2674]		
**3**	Graboyes et al., 2023, USA	RCT	12.2	5.5	20	16.5	6.0	24	6.54	-0.73 [−1.345, −0.116]		
**4**	Jensen-Johansen et al., 2018, Denmark	RCT	7.00	3.88	201	6.62	4.05	226	8.24	0.10 [−0.095, 0.286]		
**5**	Lu et al., 2023, USA	RCT	8.15	5.56	54	10.87	6.51	36	7.41	-0.45 [−0.88, −0.026]		
**6**	Nairn and Merluzzi, 2019, USA	RCT	5.34	3.71	66	5.51	4.57	68	7.77	-0.04 [−0.379, 0.298]		
**7**	Nelson et al., 2021, USA	RCT	4.2	3.9	86	5.7	4.1	74	7.87	-0.37 [−0.687, −0.06]		
**8**	Rosenberg et al., 2018, USA	RCT	5.28	2.5	36	6.05	2.5	38	7.28	-0.30 [−0.763, 0.154]		
**9**	Rosenberg et al., 2021, USA	RCT	7.10	4.86	29	7.20	4.86	28	7.00	-0.02 [−0.54, 0.499]		
**10**	Samami et al., 2021, Iran	RCT	8.22	2.95	27	17.26	4.28	30	6.17	-2.40 [−3.094, −1.711]		
**11**	Santoyo-Olsson et al., 2022, USA	RCT	0.97	0.86	76	1.50	0.90	75	7.82	-0.60 [−0.925, −0.273]		
**12**	Tutino et al., 2022, USA	RCT	4.00	2.1	21	7.10	2.7	27	6.48	-1.24 [−1.867, −0.615]		
**13**	Winger et al., 2023, USA	RCT	6.69	4.85	29	7.85	4.85	30	7.03	-0.24 [−0.748, 0.276]		
**14**	Wittmann et al., 2022, USA	RCT	53.00	5.0	31	51.00	5.0	51	7.31	0.40 [−0.054, 0.847]		
	**Random Effects Model**	REM	13.004	5.675	733	15.125	6.151	759	100	-0.43 [-0.81, -0.054]	-2.47	0.02832
	**Prediction Interval**	PI								[-1.74, 0.88]		

Table S13. Assessment of Heterogeneity in Randomized Controlled Trials Using Funnel Plot Analysis (Coping and Psychological Resilience Interventions).

**Heterogeneity (Coping and Psychological Resilience Interventions)**			
**No.**	Parameter	Value	95% CI
**1**	Tau^2	0.33	0.15-1.115
**2**	Tau	0.58	0.387-1.056
**3**	I^2	0.83	0.736-0.896
**4**	H	2.46	1.945-3.106
**Test of Overall Efect: t_{13} = -2.47 (p = 0.03)**			

Table S14. Cochran's Q Test for Heterogeneity in Randomized Controlled Trials: Funnel Plot-Based Analysis (Coping and Psychological Resilience Interventions).

**Cochran's Q Test (Coping and Psychological Resilience Interventions)**			
**No.**	Q	d.f.	p-value
**1**	78.53	13	< 0.01

Table S15. Coping and Psychological Resilience Interventions (Trimm and Fill Plots).

Meta-Analysis - Coping and Psychological Resilience Interventions (Trimm and Fill Plots)										
**No.**	**ID**	**Design**	**Experimental**			**Control**	**Weight**	**SMD**	**t**	**p-value**
			**SMD**	**SE**	**Total**	**Total**	**(%)**	**(95%CI)**		
**1**	Cafaro et al., 2024 Italy	RCT	-0.1296	0.2226	39	42	5.4	-0.13 [−0.566, 0.307]		
**2**	Cheung et al., 2017, USA	RCT	-0.5128	0.4012	18	10	4.7	-0.51 [−1.299, 0.2674]		
**3**	Graboyes et al., 2023, USA	RCT	-0.7307	0.3135	20	24	5.1	-0.73 [−1.345, −0.116]		
**4**	Jensen-Johansen et al., 2018, Denmark	RCT	0.0955	0.0970	201	226	5.7	0.10 [−0.095, 0.286]		
**5**	Lu et al., 2023, USA	RCT	-0.4528	0.2179	54	36	5.4	-0.45 [−0.88, −0.026]		
**6**	Nairn and Merluzzi, 2019, USA	RCT	-0.0405	0.1728	66	68	5.6	-0.04 [−0.379, 0.298]		
**7**	Nelson et al., 2021, USA	RCT	-0.3738	0.1600	86	74	5.6	-0.37 [−0.687, −0.06]		
**8**	Rosenberg et al., 2018, USA	RCT	-0.3048	0.2340	36	38	5.4	-0.30 [−0.763, 0.154]		
**9**	Rosenberg et al., 2021, USA	RCT	-0.0203	0.2650	29	28	5.3	-0.02 [−0.54, 0.499]		
**10**	Samami et al., 2021, Iran	RCT	-2.4025	0.3529	27	30	4.9	-2.40 [−3.094, −1.711]		
**11**	Santoyo-Olsson et al., 2022, USA	RCT	-0.5992	0.1665	76	75	5.6	-0.60 [−0.925, −0.273]		
**12**	Tutino et al., 2022, USA	RCT	-1.2409	0.3195	21	27	5.1	-1.24 [−1.867, −0.615]		
**13**	Winger et al., 2023, USA	RCT	-0.2360	0.2614	29	30	5.3	-0.24 [−0.748, 0.276]		
**14**	Wittmann et al., 2022, USA	RCT	0.3962	0.2299	31	51	5.4	0.40 [−0.054, 0.847]		
	**Cheung et al., 2017, USA**	Filled	0.3745	0.4012	18	10	4.7	0.37 [-0.410, 1.160]		
	**Santoyo-Olsson et al., 2022, USA**	Filled	0.4609	0.1665	76	75	5.6	0.46 [0.130, 0.790]		
	**Graboyes et al., 2023, USA**	Filled	0.5924	0.3135	20	24	5.1	0.59 [-0.020, 1.210]		
	**Tutino et al., 2022, USA**	Filled	1.1026	0.3195	21	27	5.1	1.10 [0.480, 1.730]		
	**Samami et al., 2021, Iran**	Filled	2.2642	0.3529	27	30	4.9	2.26 [1.570, 2.960]		
	**Random Effects Model**	REM			895	925	100	-0.09 [-0.54, 0.35]	-0.44	0.66
	**Prediction Interval**	PI						[-1.95, 1.77]		

Table S16. Assessment of Heterogeneity in Randomized Controlled Trials Using Trimm and Fill Plots Analysis (Coping and Psychological Resilience Interventions).

**Heterogeneity (Coping and Psychological Resilience Interventions)**		
**No.**	Parameter	Value
**1**	Tau^2	0.7361
**2**	Tau	0.8579
**3**	I^2	0.89
**Test of Overall Efect: t_{18} = -0.44 (p = 0.66)**		

Table S17. Cochran's Q Test for Heterogeneity in Randomized Controlled Trials: Trimm and Fill Plots-based Analysis (Coping and Psychological Resilience Interventions).

**Cochran's Q Test (Coping and Psychological Resilience Interventions)**			
**No.**	Q	d.f.	p-value
**1**	161.22	18	< 0.01

Table S18. Meta-Subgroup Analysis – CBT-Based Interventions.

Meta-Subgroup Analysis (CBT-Based Interventions)										
**No.**	**ID**	**Design (Intervention)**	**Experimental Mean (SD)**	**N (Exp)**	**Control Mean (SD)**	**N (Ctrl)**	**SMD (95% CI)**	**Weight (%)**	**t**	**p-value**
**1**	Gudenkauf et al., 2015, USA	RCT (CBT-Based)	85.00 (9.62)	42	80.00 (9.62)	45	0.52 [0.087, 0.943]	25.96		
**2**	Li et al., 2023, China	RCT (CBT-Based)	9.84 (2.57)	48	10.98 (2.87)	48	-0.41 [- 0.82, - 0.011]	26.22		
**3**	Lopez et al., 2023, USA	RCT (CBT-Based)	6.94 (2.64)	17	8.67 (2.31)	18	-0.68 [-1.367, - 0.001]	22.66		
**4**	Park et al., 2020, Japan	RCT (CBT-Based)	6.18 (4.83)	38	14.00 (8.00)	36	-1.18 [-1.674, -0.683]	25.16		
	**Random Effects Model**	REM	26.99 (4.915)	145	28.4125 (5.7)	147	-0.43 [-1.57, 0.717]	100.00	-1.19	0.3209
	**Prediction Interval**	PI					[-3.74, 2,89]			

Table S19. Assessment of Heterogeneity in Randomized Controlled Trials Using Funnel Plot Analysis (CBT-Based Interventions).

Heterogeneity (CBT-Based Interventions)			
**No.**	Parameter	Value	95% CI
**1**	Tau²	0.46	0.106-6.976
**2**	Tau	0.68	0.326-2.641
**3**	I²	0.89	0.747-0.953
**4**	H	3.03	1.989-4.6
**Test of Overall Efect: t_3 = - 1.19 (p = 0.32)**			

Table S20. Cochran's Q Test for Heterogeneity in Randomized Controlled Trials: Funnel Plot-Based Analysis (CBT-Based Interventions).

Cochran's Q Test (CBT-Based Interventions)			
**No.**	Q	d.f.	p-value
**1**	27.44	3	< 0.01

Table S21. Meta-Subgroup Analysis – Supportive/Expressive Interventions.

Meta-Subgroup Analysis (Supportive/Expressive Interventions)										
**No.**	**Study**	**Design (Intervention)**	**Experimental Mean (SD)**	**N (Exp)**	**Control Mean (SD)**	**N (Ctrl)**	SMD **(95% CI)**	Weight (%)	t	p-value
**1**	Han et al., 2021, China	RCT (Supportive/Expressive)	2.42 (1.03)	55	4.81 (1.43)	53	-1.91 [-2.368, -1.452]	25.51		
**2**	Huang et al., 2024, China	RCT (Supportive/Expressive)	0.714 (1.40)	70	4.50 (1.00)	69	-3.09 [-3.588, -2.595]	25.30		
**3**	Nissen et al., 2020, Denmark	RCT (Supportive/Expressive)	10.70 (6.60)	73	15.60 (9.20)	38	-0.64 [-1.043, -0.24]	25.78		
**4**	Ross et al., 2016, USA	RCT (Supportive/Expressive)	15.38 (6.33)	14	23.00 (7.74)	15	-1.04 [-1.827, -0.261]	23.41		
	Random Effect Model	REM	7.3035 (3.84)	212	11.9775 (4.8425)	175	-1.68 [-3.413, 0.05]	100.00	-3.08	0.05403
	Prediction Interval	PI					[-6.81, 3.45]			

Table S22. Assessment of Heterogeneity in Randomized Controlled Trials Using Funnel Plot Analysis (Supportive/Expressive Interventions).

Heterogeneity (Supportive/Expressive Interventions)			
**No.**	Parameter	Value	95% CI
**1**	Tau²	1.12	0.312-16.279
**2**	Tau	1.06	0.558-4.035
**3**	I²	0.95	0.902-0.975
**4**	H	4.48	3.2-6.27
**Test of Overall Efect: t_3 = -3.08 (p = 0.05)**			

Table S23. Cochran's Q Test for Heterogeneity in Randomized Controlled Trials: Funnel Plot-Based Analysis (Supportive/Expressive Interventions).

Cochran's Q Test (Supportive/Expressive Interventions)			
**No.**	Q	d.f.	p-value
**1**	60.19	3	< 0.01

Table S24. Statistical Comparison Between CBT-Based and Supportive/Expressive Interventions (Subgroup Difference Test).

Subgroup Difference Test (CBT-Based vs. Supportive/Expressive Interventions)				
**Comparison**	**SMD Difference (Δ)**	**Standard Error (SE)**	**Z-score**	**p-value**
**CBT vs. Supportive/Expressive**	1.25	1.06	1.18	0.238

Notes:

- **SMD Difference (Δ)**: represents the difference between the pooled standardized mean differences (Hedges' g) of the CBT-based and Supportive/Expressive subgroups.
- **Standard Error (SE):** reflects the combined uncertainty of the two pooled SMDs, computed using the standard formula for the pooled standard error of independent estimates.
- **Z-score**: is the test statistic derived from the ratio of the SMD difference to its standard error, used to assess statistical significance.
- **p-value**: indicates the probability of observing such a difference (or more extreme) under the null hypothesis of no true difference between the subgroups. A p-value < 0.05 is typically considered statistically significant.
- The test was conducted using a normal distribution approximation and assumes independence between subgroup estimates.

Table S25. Effect Sizes for Meta-Regression Analysis of Structured Psychotherapeutic Interventions in Oncology (Hedges' g).

Meta-Regression Analysis: Structured Psychotherapeutic Interventions as Moderator						
**No.**	**ID**	**Intervention Type**	**Experimental Mean (N)**	**Control Mean (N)**	**SMD (Hedges' g)**	**SE**
**1**	Gudenkauf et al., 2015	CBT	85.00 (42)	80.00 (45)	0.52	0.03
**2**	Li et al., 2023	CBT	9.84 (48)	10.98 (48)	-0.42	0.21
**3**	Lopez et al., 2023	CBT	6.94 (17)	8.67 (18)	-0.69	0.35
**4**	Park et al., 2020	CBT	6.18 (38)	14.00 (36)	-1.18	0.26
**5**	Han et al., 2021	Supportive	2.42 (55)	4.81 (53)	-1.91	0.24
**6**	Huang et al., 2024	Supportive	0.714 (70)	4.50 (69)	-3.09	0.25
**7**	Nissen et al., 2020	Supportive	10.70 (73)	15.60 (38)	-0.65	0.21
**8**	Ross et al., 2016	Supportive	15.38 (14)	23.00 (15)	-1.04	0.41
**9**	Graham et al., 2024	ACT	88.10 (33)	81.40 (34)	0.53	0.25
**10**	Zhang et al., 2022	ACT	22.35 (30)	23.81 (31)	-0.39	0.23
**11**	Cafaro et al., 2024	ACT	15.50 (67)	17.10 (66)	-0.25	0.24
**12**	Lu et al., 2023	ACT	13.50 (55)	15.00 (52)	-0.43	0.24
**13**	Fauser et al., 2023	Other	7.30 (80)	7.60 (80)	-0.11	0.16
**14**	Manne et al., 2017	Other	7.94 (118)	10.81 (116)	-0.40	0.13
**15**	Rodin et al., 2018	Other	5.35 (107)	6.64 (118)	-0.29	0.14

Notes:

- Hedges' g represents the standardized mean difference (SMD), adjusted for small sample bias.
- SE refers to the standard error of Hedges' g, calculated from the 95% confidence interval.
- Intervention types are coded as follows:
  - CBT: Cognitive Behavioral Therapy, a structured, skills-based intervention targeting dysfunctional thoughts and behaviors.
  - ACT: Acceptance and Commitment Therapy, a third-wave cognitive-behavioral intervention focused on psychological flexibility, mindfulness, and values-based action.
  - Supportive: Supportive/Expressive Psychotherapy, including interventions centered on emotional expression, interpersonal support, and meaning-making, typically in a structured format.
  - Other: Other structured psychotherapeutic approaches not classified under CBT, ACT, or Supportive categories (e.g., integrative, problem-solving, or novel hybrid models).
- Experimental and control values represent post-intervention mean scores with corresponding sample sizes in parentheses.
